# A multigene phylogeny toward a new phylogenetic classification of *Leotiomycetes*

**DOI:** 10.1186/s43008-019-0002-x

**Published:** 2019-06-07

**Authors:** Peter R. Johnston, Luis Quijada, Christopher A. Smith, Hans-Otto Baral, Tsuyoshi Hosoya, Christiane Baschien, Kadri Pärtel, Wen-Ying Zhuang, Danny Haelewaters, Duckchul Park, Steffen Carl, Francesc López-Giráldez, Zheng Wang, Jeffrey P. Townsend

**Affiliations:** 1 Manaaki Whenua Landcare Research, Private Bag 92170, Auckland, 1142 New Zealand; 2 Department of Organismic and Evolutionary Biology, Harvard Herbarium, 22 Divinity Ave, Cambridge, MA 02138 USA; 3 Blaihofstr aße 4272074, Tübingen, Germany; 4 Department of Botany, National Museum of Nature and Science, 4-1-1 Amakubo, Tsukuba, Ibaraki 305-0005 Japan; 5 Leibniz-Institute DSMZ German Collection of Microorganisms and Cell Cultures, Inhoffenstrasse 7B, 38124 Braunschweig, Germany; 6 Institute of Ecology and Earth Sciences, University of Tartu, Lai 40, EE-51005 Tartu, Estonia; 7 State Key Laboratory of Mycology, Institute of Microbiology, Chinese Academy of Sciences, Beijing, 100101 China; 8 Faculty of Science, University of South Bohemia, Branišovská 31, 370 05 České Budějovice, Czech Republic; 9 Yale Centre for Genome Analysis, Yale University, Orange, CT 06477 USA; 10 Department of Biostatistics, Yale University, 135 College St, New Haven, CT 06510 USA

**Keywords:** *Helotiales*, *Marthamycetales*, *Leotiales*, *Thelebolales*, *Phacidiales*, *Rhytismatales*, *Chaetomellales*, *Erysiphales*, Genome phylogeny, Three new taxa

## Abstract

Fungi in the class *Leotiomycetes* are ecologically diverse, including mycorrhizas, endophytes of roots and leaves, plant pathogens, aquatic and aero-aquatic hyphomycetes, mammalian pathogens, and saprobes. These fungi are commonly detected in cultures from diseased tissue and from environmental DNA extracts. The identification of specimens from such character-poor samples increasingly relies on DNA sequencing. However, the current classification of *Leotiomycetes* is still largely based on morphologically defined taxa, especially at higher taxonomic levels. Consequently, the formal *Leotiomycetes* classification is frequently poorly congruent with the relationships suggested by DNA sequencing studies. Previous class-wide phylogenies of *Leotiomycetes* have been based on ribosomal DNA markers, with most of the published multi-gene studies being focussed on particular genera or families. In this paper we collate data available from specimens representing both sexual and asexual morphs from across the genetic breadth of the class, with a focus on generic type species, to present a phylogeny based on up to 15 concatenated genes across 279 specimens. Included in the dataset are genes that were extracted from 72 of the genomes available for the class, including 10 new genomes released with this study. To test the statistical support for the deepest branches in the phylogeny, an additional phylogeny based on 3156 genes from 51 selected genomes is also presented. To fill some of the taxonomic gaps in the 15-gene phylogeny, we further present an ITS gene tree, particularly targeting ex-type specimens of generic type species. A small number of novel taxa are proposed: *Marthamycetales* ord. nov., and *Drepanopezizaceae* and *Mniaeciaceae* fams. nov. The formal taxonomic changes are limited in part because of the ad hoc nature of taxon and specimen selection, based purely on the availability of data. The phylogeny constitutes a framework for enabling future taxonomically targeted studies using deliberate specimen selection. Such studies will ideally include designation of epitypes for the type species of those genera for which DNA is not able to be extracted from the original type specimen, and consideration of morphological characters whenever genetically defined clades are recognized as formal taxa within a classification.

**Electronic supplementary material:**

The online version of this article (10.1186/s43008-019-0002-x) contains supplementary material, which is available to authorized users.

## INTRODUCTION

The class *Leotiomycetes* was erected when the superclass *Leotiomyceta* was split into seven classes by Eriksson and Winka (1997). It is recognized as one of the most diverse classes in the subphylum *Pezizomycotina*, which in turn is one of the most diverse groups within *Ascomycota* (Berbee 2001). No recent estimates of the diversity of the entire *Leotiomycetes* have been made, but Hawksworth (2001) provided estimates for *Erysiphales* (10,000 spp.), a moderately well-studied order of *Leotiomycetes*, and *Helotiales* (70,000 spp.), a poorly studied order. Using these numbers as rough guides, we can expect a minimum of 80,000 species for the class. Current estimates put the number of published species in the class in the range 4407–5587, meaning that at most about 5–7% of the species diversity is currently known (Kirk et al. 2008; Baral 2016; Wijayawardene et al. 2017, 2018).

The class comprises non-lichenized ascomycetes, with those species that form a sexual morph historically referred to as “inoperculate discomycetes”. They are characterized by the production of an open ascoma (apothecium) and unitunicate asci generally opening by a pore (Eriksson 2005). In the last decade, this concept has changed mostly due to the contributions of molecular analyses in the systematics of the class. As currently conceived, the class is morphologically more diverse than Eriksson’s concept, including taxa with conspicuous or inconspicuous fruitbodies, usually apothecial, some opening late to expose the hymenium (pro- or mesohymenial phase) at maturity (e.g. *Marthamycetaceae*, *Phacidiales, Rhytismatales*), immersed in stromatic tissues or not, or with cleistothecial ascomata (e.g. *Erysiphales*). Some fungi now known to be members of *Leotiomycetes* have no known, or a rarely detected, sexual morph. These include vascular pathogens such as *Cadophora*, *Phialocephala* and *Collophorina* (Gams 2000; Damm et al. 2010; Day et al. 2012), the aquatic hyphomycetes (Baschien et al. 2013), mycorrhizal species, and root-inhabiting dark septate endophytes (Grünig et al. 2011).

The current classification of the class includes 11 orders, ca. 43 families, ca. 592 genera and ca. 4407 species, but these numbers vary depending on the taxonomic concepts applied by different authors, for example Baral (2016) versus Wijayawardene et al. (2017, 2018). *Leotiomycetes* also feature a remarkably high number of monotypic taxa: seven orders have only one family, two orders have only one species, 11 families have only one genus and ca. 231 genera have only one species (Baral 2016; Wijayawardene et al. 2017, 2018; Quijada 2018). The diversity is not equal across the class, *Helotiales* (ca. 2334 species) contains half of the described species, with three to five times more species than the second or the third most diverse orders, *Erysiphales* (ca. 747 spp.), and *Rhytismatales* (ca. 497 spp.). *Helotiales* is also the order in *Leotiomycetes* with the most genera placed as *incertae sedis* at the familial rank (ca. 90–151; Baral 2016, Wijayawardene et al. 2017, 2018, Quijada 2018). Currently, *Helotiales* includes 26 families, within which about 19–27% of the genera have an uncertain position at the family level (Baral 2016). Although the latest classifications of *Leotiomycetes* (*op. cit.*) have drastically changed the number of families and the placement of genera compared to earlier classifications (e.g. Lumbsch and Huhndorf 2010), the general concept of the order has remained the same for several decades and is now failing to reflect increasingly well-understood phylogenetic relationships.

An early class-wide phylogenetic analysis of *Leotiomycetes*, published as part of the AFTOL-1 project, was based on just the SSU, LSU and 5.8S rDNA regions (Wang et al. 2007). That study confirmed the placement of *Geoglossaceae* outside *Leotiomycetes*, the inclusion of *Erysiphales* and *Rhytismatales* in the class, and noted the polyphyly of *Helotiales* (Wang et al. 2007). Since then, other large phylogenies treating taxa within this class include Stenroos et al. (2010), Lantz et al. (2011), Han et al. (2014), Crous et al. (2014), Baral et al. (2015), and Pärtel et al. (2017). Lantz et al. (2011) demonstrated that some genera traditionally placed within *Rhytismatales* were related to other taxa placed in the polyphyletic *Helotiales*. Han et al. (2014) pointed out the extreme polyphyly of the family *Hyaloscyphaceae*, which constitutes at least 10 different clades within *Leotiomycetes*. The same year, Crous et al. (2014) circumscribed *Phacidiaceae*, previously considered a family in *Helotiales*, and accepted the order *Phacidiales* as a sister clade to *Helotiales*. A year later, Baral et al. (2016) suggested a new classification of *Leotiomycetes*. Baral (2016) provided more detail on thisnew classification, making many important changes at the family level, using both genetic and morphological evidence to establish several new families, to shift genera between families, and to revive several families that had fallen out of use. Baral (2016) accepted paraphyletic groups when strong morphological support was noted (e.g. *Geoglossaceae* was included in *Leotiales*, *Tympanidaceae* was included in *Phacidiales*). After this classification, three relevant publications appeared, Pärtel et al. (2017), Hernández-Restrepo et al. (2017), and Crous et al. (2017). In the first, evidence of the paraphyly of *Helotiales*, forming a clade that included *Erysiphales* and *Cyttariales*, was presented again, in a study that focused on the encoelioid taxa. The second established a new monotypic family and order for the genus *Lauriomyces*, and the third established two new families —*Cochlearomycetacae* and *Neocrinulaceae* —for two new, genetically isolated genera. Most of these phylogenies have a common problem, in that they are based on rDNA sequences which generally do not inform high-level relationships (Liu et al. 1999). Only four of these phylogenies included RPB2 sequences in combination with ribosomal DNA (rDNA) sequences (Stenroos et al. 2010; Han et al. 2014; Crous et al. 2014; Pärtel et al. 2017). The maximum number of gene regions used was six (Pärtel et al. 2017), and that is the only study to have used more than one protein-coding gene (EF1a, RPB1, and RPB2). Accounting for all of these phylogenies, only 32% of the genera included in *Leotiomycetes* have been incorporated at least once in a large phylogeny with more than three molecular markers (Quijada 2018). Although the families proposed by Baral (2016) are usually supported in these analyses, several of them are paraphyletic (e.g. *Helotiaceae*, *Lachnaceae*, *Pezizellaceae*, *Rutstroemiaceae*, *Tympanidaceae*), and in none of these phylogenies have the deeper, backbone relationships between families or orders been strongly supported. Several taxa remain as members of informal lineages (e.g. *Bryoglossum*, *Stamnaria*, *Strossmayeria*) sensu Baral (2016) or are placed as *incertae sedis* in Wijayawardene et al. (2017). Also, a high proportion of taxa have not yet been treated at all using molecular methods (Baral 2016; LoBuglio and Pfister 2010).

Most mycologists and users of fungal classifications, such as plant pathologists and mycorrhizal ecologists, now routinely identify their specimens using DNA sequences. Accurate morphological identifications depend on critical obseravtions and mycologists able to perform these are in rapid decline. Morphological characters can be misleading if incorrectly interpreted, being influenced by the methods used in morphological studies (Baral 1992) or the inherent morphological plasticity of many characters. Also, an increasing number of *Leotiomycetes* are known only from the character-poor asexual morph. DNA sequences provide an extremely high number of useable characters compared with the ones that we can find with morphology. Nevertheless, it is necessary to associate these sequences with reliable names because names link the sequences to historical, accumulated information on the biology, host range, distribution and pathogenic potential of the taxa detected (Crous et al. 2015). Reconciling morphology with phylogeny may not always be possible, but understanding molecular phylogenetic relationships as accurately as possible — and ideally reflecting them taxonomically in classifications — is important for understanding the role that fungi play in the ecosystems in which they have been detected. To enable this understanding, named fungarium specimens and cultures, especially type specimens, need to be linked with sequences (Truong et al. 2017).

*Leotiomycetes* has a worldwide distribution, and species have diverse ecological roles in soil, water or in the air, from the tropics to temperate, boreal or arctic-alpine, humid to arid ecosystems(O’Brien et al. 2005; Sieber 2007; Baral 2016). Taxa associated with fresh water include two families that characteristically form a sexual morph (*Mitrulaceae* and *Vibrissaceae*; Baral 2016), while many other genera from fresh-water are only known from an asexual morph (e.g. “aquatic hyphomycetes” in Raja et al. 2008, Gulis et al. 2012, Baschien et al. 2013). They have been found in many substrates in marine environments (Gnavi et al. 2014), including sponges in the Antarctic (Henríquez et al. 2014). *Bryoglossum* and *Leotia* form mycorrhizas, which have also been observed in some species of *Mollisiaceae*, *Hyaloscyphaceae*, and *Myxotrichiaceae* (Zijlstra et al. 2005; Kühdorf et al. 2015; Baral 2016). Endophytes have been found in several lineages of *Dermateaceae*, *Gelatinodiscaceae*, *Helotiaceae*, *Phacidiaceae* and *Rhytismataceae* (Baral 2016). Five families are exclusively plant pathogenic (*Cyttariaceae*, *Erysiphaceae*, *Drepanopezizaceae*, *Medeolariaceae*, and *Sclerotiniaceae*), one typically includes fungal parasites (*Helicogonaceae*), and some reportedly exhibit strong host specificity: the *Stamnaria* lineage (on *Equisetum*) and the *Mniaecia* lineage (on liverworts) (Baral 2016). Some members of the class are pathogenic to mammals, such as *Pseudogymnascus destructans,* which causes white-nose syndrome of bats (Blehert 2012). Despite this great diversity, the two predominant life-styles are saprobic and parasitic, with both strategies coexisting in the majority of families included in the class. Based on current sampling, the saprobic lifestyle appears to be the most common ecology in the class, more than half of the families having saprobic members growing on dead or decaying plant material (Baral 2016).

DNA-barcoding and high-throughput sequencing methods have changed the way fungal diversity in ecosystems can be measured. These new techniques allow the assessment of the fungal diversity in the environment that is independent of direct observation of fungal structures or cultivation of the fungi (Mitchell and Zuccaro 2006; Stewart 2012; Thomsen and Willerslev 2015). Purely molecular sampling methods have led to an increased number of sequences in general repositories, some identified but many unidentified, inspiring a debate as to whether sequences or eDNA should be allowed to serve as name-bearing types to describe and catalogue this hidden diversity (Hibbett et al. 2016; Hawksworth et al. 2018; Zamora et al. 2018; Hongsanan et al. 2018). The class *Leotiomycetes* provides a good example of how these techniques are revealing diversity, as members have been commonly found in permanently frozen soil (Lydolph et al. 2005), associated with roots (Hambleton and Sigler 2005; Toju et al. 2013; Hazard et al. 2014; Koizumi and Nara 2017), associated with bryophytes (Kauserud et al. 2008), on soil (Varma and Oelmüller 2007), in seawater (Henríquez et al. 2014), in air samples (Banchi et al. 2018), and elsewhere. Unfortunately, it is often impossible to associate these eDNA sequences with known species. In part this mismatch occurs because many taxa remain undiscovered and many named taxa lack DNA sequence data. Nevertheless, a classification of *Leotiomycetes* that better reflects molecular phylogeny would enable even the unknown taxa to be placed more reliably in a taxonomic framework, and through this placement their role in the ecosystems where they are detected can be better predicted (Wang et al. 2011).

In summary, the taxonomy and classification of *Leotiomycetes* remains unsettled. The current classification is still largely based on taxa defined by morphology and there is a disconnect between this morphology-based classification and molecular phylogenies. Because of its ecological diversity, the class has been studied by several distinct research communities, and this has resulted in the adoption of different classifications with different opinions about higher classification schemes. This problem needs to be solved, and the alternative schemes reconciled to develop one common classification. During the 11th International Mycological Congress in Puerto Rico (IMC11) in July 2018, a workshop on *Leotiomycetes* taxonomy was held, where 35 researchers were surveyed for their opinions on the current problems in the class: 80% of them agreed that the lack of specialist, general treatments, keys or sequences for common genera or species was a central issue; and 100% agreed that the main problem is polyphyly at different taxonomic levels (Quijada 2018). Here we use available data from published, taxonomically narrowly focused multi-gene studies, data extracted from published genomes, and some novel genome and sequence data, to provide a more robust class-wide phylogeny for *Leotiomycetes*. The aim is to provide a basis for taxon and specimen selection for future studies attempting to resolve outstanding issues associated with particular clades or subclades supported in the analyses presented here.

## METHODS

Three sets of phylogenetic analyses were undertaken, one based on several thousand genes that were selected from 51 genomes, including the 10 new *Leotiomycetes* genomes released here (*see below*); a second based on up to 15 genes for a set of specimens selected to represent as widely as possible the genetic diversity across the class *Leotiomycetes* as understood from current rDNA-based class-wide phylogenies (e.g. Wang et al. 2007) and to include taxa known from both asexual and sexual morphs (e.g. Baschien et al. 2013); and a third, based on ITS sequences alone, which includes many taxa that were not represented in the other more data-rich analyses. Whenever possible, specimens were selected that represent the type species of a genus and where possible the type specimens themselves were used. If the only generic name available for a species is considered to be incorrect, it is cited in double quotes.

A complete list of the specimens and genomes sampled is given in Additional file 1: Table S1 and Additional file 2: Table S2.

### Gene selection

The genes targeted for the 15-gene analysis were the ribosomal genes (SSU, 5.8S, LSU), and genes commonly used in recent multi-gene studies that include *Leotiomycetes* — β-tubulin, EF1a, MCM7, mtSSU, RPB1 and RPB2 (e.g. Baral et al. 2009; Chen et al. 2016; Crous et al. 2014; Han et al. 2014; Hosoya et al. 2010, 2011; Hustad and Miller 2011; Iturriaga et al. 2017; Lorch et al. 2013; Malloch et al. 2016; Pärtel et al. 2017; Sanoamuang et al. 2013; Schoch et al. 2009; Spatafora et al. 2006; Zhao et al. 2012).

Several additional genes were selected on the basis of a phylogenetic informativeness analysis using PhyDesign (Lόpez-Giráldez and Townsend 2011) to determine genes likely to be highly informative at deep levels within the phylogeny. An ultrametric tree was calculated for the taxa sampled in the single-copy orthologue dataset of Rokas et al. (2005) as modified by Taylor and Berbee (2006), using the methods of Taylor and Berbee (2006). Calibration points targeted divergence of major *Pezizomycotina* lineages, approximately 250–400 million years ago, based on the fungal, animal and plant calibrations of Taylor and Berbee (2006) (Additional file 3: Figure S1). Phylogenetic informativeness profiles were then calculated for gene fragments within the gene-rich taxon-poor sequence dataset of Taylor and Berbee (2006), and within the taxon-rich gene-poor dataset from the AFTOL-1 project (Schoch et al. 2009). Further consideration was dedicated to the 10 most informative genes for a timescale approximating early divergence within *Leotiomycetes*. These genes are listed, with their phylogenetic informativeness, along with potential primers for PCR amplification in Additional file 4: Table S3. Of these genes, RPB1 was already being targeted based on published multi-gene studies of the class. Preliminary attempts to amplify the other putatively highly informative genes were not consistently successful. However, several of these genes, RPA1, RPA2, RPC2, SF3B1, TFB4 and α-tubulin, were readily detected in the *Leotiomycetes* genomes we sampled (Additional file 1: Table S1). For those specimens that had genomes available, these genes were incorporated into the 15-gene analysis.

### Newly generated DNA sequences

Newly generated Sanger sequences for SSU, ITS, LSU, β-tubulin, RPB1, RPB2, mtSSU, EF1a, and MCM7 were sourced from several laboratories, variously from cultures grown from ascospores discharged from apothecia, or from conidia from fresh water, cultures isolated from diseased plant tissue, and whole (single or multiple) apothecia. Sequences have been deposited in GenBank, or those that are unpublished or have been extracted from genomes, are available in the alignments provided in the Manaaki Whenua Datastore, 10.7931/T5YV-BE95. The voucher specimens from which the sequences were derived, GenBank accession numbers, and the researchers who generated the sequences are listed in Additional file 1: Table S1.

### Genome sequencing and assembly

Genomic DNA for genome sequencing was isolated using the method of Schwessinger and McDonald (2017) and further purified using a QIAGEN Genomic-tip. Genomic DNAs were quantified using a fluorometer, and purity and integrity were assessed with a spectrophotometer and by agarose-gel electrophoresis. Sequencing was performed by Macrogen (South Korea) with an Illumina HiSeq2500 using a TruSeq Nano DNA kit with 100 bp paired-end reads. Multiplexing with 10 samples per lane resulted in 4.9–6.3 Gb per sample, with Q30 Phred quality score (99.9% base call accuracy) of 88–91%.

Short-read illumina sequence data was assembled using two different genome assembly tools: Platanus v. 1.2.4 (http://platanus.bio.titech.ac.jp/platanus-assembler/platanus-1-2-4), and the A5 Miseq Pipeline (Coil et al. 2015). For assembly with Platanus, sequence data was pre-processed using platanus_trim (http://platanus.bio.titech.ac.jp/pltanus_trim) followed by contig assembly, scaffolding, and gap closure using Platanus. The A5 Miseq pipeline used Trimmomatic (Bolger et al. 2014) for removal of adapter sequences and low-quality regions, followed by contig assembly using the IDBA-UD algorithm (Peng et al. 2012). Initial scaffolding, misassembly correction and final scaffolding was undertaken using SSPACE (Boetzer et al. 2010), bowtie (Langmead et al. 2009), samtools (Li et al. 2009), and BWA (Li and Durban 2009). QUAST (Gurevich et al. 2013) and BUSCO v3.0.2 (Simão et al. 2015; Waterhouse et al. 2017) were used to assess genome assembly completeness and quality. QUAST was run using the –eukaryote flag. BUSCO was run in genome mode with the *Pezizomycotina* odb9 database, of which *Aspergillus nidulans* is the default species.

### Phylogenetic analyses

For the phylogeny based on 51 genomes, each genome was analyzed with BUSCO v3.0.2 using the *Pezizomycotina* odb9 database. This database contains 3156 putative single-copy reference genes which can be searched for within submitted genomes. The orthologous protein sequences identified using BUSCO were used for a phylogenetic analysis. Duplicate genes were removed from the phylogenetic analysis for those taxa where they were detected. Orthologous amino acid sequences were aligned separately using MUSCLE v. 3.8.31 (Edgar 2004). Following this alignment, gaps and phylogenetically uninformative positions were removed using Gblocks v. 0.91b (Castresana 2000; Talavera and Castresana 2007) with default settings. All aligned genes were concatenated into a supermatrix using Geneious v. 10 (Kearse et al. 2012), with sites of missing genes represented by N characters. A Bayesian Inference phylogeny was estimated using ExaBayes v. 1.5 (Aberer et al. 2014). Two independent runs were undertaken; following the recommended guidelines, analysis stopped after each had run for > 100,000 generations and the combined ESS values were > 100. *Xylaria hypoxylon* (JGI genome Xlyhyp1) and *Neurospora crassa* (JGI genome Neucr2) were used as outgroups.

For the phylogeny based on 15 genes, only 30 of the 279 specimens treated have all 15 genes available, but more than half have five or more genes (Additional file 5: Table S7). The sequences available for each gene were aligned using MAFFT (Katoh and Standley 2013) as implemented in Geneious 10. The ends were manually trimmed and introns were removed manually; all remaining data were then concatenated. Maximum likelihood analyses were run with IQ-TREE (Nguyen et al. 2015; Chernomor et al. 2016), using models selected by ModelFinder (Kalyaanamoorthy et al. 2017) for each partitioned gene; ultrafast bootstrap (BS) analysis with 1000 replicates estimated branch support in the ML tree (Hoang et al. 2018). *Xylaria hypoxylon* and *Neurospora crassa* were used as outgroups. The taxa and genes included in the alignment are provided in Additional file 1: Table S1 and the models used for each partition are in the Manaaki Whenua Landcare Research datastore, 10.7931/T5YV-BE95.

The ITS phylogeny includes 568 sequences. The methods for the analysis match those used for the 15-gene concatenated tree, but using the TIM2 + F + R9 model. Details of the taxa, specimens and GenBank sequence accessions are provided in Additional file 2: Table S2.

## RESULTS

**Fig. 1:**
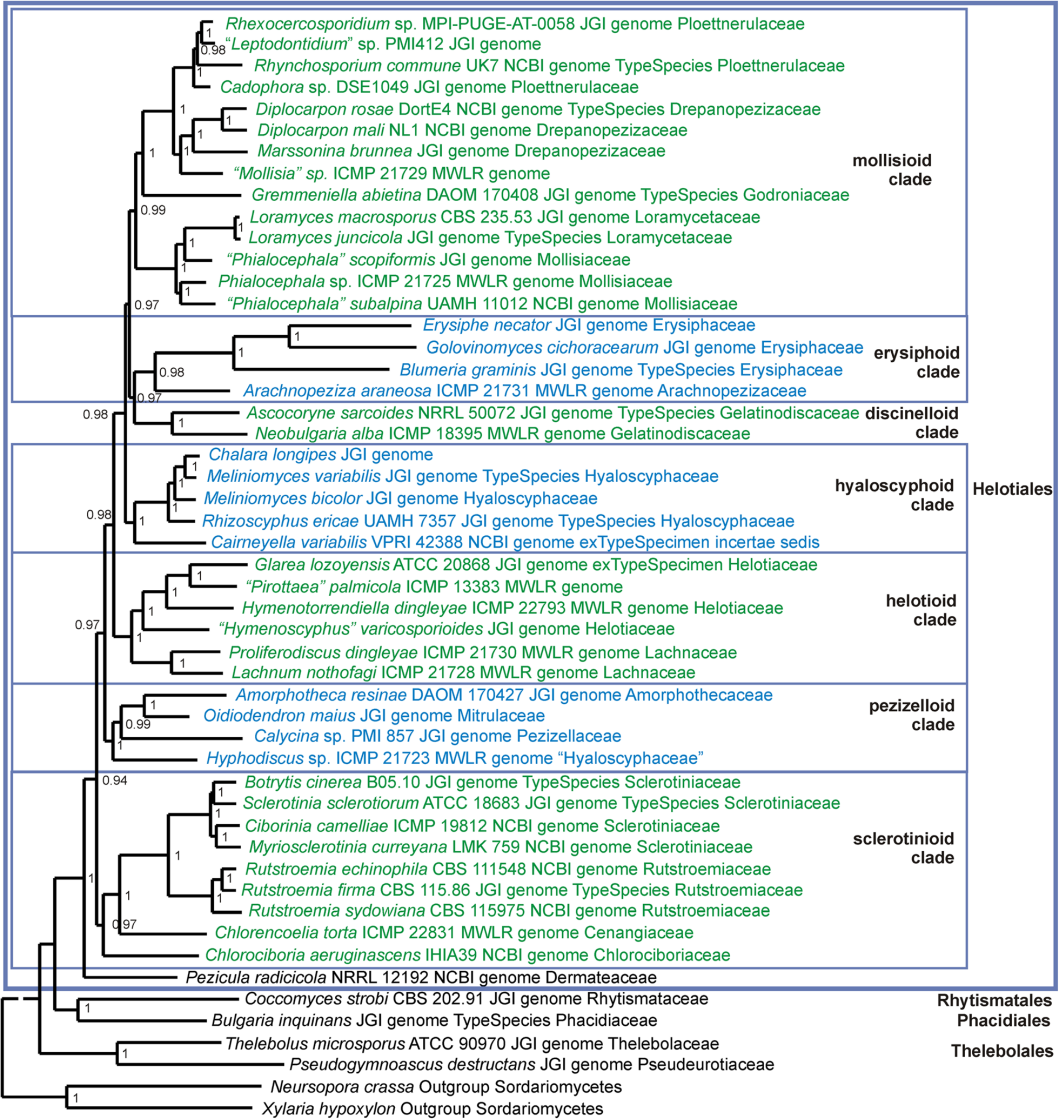
Phylogenetic tree based on a Bayesian analysis of 3156 concatenated orthologous single copy genes recognized using BUSCO from 49 selected *Leotiomycetes* genomes plus two outgroup genomes (*Xylaria hypoxyolon* and *Neurospora crassa*). Labels for the specimens sampled include the taxonomic name, voucher specimen where this data is available, source of the genome, and family level classification for that genus as accepted in Baral (2016). The large *Helotiales* clade is divided into several informal subclades that are discussed in the text

**Fig. 2:**
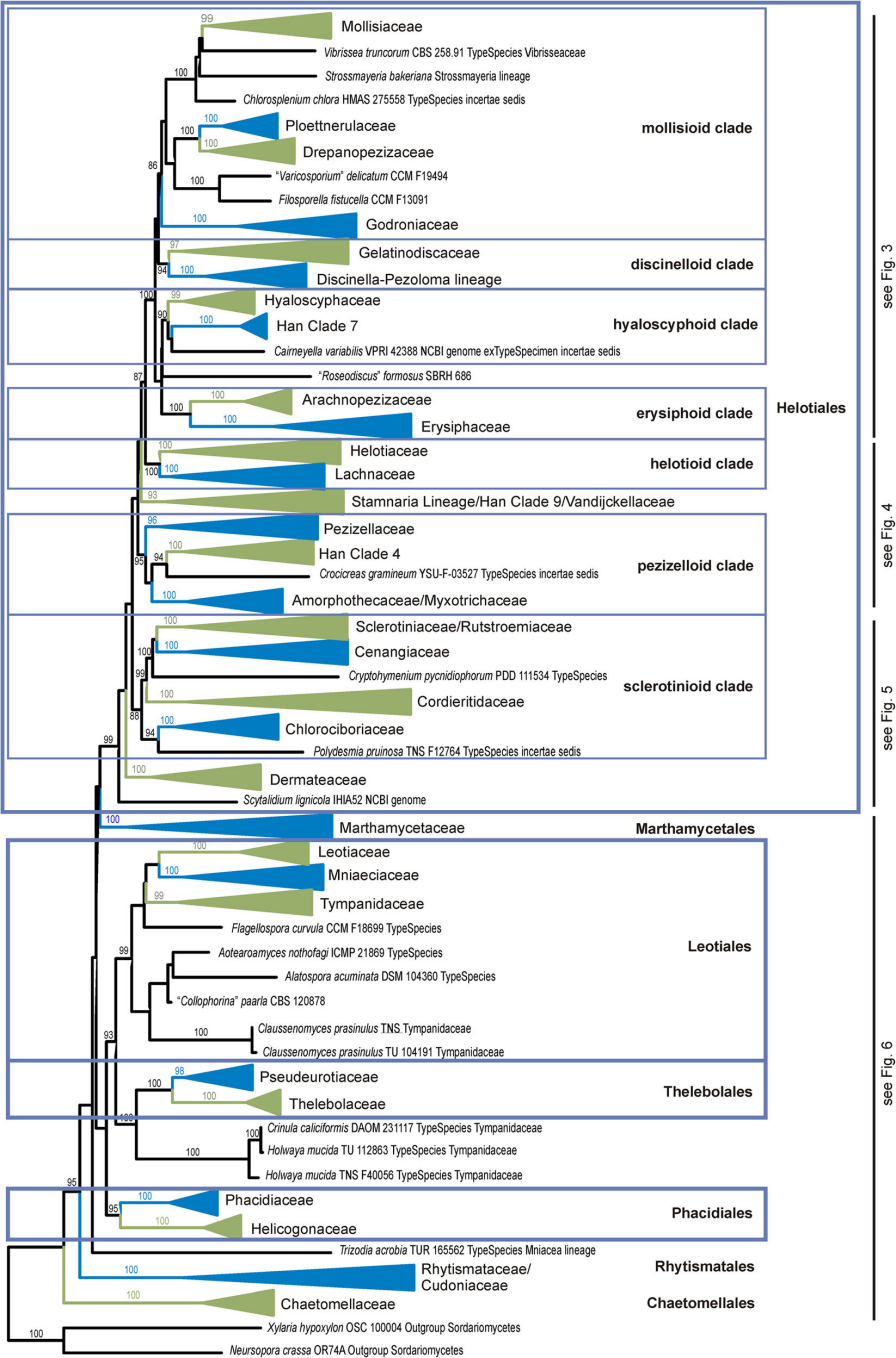
Summary tree from the ML analysis based on 15 concatenated sequences. Names for the collapsed family level clades are those accepted in this paper and generally the clades follow Baral (2016), the clades labelled Han 4, 7, 9 are those recognized in Han et al. (2014). *Helotiales* sensu Baral (2016) is divided into several informal clades based on bootstrap support in both this tree and Bayesian PP support in the genome-scale phylogeny (Fig. 1). The labels for taxa which are not included in one of the family-level clades include the voucher specimen from which the sequences were obtained, the type status of the specimen (whether it is the ex-type specimen of the type species for that genus, or whether it has been identified as the type species for that genus), the family in which it was placed by Baral (2016), if it was treated in that work, and the source of the genome data for those that have had their genome sequenced. Bootstrap values 90% or greater indicated, in a few cases lower bootstrap values for a few key branches are also indicated. Figures 3, 4, 5 and 6 provide detail of species included in each clade

**Fig. 3:**
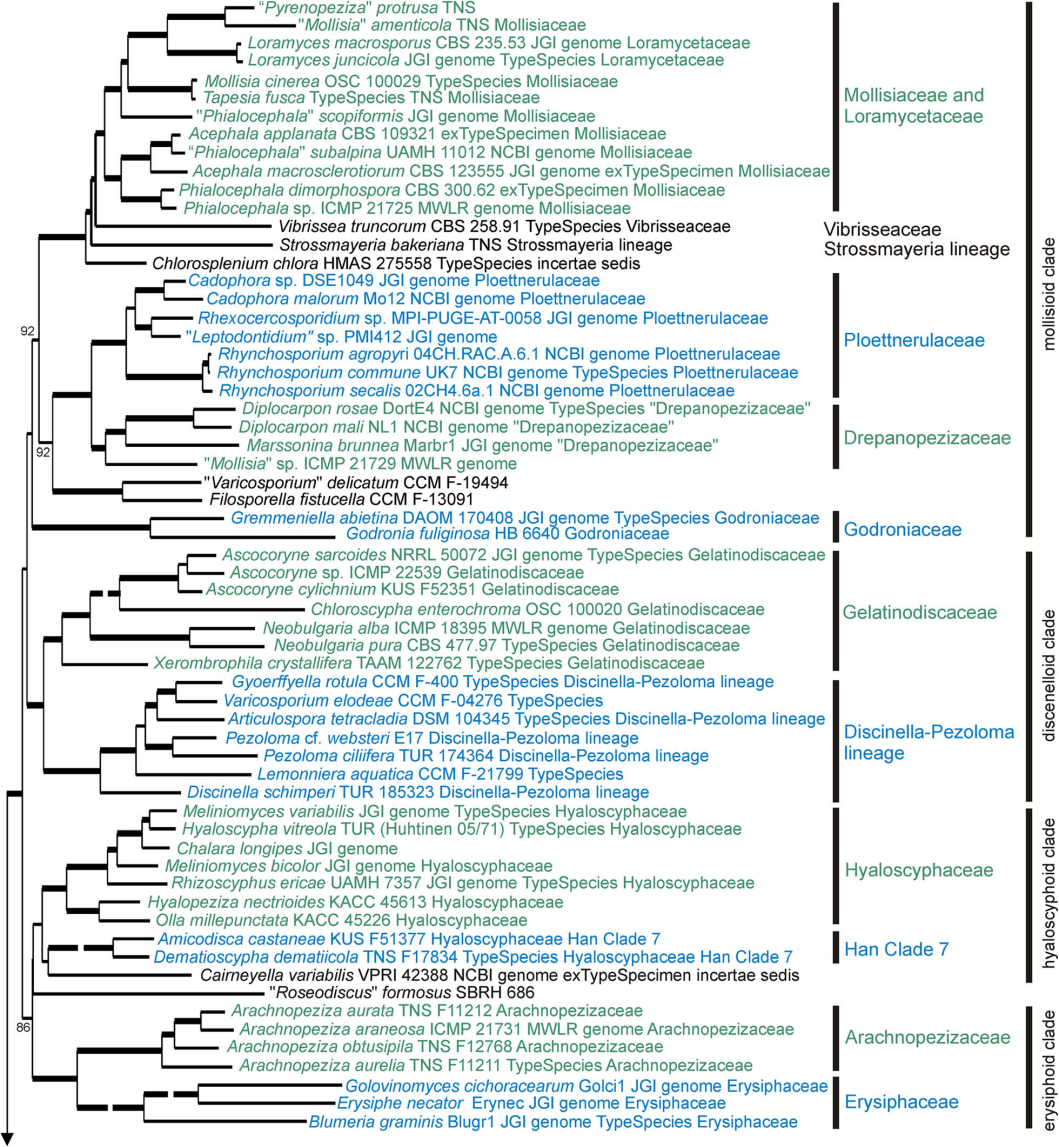
Detail from Fig. 2. ML tree based on 15 concatenated sequences for specimens within the informal “mollisioid clade”, “discinelloid clade”, “hyaloscypheloid clade” and “erysiphoid clade” within *Helotiales*. Family level taxa indicated to the right are those accepted in this paper, ‘Han Clade 7’ is from Han et al. (2014). Thick branches have bootstrap values > 95%, lower bootstrap values for a few key branches are also indicated. The labels for taxa include the voucher specimen from which the sequences were obtained, the type status of the specimen (whether it is the ex-type specimen of the type species for that genus, or whether it has been identified as the type species for that genus), the family in which it was placed by Baral (2016), if it was treated in that work, and the source of the genome data for those that have had their genome sequenced. Some very long branches have been shortened for presentation; the Figs. 2, 3, 4, 5 and 6 nexus file in 10.7931/T5YV-BE95 has the original scale

**Fig. 4:**
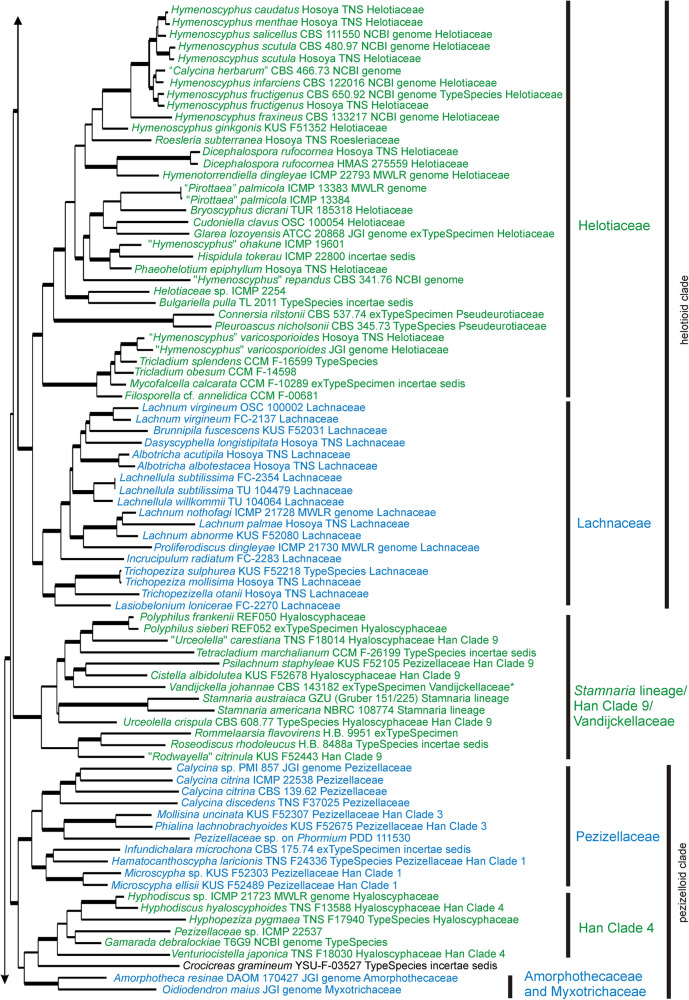
Detail from Fig. 2. ML tree based on 15 concatenated sequences for specimens within the informal “helotioid clade” and “pezizelloid clade”, and the *Stamanaria* lineage within *Helotiales*. Family level taxa indicated to the right are those accepted in this paper, ‘Han Clade 4’ and ‘Han Clade 9’ are from Han et al. (2014). Thick branches have bootstrap values > 95%, lower bootstrap values for a few key branches are also indicated. The labels for taxa include the voucher specimen from which the sequences were obtained, the type status of the specimen (whether it is the ex-type specimen of the type species for that genus, or whether it has been identified as the type species for that genus), the family in which it was placed by Baral (2016), if it was treated in that work (*Vandijckellaceae was described in 2017), and the source of the genome data for those that have had their genome sequenced

**Fig. 5:**
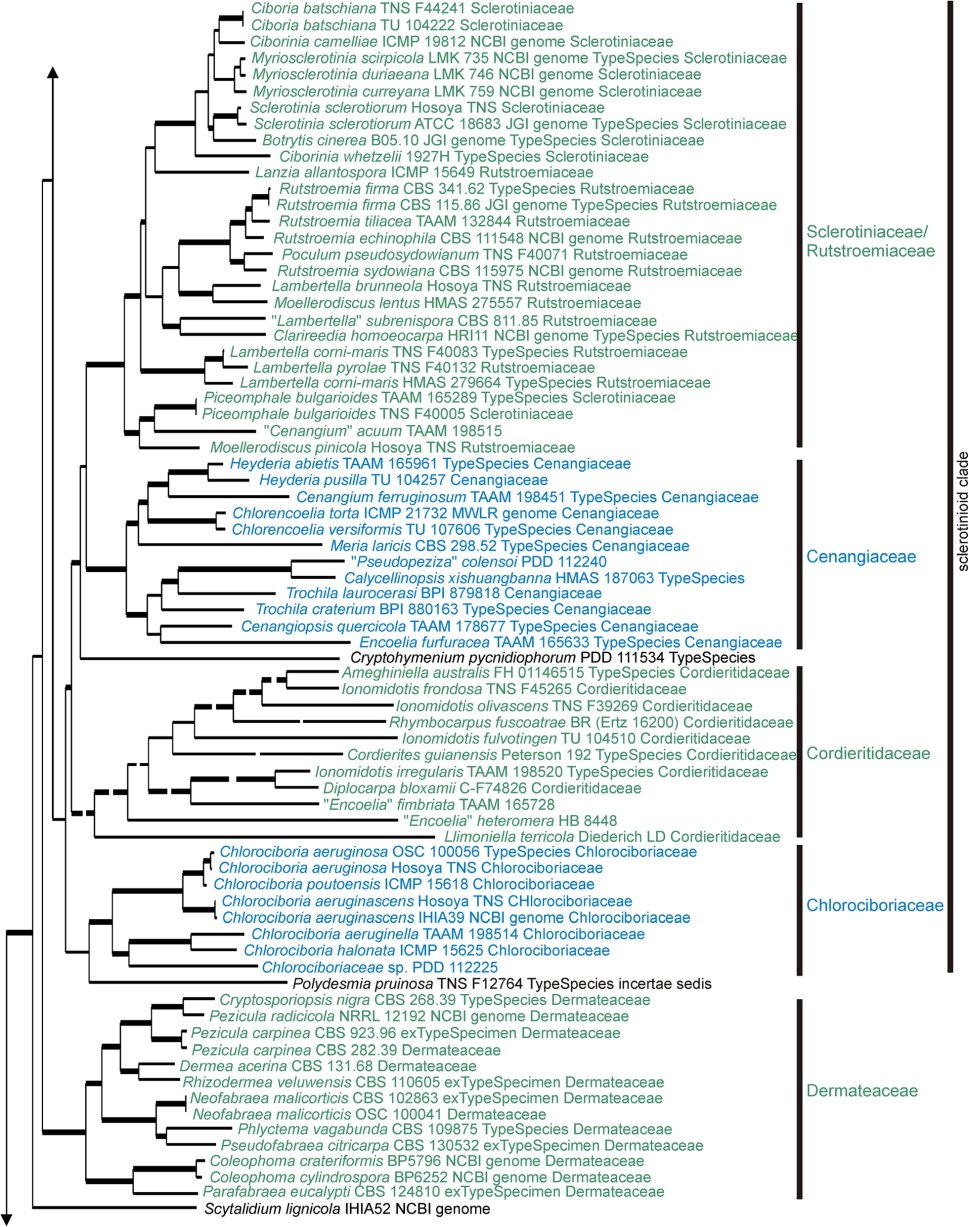
Detail from Fig. 2. ML tree based on 15 concatenated sequences for specimens within the informal “sclerotinioid clade” and *Dermateaceae* within *Helotiales*. Family level taxa indicated to the right are those accepted in this paper. Thick branches have bootstrap values > 95%, lower bootstrap values for a few key branches are also indicated. The labels for taxa include the voucher specimen from which the sequences were obtained, the type status of the specimen (whether it is the ex-type specimen of the type species for that genus, or whether it has been identified as the type species for that genus), and the family in which it was placed by Baral (2016), if it was treated in that work, and the source of the genome data for those that have had their genome sequenced. Some very long branches have been shortened for presentation; the Figs. 2, 3, 4, 5 and 6 nexus file in 10.7931/T5YV-BE95 has the original scale

**Fig. 6:**
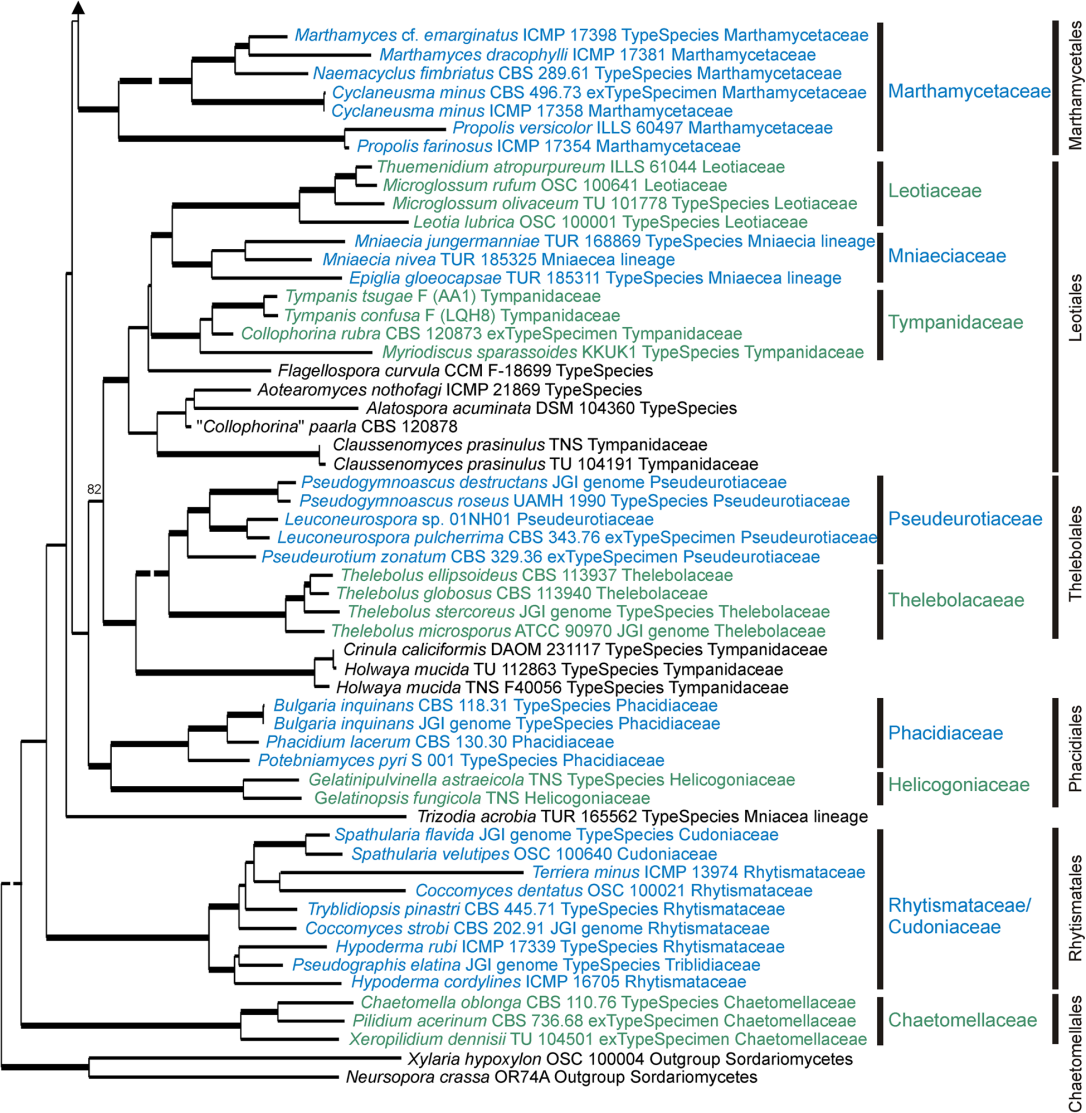
ML Detail from Fig. 2. tree based on 15 concatenated sequences for specimens within *Marthamycetales*, *Leotiales*, *Thelebolales*, *Phacidiales*, *Rhytismatales* and *Chaetomellales*. Family and order level taxa indicated to the right are those accepted in this paper. Thick branches have bootstrap values > 95%, lower bootstrap values for a few key branches are also indicated. The labels for taxa include the voucher specimen from which the sequences were obtained, the type status of the specimen (whether it is the ex-type specimen of the type species for that genus, or whether it has been identified as the type species for that genus), the family in which it was placed by Baral (2016), if it was treated in that work, and the source of the genome data for those that have had their genome sequenced. Some very long branches have been shortened for presentation; the Figs. 2, 3, 4, 5 and 6 nexus file in 10.7931/T5YV-BE95 has the original scale

Results from the three phylogenetic analyses are provided as phylogenetic trees, one based on single copy genes across 51 genomes (Fig. 1), another based on up to 15 genes for 279 specimens across 68 genera (Figs. 2, 3, 4, 5 and 6), and a third using 568 ITS sequences (Additional file 6: Figure S2) representing 315 genera (including 80 generic type specimens). A comparison of the current family-level taxonomy with the taxonomic relationships suggested by our three sets of analyses is provided in Additional file 7: Table S6. The Supplementary Data, along with Nexus files of the 15-gene tree and the ITS tree, and the alignments on which they are based, can be downloaded from the Manaaki Whenua – Landcare Research datastore, see 10.7931/T5YV-BE95.

### Genome assembly and phylogeny

The 10 draft nuclear genomes have been deposited as NCBI Bioproject PRJNA487672. Data on genome size, coverage, GC content, number and size of contigs is provided in Additional file 8: Table S4. The A5 Miseq assembly pipeline generally provided greater coverage, higher N_50_ values, and a greater number of unique predicted genes compared with the Platanus assembler. Based on a BUSCO comparison, all but two of our genomes assembled using the A5 Miseq assembler provided a completeness greater than 98%; the other two provided 96.8 and 90.1% completeness (Additional file 9: Table S5).

The 10 newly generated genomes plus a further 41 genomes were selected for phylogenetic analysis. From all but one of these genomes, we were able to detect more than 80% of the 3156 genes in the BUSCO *Pezizomycotina* database, with sets of genes for most genomes being more than 98% complete (Additional file 9: Table S5). The only poorly sampled genome was the powdery mildew *Golovinomyces cichoracearum* (54.8% completeness), although a notably large number of duplicate genes were detected for *Diplocarpon rosae*.

The genome phylogeny had posterior probability support of > 0.95 for all branches (Fig. 1). The clades resolved are congruent with the family-level classification of Baral (2016). Strong support for deeper branches suggests that it is possible to identify a set of higher-level taxa within the current order *Helotiales*. Taxon sampling depth near the base of the tree is inadequate to draw any taxonomic conclusions for those taxa included that are in *Leotiomycetes* orders other than *Helotiales*.

### 15-gene phylogeny

Based on the 15-gene phylogeny, 258 species of Leotiomycetes were distributed across 31 formal and informal family-level clades as shown in the summary tree in Fig. 2. Placement of individual taxa within the clades is detailed in Figs. 3, 4, 5 and 6. Generally, the family-level relationships resolved in Figs. 2, 3, 4, 5 and 6 are congruent with the family level classification presented in Baral (2016). Exceptions are discussed below. Based on this phylogeny, a new order and two new families are introduced, along with the validation of an existing family name; these are formally treated in the Taxonomy section below. A series of informal clades are recognized within the large order *Helotiales*. The limits for these clades are based on support in the 15-gene phylogeny, as well as in the genome-scale phylogeny (Fig. 1).

Within the “mollisioid clade” of the *Helotiales* there is strong molecular phylogenetic support for a group comprising the familes *Mollisiaceae*, *Vibrisseaceae*, *Loramycetaceae*, the *Strossmayeria* lineage of Baral (2016) and the genus *Chlorosplenium* (incertae sedis in Baral 2016) (Figs. 2 and 3). Although within this group further strong molecular phylogenetic structure is recognisable, it remains a matter of debate whether or not the whole group should be included in the oldest family *Mollisiaceae*. Scattered across the subclades within the group are the type species of genera such as *Acephala, Mollisia*, *Phialocephala* and *Vibrissea* (Fig. 3). These generic names could be useful in naming some of these clades as part of a reworking of the generic classification within this group of families.

The *Discinella-Pezoloma* lineage proposed by Baral (2016) is strongly resolved. Along with the apothecial genera *Pezoloma* and *Discinella*, the family includes several aquatic genera known only as hyphomycete morphs — *Articulospora*, *Gyoerffyella*, *Lemonniera*, and *Varicosporium* (all sampled from specimens identified as the type species) (Fig. 3). Baral (2016) placed the *Discinella*-*Pezoloma* lineage with *Gelatinodiscaceae*, *Helotiaceae* and *Roesleriaceae* in his “Lineage B” (*Helotiaceae s.lat*.). In the present study, the *Discinella*-*Pezoloma* lineage and *Gelatinodiscaceae* are strongly supported as sister clades in the informal “discinelloid clade” of *Helotiales*, distant from *Helotiaceae* (incl. *Roesleriaceae*). Taxa in the *Discinella*-*Pezoloma *lineage differ from *Gelatinodiscaceae *by their paraphyses lacking vacuolar bodies, asci mostly with hemiamyloid apical rings of the *Pezicula*-type, the consistent absence of crystals, and asexual morphs being hyphomycetous with filiform or staurosporous conidia.

The “hyaloscyphoid clade” includes *Hyaloscyphcaeae* s. str., genera recognized by Han et al. (2014) as Clade 7, and the recently described mycorrhizal genus *Cairneyella* (Figs. 2 and 3). As discussed by Han et al. (2014), genera traditionally placed in *Hyaloscyphaceae* on the basis of morphology are polyphyletic. The type species of *Hyaloscypha*, *H. vitreola*, forms a strongly supported clade with root endophytic fungi in *Meliniomyces* and *Rhizoscyphus* (these latter two genera recently synonymised with *Hyaloscypha*, Fehrer et al. 2019), along with species in genera historically placed in *Hyaloscyphaceae* based on their ascoma morphology, *Olla* and *Hyalopeziza*. Also in this clade is a specimen that has had its genome sequenced and that was identified as *Chalara longipes*. Based on a comparison of SSU sequences, this specimen could be congeneric with a specimen identified as *Chalara fusarioides* (AF203463), the type species of the genus. Other genera traditionally placed in *Hyaloscyphaceae* are discussed below in the sections on Han Clade 9/*Stamnaria* lineage and the “pezizelloid clade”.

The “erysiphoid clade” includes the sister families *Erysiphaceae* and *Arachnopezizaceae* (Figs. 2 and 3). The powdery mildew family *Erysiphaceae* is traditionally treated as a separate order *Erysiphales* but is a molecular phylogenetic member of *Helotiales* as currently circumscribed.

The “helotioid clade” includes *Helotiaceae* and *Lachnaceae* (Figs. 2 and 4). *Roesleriaceae*, retained by Baral (2016) as a distinct family because of its unusual morphology, also belongs in *Helotiaceae* based on molecular phylogeny. The *Helotiaceae* clade has strong internal molecular phylogenetic structure (Fig. 4). “*Hymenoscyphus*” *varicosporioides* (genetically distinct from the type species of *Hymenoscyphus* and reportedly with a *Varicosporium*-like asexual morph later referred to the genus *Tricladium*; Sivichai et al. 2003) forms a well-resolved clade within *Helotiaceae* that in the 15-gene tree also includes the aquatic hyphomycete genera *Mycofalcella* and *Tricladium*. Two genera with reduced, cleistothecial ascomata currently classified as *Pseudeurotiaceae* — *Connersia* and *Pleuroascus* — belong in *Helotiaceae*, again in a well-resolved subclade. The *incertae sedis* genus *Bulgariella* also belongs in this family. The type species of the New Zealand endemic genus *Chaetoscypha* was recombined by Johnston (2002) as *Pirottaea nidulans*. Baral (2016) noted that morphologically *P. nidulans* has an ascus apical ring typical of *Helotiaceae* rather than *Ploettnerulaceae*, the family he accepted for *Pirottaea*. Sequences from a morphologically similar fungus from New Zealand, *Pirottaea palmicola*, support the Baral (2016) interpretation of these New Zealand “*Pirottaea”* species as *Helotiaceae*. *Lachnaceae*, the second family in the “helotioid clade”, forms a well resolved sister clade to the *Helotiaceae*.

The Han Clade 9/*Stamnaria* lineage/*Vandijckellaceae* clade includes a set of taxa with no obvious shared morphological or ecological features. Included here are specimens in the genera *Cistella* and *Urceolella* that Han et al. (2014) placed in their Clade 9 (both *Hyaloscyphaceae* sensu Baral 2016), *Stamnaria* (*Stamnaria* lineage sensu Baral and Haelewaters 2015, Baral 2016), the ex-type specimen of *Vandijckella* (*Vandijckellaceae*, Crous et al. 2017), along with the recently described *Polyphilus* (Ashrafi et al. 2018) and the type species of *incertae sedis* genera *Tetracladium*, *Rommelaarsia*, and *Roseodiscus* (Fig. 4). Baral (2016) discussed challenges with regard to morphological identification of some of the specimens referred to these genera. A targeted morphological and multi-gene molecular phylogenetic study is needed to resolve the taxonomy of this clade.

The informal “pezizelloid clade” includes the *Pezizellaceae* sensu Baral (2016) (this family includes specimens Han et al. (2014) placed in their Clades 1, 2, and 3), the genera treated by Han et al. (2014) as Clade 4 (this includes three of the genera placed in *Hyaloscyphaceae* by Baral (2016)), and the recently described mycorrhizal genus *Gamarada* (Fig. 4). The “pezizelloid clade” also contains a specimen identified as the type of *Crocicreas*, *C. gramineum*, and species in the cleistothecial genera *Amorphotheca* (*Amorphothecaceae*) and *Oidiodendron* (*Myxotrichaceae*). Fungi in *Myxotrichaceae* share the mycorrhizal lifestyle of *Gamarada*.

The “sclerotinioid” clade contains four strongly supported clades, *Sclerotiniaceae* + *Rutstroemiaceae*, *Cenangiaceae*, *Cordieritidaceae* and *Chlorociboriaceae* (Figs. 2 and 5). Although *Sclerotiniaceae* is well resolved as monophyletic, it makes *Rutstroemiaceae* as currently accepted paraphyletic. Based on the specimens we sampled, to retain monophyly within the *Sclerotiniaceae* + *Rutstroemiaceae* clade while retaining *Sclerotiniaceae* sensu Holst-Jensen et al. (1997), would require *Rutstroemiaceae* to be split into four separate families. *Calycellinopsis*, placed in *Dermateaceae* by Zhuang (1990), later in *Helotiaceae* by Zhuang et al. (2010) and then as *incertae sedis* in Baral (2016), belongs in *Cenangiaceae*. Also in the “sclerotinioid clade”, but not clearly within any of the families within the clade, are the genera *Cryptohymenium* and *Polydesmia*.

*Leotiales* as accepted here (Figs. 2 and 6) has a different concept to that of Baral (2016). It includes *Leotiaceae*, the newly described *Mniaeciaceae*, *Tympanidaceae* s. str., along with *Claussenomyces* (placed by Baral (2016) in a broader concept of *Tympanidaceae*), the recently described *Aotearoamyces* (Quijada et al. 2018), the aquatic hyphomycete genera *Alatospora* and *Flagellospora*, and another fungus that had been incorrectly placed in *Collophorina*. *Mniaeciaceae* is formally named here as a family to represent the *Mniaecia* lineage of Baral (2016, see below). Based only on the genera we sampled, *Tympanidaceae* is restricted to *Tympanis*, *Collophorina*, and *Myriodiscus*. The only other genera included in the family by Baral (2016) that we treated were *Holwaya* (here sister to *Pseudeurotiaceae* + *Thelebolaceae* in *Thelebolales*) and *Grovesiella*, here probably in *Godroniaceae* based on the ITS tree (Additional file 6: Figure S2), but on a questionably long branch.

*Thelebolales* are placed in a separate clade in Fig. 2. An alternative treatment would be to consider *Pseudeurotiaceae* and *Thelebolaceae* as morphologically highly specialised members of a genetically broader *Leotiales*. More intensive taxon sampling of the putative *Leotiales* genera discussed in the previous paragraph will be needed to resolve a defensible taxonomy for *Thelebolales*.

*Phacidiales* is here restricted to the *Phacidiaceae* and *Helicogoniaceae*. Within these families our analyses support the concepts of Baral (2016).

*Rhytismatales* in our analyses includes three families, *Rhytismataceae*, *Cudoniaceae*, and *Triblidiaceae*. A molecular phylogenetic relationship between *Rhytismataceae* and *Cudoniaceae* was recognized by Wang et al. (2002), and Magnes (1997) recognised *Triblidiaceae* as a member of the Rhytismatales based on a morphological study. The phylogenetic position of *Triblidiaceae* reported here is based on genes extracted from a genome from a single specimen (CBS 651.97) identified as *Pseudographis elatina*. This identification needs to be confirmed with additional sequence data from other specimens. *Cudoniaceae* forms a well resolved clade within a more inclusive *Rhytismataceae* + *Cudoniaceae* clade, but makes the remainder of *Rhytismataceae* paraphyletic. The morphologically based genera within *Rhytismataceae* have long been known to be incongruent with phylogeny (e.g. Lantz et al. 2011). The family requires a complete genus-level taxonomic revision. This revision must include resolution of the relationship between the families *Rhytismataceae* and *Cudoniaceae*.

Genera with enclosed ascomata have evolved independently several times across *Leotiomycetes*, but based on their distinctive ascomatal morphology they have often been treated as taxonomically distinct, despite being closely related in molecular phylogenetic studies to taxa with the disc-shaped ascomata more typical of *Leotiomycetes*. For example, the genus *Loramyces* is nested within *Mollisiaceae*, but retained as a separate family *Loramycetaceae* by Baral (2016) because of its unusual morphology, having enclosed ascomata with a perithecoid macromorphology. Other examples include *Erysiphaceae* with ascospores formed in small, enclosed, globose chasmothecia. They are generally treated as a separate order *Erysiphales*, despite being genetically members of *Helotiales* (Figs. 1 and 3). *Connersia* and *Pleuroascus*, with enclosed, cleistothecial ascomata have been placed in *Pseudeurotiaceae*, however genetically they appear to be morphologically specialised members of *Helotiaceae*. *Bicornispora*, originally placed in *Coryneliales*, is another cleistothecial member of *Leotiomycetes*, being closely related to the type species of *Lambertella* (*Rutstroemiaceae*), *L. corni-maris* (Additional file 6: Figure S2, Galán et al. (2015)). There is also strong molecular support for a sister relationship between the *Thelebolales* clade (sets of species in *Pseudeurotiaceae* and *Thelebolaceae* that have both apothecioid reduced cleistothecial ascomata) and the apothecial genus *Holwaya* and its asexual morph *Crinula*.

### ITS gene tree

The ITS gene tree (Additional file 6: Figure S2) includes many more taxa than the 15-gene tree. Although deeper relationships are poorly supported in the ITS tree, many of the family level clades that were well supported in the 15-gene tree (Fig. 2) are also resolved in the ITS tree. These clades can help inform the taxonomic relationship of some genera that are not otherwise treated. Genera that were not treated in the 15-gene phylogeny but that are placed in the ITS tree with some confidence in one of the families or clades used in Fig. 2, and that had previously been referred to a different family (Additional file 7: Table S6), are discussed below.

The families *Loramycetaceae*, *Mollisiaceae*, *Vibrisseaceae* form with *Strossmayeria* and *Chlorosplenium* a well resolved clade in the ITS tree. Genera within this clade that were not treated in the 15-gene phylogeny and not previously placed in *Mollisiaceae* include *Barrenia* and *Cheirospora* (both previously *incertae sedis*), and *Fuscosclera* (previously *Dermateaceae*).

*Ploettnerulaceae* forms a well-resolved clade in the ITS tree. In the 15-gene tree it was represented only by the important plant pathogens *Cadophora*, *Rhexocercosporidium*, and *Rhycosporium*. Based on the ITS tree other genera in this family include the aquatic *Collembolispora* and the grass parasite *Mastigosporium* (both previously *incertae sedis*).

*Godroniaceae* forms a well-resolved clade in the ITS tree and this clade includes *Grovesiella*, previously referred to *Tympanidaceae*.

*Gelatinodiscaceae* splits into two clades in the ITS tree, one of these also includes *Clathrosporium* and *Helicodendron* (previously *incertae sedis*), and the type specimen of *Dimorphospora* (previously *Helotiaceae*). *Neocudoniella*, placed in *Gelatinodiscaceae* by Baral (2016), is in the *Bryoglossum* lineage in the ITS tree.

The *Discinella*-*Pezoloma* lineage forms a well-resolved clade in the ITS tree and this clade includes several additional aquatic hyphomycete genera —*Cladochasiella*, *Fontanospora*, *Margaritispora,* and *Tetrachaetum* — as well as specimens identified as the type species of *Pseudopezicula* (tentatively placed in the *Drepanopezizaceae* by Baral 2016) and *Naevala* (previously *Calloriaceae*).

*Hyaloscyphaceae* sensu our 15-gene analysis splits into two clades in the ITS tree. *Hyaloscypha* (including *Meliniomyces*, *Pseudaegerita* and *Rhizoscyphus*) forms a clade distant to that containing *Hyalopeziza* and *Olla*. Also in the *Hyalopeziza* ITS clade is a species of *Arbusculina*, but no data is available for type material of this genus. *Unguicularia*, accepted in *Hyaloscyphaceae* by Baral (2016), has an unresolved relationship in the ITS tree and awaiting additional genetic data is here accepted as *Helotiales incertae sedis*.

In an unresolved position in the 15-gene tree is a paratype specimen identified as “*Roseodiscus*” *formosus*, genetically distinct from the type species of *Roseodiscus* (Fig. 3). Based on the ITS tree, this specimen appears to belong in a well-supported clade with members of the *Bryoglossum* lineage sensu Baral (2016) that includes *Bryoclaviculus*, *Bryoglossum*, *Neocudoniella*, and “*Crocicreas*” *multicuspidatum*. Sampling of additional genes for more of these specimens may help resolve the position of the *Bryoglossum* lineage in the context of the current 15-gene analysis.

*Arachnopezizaceae* forms a well-resolved clade in the ITS tree. Sister to this clade is another with the aero-aquatic hyphomycete *Clathrosphaerina* and the phialophora-like *Psychrophila*. Support for this relationship is also indicated by LSU sequences for both genera, but not by the EF1a and β-tubulin sequences available for *Psychrophila* (unpubl. data). Two species in this clade referred to *Durella*, *D. macrospora* and *D. melanochlora*, are phylogenetically distinct from the type species of *Durella*, *D. connivens* (Baral 2016; Additional file 6: Figure S2).

*Helotiaceae* sensu our 15-gene analysis is resolved in the ITS tree. The core *Helotiaceae* clade in the ITS tree also includes *Brunaudia* (previously *Patellariaceae*), *Endoscypha* (previously *Hyaloscyphaceae*, and based on our ITS analysis possibly synonymous with *Hymenotorrendiella*), and *Mitrulinia* (previously *incertae sedis*). The “*Hymenoscyphus*” *repandus* specimen used for a genome study is closely related to the type species of *Amylocarpus*. As in the 15-gene tree, “*Hymenoscpyhus*” *varicosporoides* forms a separate subclade, and in the ITS tree this subclade includes additional aquatic hyphomycete genera *Spirosphaera* and *Halenospora*, along with *Graddonia* and *Cudoniella*, apothecial fungi reportedly associated with wet habitats (Hustad and Miller 2011).

The Han Clade 9/*Stamnaria* lineage/*Vandijckellaceae* lineage is spread across several clades in the ITS tree. Other genera that also belong in one or other of these ITS clades are *Belonioscyphella* (previously *incertae sedis*), *Leohumicola* (previously *incertae sedis*) and *Mycoarthris* (previously *Hyaloscyphaceae*). Also belong here are specimens representing three of the genera placed in *Calloriaceae* by Baral (2016), *Calloria urticae* (type species), *Duebenia compta* (type species) and *Laetinaevia carneoflavida*.

*Pezizellaceae* sensu our 15-gene analysis splits into three clades in the ITS tree. Also in one or other of these clades are *Austropezia* (previously *Arachnopezizaceae*), *Porodiplodia* (in the newly described family *Porodiplodiaceae*, Crous et al. 2018), and the previously *incertae sedis* genera *Curviclavula*, *Hyalodendriella*, *Xenopolyscytalum*, and *Zymochalara*.

Han Clade 4 from the 15-gene tree (Fig. 5) is well resolved in the ITS tree, except for *Venturiocistella*, a genus with no clear relationship in the ITS analysis. Based on the ITS tree, also in Han Clade 4 are *Soosiella* and *Leptodontidium* (both previously *incertae sedis*, and both sampled from the type specimen), and possibly *Catenulifera*, although this genus has no data available from the type species.

*Cordieritidaceae* mostly cluster in a poorly resolved clade in the ITS tree, and this clade includes the *incertae sedis* genera *Sabahriopsis* and *Macroskyttea*.

The ITS tree supports the congeneric relationship of *Xylogone* and *Scytalidium* suggested by Johnston et al. (2014). Only *Scytalidium* is treated in the 15-gene analysis and it has a poorly resolved position near the base of *Helotiales*.

*Phacidiaceae* forms a well-resolved clade in the ITS tree, and based on this tree two taxonomically poorly understood genera *Epicladonia* and *Fulvoflamma* might also belong in this family.

Based on our ITS tree several other genera could also possibly belong in the *Leotiales* sensu Fig. 6, including *Cochlearomyces* (*Cochlearomycetacae*, Crous et al. 2017), *Gorgomyces*, *Miniancora*, *Mycosymbioses*, and *Satchmopsis*. *Patinella* is close to *Holwaya* in the ITS tree. However, deeper relationships in this part of the ITS tree are poorly resolved and additional genes, additional taxa, and careful consideration of morphology is needed to develop a stable classification for *Leotiales*.

Other families not mentioned above that were sampled only with ITS data include *Cyttariaceae*, *Heterosphaeriaceae*, *Mitrulaceae*, and the recently described *Neocrinulaceae*. Based on the limited molecular data available, all occupy isolated positions within *Helotiales*, and whether or not they can be placed in one of our informal clades within *Helotiales*, or whether they form further distinct clades, requires increased gene sampling to resolve.

## TAXONOMY

**Marthamycetales** P.R. Johnst. & Baral, **ord. nov.**

MycBank No.: MB827852

*Etymology*: named after the type genus *Marthamyces*.

*Diagnosis*: Phylogenetically isolated within *Leotiomycetes*; differs from the micro-morphologically similar *Rhytismatales* by the apothecia being deeply immersed in host tissue and having granular crystalline material on the hymenial surface.

*Type*: *Marthamyces* Minter 2003.

*Description*: Apothecia erumpent from substrate, covering layer splitting into irregular lobes to expose the more or less flat hymenial surface, underside of the lobes and surface of the hymenium often associated with a granular exudate giving a white, yellowish or greenish tinge. Excipular tissue layers reduced, marginal lobes often with internal periphysoids. Paraphyses simple or often highly branched near the apex. Asci thin-walled or with thick apical dome, nonamyloid. Ascospores 1-several septate, ellipsoid to filiform, lacking a gel sheath. Asexual morph unknown.

*Habitat*: Saprobic on wood, bark, and leaves, desiccation-tolerant.

*Notes*: *Marthamycetales* is proposed here as a new order with the single family *Marthamycetaceae*. Our results confirmed the comment by Baral (2015, 2016) that this family is phylogenetically isolated within *Leotiomycetes*.

**Mniaeciaceae** Baral, **fam. nov.**

MycoBank No.: MB828888

*Etymology*: named after the type genus, *Mniaecia*.

*Diagnosis*: Phylogenetically sister to *Leotiaceae*, differs by the small, sessile discoid apothecia and in being parasitic on liverworts.

*Type*: *Mniaecia* Boud. 1885.

*Description*: Apothecia gymnohymenial, sessile, superficial, non-gelatinous, white or blue-green, with smooth, non-protruding margin, hairless; ectal excipuletextura prismatica-globulosa, without crystals. Paraphyses simple, without vacuolar bodies. Asci with ± conical, inamyloid, thick-walled apex. Ascospores hyaline, broadly ellipsoid, non-septate, without sheath, with high lipid content (multiguttulate). Asexual morph unknown.

*Habitat*: Parasitic on liverworts on soil, desiccation-intolerant.

*Included genera*: *Mniaecia* (syn. *Epiglia*).

*Notes*: This new family was discussed by Baral (2016) as the *Mniaecia* lineage. It has a sister relationship with *Tympanidaceae* and *Leotiaceae*, and with the latter it shares the characteristics of lipid-rich ascospores and growth on soil*.*

**Drepanopezizaceae** Baral **fam. nov.**

*Synonym*: *Drepanopezizaceae* Bat. & *H. Maia*, *Saccardoa* **1**: 98 (1960); nom. inval. (Art. 38.1)

MycoBank No.: MB828889

*Etymology*: named after the type genus, *Drepanopeziza*.

*Diagnosis*: Phylogenetically sister to *Ploettnerulaceae*, differs by the apothecia often being associated with stromatic structures; asexual morphs acervular, conidiogenesis holoblastic, and conidia large.

*Type*: *Drepanopeziza* (Kleb.) Jaap 1914.

*Description*: Apothecia sessile, immersed to erumpent, rarely superficial, often on stromatic tissue, hymenium greyish to brownish, margin often protruding, with or without lobes. Ectal excipule textura angularis. Paraphyses usually without vacuolar bodies. Asci with obtuse to conical apex, with or without amyloid apical ring. Ascospores ellipsoid to fusoid or often ± broadly ovoid-clavate, 0–1(− 2)-septate, septum ± eccentrical, lipid content low to medium. Asexual morph acervular, subcuticular, holoblastic, percurrent; conidia 0–1-septate, variously shaped, rarely staurosporous.

*Habitat*: Parasitic on leaves of various dicotyledons, causing leaf-spot diseases, rarely on herbaceous stems (*Spilopodia*); desiccation-tolerant.

*Included genera*: *Blumeriella* (syns. *Higginsia*, *Phloeosporella*, and *Microgloeum*), *Diplocarpon* (syn. *Marssonina*), *Drepanopeziza* (syn. *Gloeosporidiella*), *Felisbertia*, *Leptotrochila* (syns. *Ephelina, Fabraea,* and *Sporonema*), *Pseudopeziza*, *Spilopodia* (syns. *Holmiodiscus* and *Melanodiscus*), *Spilopodiella*, and *Thedgonia*.

*Notes*: The family was invalidly described (without a diagnosis or description) by Batista and Maia (1960) and is validated here. Our phylogenetic analyses support the treatment by Baral (2016) of this group of plant pathogenic fungi as distinct from the phylogenetically related *Ploettnerulaceae*. *Pseudopezicula* is excluded from the family as molecular data indicate affiliation in the *Discinella*-*Pezoloma* lineage.

## DISCUSSION

The genomic and 15-gene phylogenies presented here are the first multi-gene, class-wide analyses of *Leotiomycetes*. They provide a framework for taxon and specimen selection for the future, targeted studies that are needed to formally develop a complete, modern classification of *Leotiomycetes* that is congruent with molecular phylogenetic relationships. The development of such a classification will require additional genomes to be sequenced to provide sufficient data to allow deeper relationships to be accurately resolved. Our phylogenies will inform the selection of taxa that are needed to be sampled at this level to provide that extra level of resolution. In addition, the data on which our phylogenies are based provide a set of reliable, taxonomically annotated DNA sequences that can be used in the meantime for placing specimens in a taxonomically informal, phylogenetic context. Such phylogenetic placement may help in understanding ecosystem function of unnamed organisms detected.

We propose only a few taxonomic novelties in this paper, because our analyses have limitations in terms of both taxon sampling and depth of gene coverage. Consequently, our results on their own are often inadequate to propose definite changes in the current classification. The groups of labelled clades defined in the phylogenies are based solely on molecular phylogenetic relationships. Where they are inconsistent with existing classifications, the source of this inconsistency (e.g. problems with our data, or problems with the current selection of taxonomically informative morphological characters) needs to be investigated in detail.

Three data quality issues might impact the reliability of the phylogeny presented as Figs. 2, 3, 4, 5 and 6. Firstly, gene coverage across the alignment is patchy. Only 30 of the specimens treated have all 15-genes available (Additional file 5: Table S7). Missing data such as this has been shown to affect accuracy of ML trees (e.g. Xi et al. 2016). Where two specimens have no genes in common in the data matrix, a pair-wise distance measure cannot be made. In our data matrix only 0.13% of the pairwise distance comparisons between specimens are missing. Three of our specimens lacked a pair-wise distance measure to more than 25 other specimens in the dataset. In none of these cases (KL299, KL332, and ERTZ16200) was their position in the phylogeny unexpected. Conversely, those specimens on long branches, such as 604a, D2514, and DH267, generally had very little missing data, with three or fewer between-specimen distances missing. This ad hoc examination of our data suggests that missing genes had minimal impact on the topology of the phylogenetic tree presented.

A second data quality issue that is potentially more problematic is the number and presumed placement of missing taxa. Theory (Townsend and Lopez-Giraldez 2010), as well as earlier analyses in this study that were based a smaller set of taxa (unpubl. data), demonstrated that the position of specimens on long branches is particularly impacted by the addition of extra, closely related specimens. Therefore, the position of several taxa on long branches, such as the type species of *Cairneyella*, *Crocicreas* and *Cryptohymenium*, could potentially be revised with molecular phylogenetic data from additional species within these genera, or from species of closely related genera. Many genera are represented by only ITS data. The placement of these within the classification accepted here should be regarded as tentative. The ITS analysis was included because it treats many more genera than the more data-rich analyses. Many of genera seem to have clearly resolved relationships in the ITS phylogeny, and the results of this analysis help highlight some of the outstanding taxonomic issues requiring additional sequence data. In addition, the ITS section of the paper provides a collation of reliable ITS sequence data (downloadable from the Manaaki Whenua Datastore) that are available to use to place sequences from unidentified specimens or DNA extracts in an approximate phylogenetic structure.

The third issue relates to the taxonomic names attached to specimens. Only 22 of the taxa in the 15-gene analysis are represented by type specimens or ex-type cultures. A further 84 genera are represented by specimens identified as the type species of that genus. Whether or not those identifications are correct has not been assessed in this study. Several genera are represented only by non-type species, that is species that may or may not be congeneric with the type of that genus.

The increasing number of genomes available for this class is providing an increasingly powerful taxonomic resource. The cost of sequencing a genome is continuing to drop, and the informatics tools now available make the production of a draft assembly of suitable quality for phylogenetic analyses straightforward. Of our analyses, only the genome-scale phylogeny provided strong support for the deepest, backbone branches in the phylogeny. It is these deep relationships that require resolution to provide support for possible new taxa at the ordinal level within the class *Leotiomycetes*. Despite the increasing number of genomes for *Leotiomycetes*, not every family has been sampled, and toward the base of the phylogeny, important for the resolution of the deep backbone (Townsend and Lopez-Giraldez 2010), sampling depth is particularly poor. A genome-based project targeting authentically identified specimens that represent poorly sampled parts of the trees presented here, should provide the data necessary to produce a really strong, phylogenetically sound classification. Future analyses of gene function based on these genomes will provide new insights into how effectively phylogeny, and classification based on that phylogeny, are able to predict lifestyle of newly discovered species with an unknown ecology. Ideally, such a classification will help predict the behaviour, or properties of interest, of ecologically poorly understood taxa included in the various clades resolved.

The *Helotiales* clade as defined in our analyses (Figs. 1 and 2) includes about 80% of specimens that we treat in both the 15-gene and ITS analyses. This high level of gene and taxon sampling reflects the predominance of *Helotiales* in the formal taxonomy of *Leotiomycetes* (Quijada 2018). If orders such as *Cyttariales*, *Erysiphales* and *Medeolariales* are accepted, then *Helotiales* is not only molecular phylogenetically extremely broad but is also highly paraphyletic. This paraphyly means that as a taxon it provides little useful information about relationships. However, the genome-scale phylogeny (Fig. 1), although based on a limited number of taxa, suggests that strong molecular phylogenetic structure does exist within the *Helotiales* clade. With increased taxon sampling of genome-scale data, underlying structure could provide the basis for a new, phylogenetically informative order-level taxonomy for *Helotiales s.lat*.

The treatment of paraphyletic relationships, often recognized taxonomically for sets of species with highly divergent, specialised morphologies, cannot be resolved by more informative phylogenies. Some taxonomists and users of classifications are willing to accept paraphyly within a classification (e.g. Davydov et al. 2010), to avoid ballooning numbers of taxonomic names of limited practical use. For example, strict monophyly within the *Sclerotiniaceae* + *Rutstroemiaceae* clade in our phylogeny, would require *Rutstroemiaceae* to be split into four or more families, if *Sclerotiniaceae* was to be retained. *Sclerotiniaceae*, a clade of genetically similar, economically important nectrotrophic pathogens, is an extremely useful taxonomic concept for plant pathologists. Because of its value to users, there are strong reasons for *Sclerotiniaceae* to be retained. The disadvantage is that the genetic diversity of the species within *Rutstroemiaceae* is ‘hidden’ by the classification. This may in turn inhibit an understanding of the evolution of lifestyles amongst these fungi, an understanding that is of economic significance when the fungi include plant pathogens, or mutualists advantageous to plant growth. Applied studies investigating the evolution of fungal lifestyles requires an accurate understanding of phylogeny to allow selection of an informative set of species. Where this selection is being applied by mycologists who are naïve to the taxonomic concepts applied, classifications that include paraphyletic taxa have the potential to mislead rather than to inform.

The phylogenies presented here are intended as a step toward a phylogenetically informative classification of *Leotiomycetes*. As discussed above, the DNA sequence data needed to generate strongly resolved phylogenies is incomplete. A cost- and time-effective way of providing the data needed for robust phylogenies across the class as a whole, would be through the sequencing of additional, selected genomes. The data generated will be of greatest value when that selection considers both the specimens being sequenced along with the informativeness of the taxon for phylogenetic uncertainties to be resolved. Our phylogenies provide a resource for the selection of those highly informative taxa for existing genera and families. This informed taxon selection should be combined with deliberate specimen selection, the most accurately identified specimen being the type specimen, and the most valuable type specimens are those available from public, curated fungaria and culture collections. To ensure *Leotiomycetes* classifications represent the global diversity of these fungi, there is a further need to actively sample taxa from poorly explored parts of the world. The study of fungal taxonomy has historically been largely based in North temperate regions, thus the current formal taxonomy unavoidably has a strong bias toward explaining the better catalogued north temperate diversity, a diversity that excludes important tropical and southern lineages.

## CONCLUSIONS

The genomic and 15-gene phylogenies presented here provide a framework for taxon and specimen selection for the future targeted studies that are needed to formally develop a complete, modern classification of Leotiomycetes that is congruent with molecular phylogenetic relationships. The development of such a classification will require additional genomes to be sequenced to provide sufficient data to allow deep relationships to be accurately resolved. A strong, phylogenetically sound classification, combined with analyses of gene function based on the genomes used to resolve the phylogeny, will provide new insights into how effectively phylogeny, and classification based on that phylogeny, are able to predict lifestyle. Such a classification may help predict the behaviour, or properties of interest, of newly discovered species with an unknown ecology or other ecologically poorly understood taxa, and of unnamed taxa detected in ecological studies using molecular environmental sampling.

## Additional files

Additional file 1: **Table S1.** Specimens sampled, their taxonomic name, the family in which the genera were placed by Baral (2016) unless otherwise indicated, the family accepted based on the analysis in this paper, the sequences available for those specimens, either as Sanger sequences (with Genbank accesion number, otherwise available from alignments in https:/doi.org/10.7931/T5YV-BE95) or as extracts from genomes despoited in JGI and NCBI (Extracts available from alignments in https://doi.org/10.7391/T5YV-BE95). Newly generated sequences and genomes are in bold. (PDF 872 kb)

Additional file 2: **Table S2.** Sequences used in ITS phylogeny, Additional file 6: Figure S2. Accepted family based on ITS and 15-gene analyses in this paper. (PDF 761 kb)

Additional file 3: **Figure S1.** PhyDesign analysis. The upper image depicts the ultrametric tree for the taxa sampled using the single-copy orthologue dataset of Rokas et al. (2005) as modified by Taylor and Berbee (2006). Calibration points targeted the divergence of major *Pezizomycotina* lineages, approximately 250–400 million years ago, based on the fungal, animal and plant calibrations of Taylor and Berbee (2006). The lower image displays phylogenetic informativeness profiles for gene fragments from the datasets of Rokas et al. (2005), Taylor and Berbee (2006) and the AFTOL-1 project (Schoch et al. 2009). AFTOL-1 gene fragments are labeled with an asterisk (*). The five lesser-known fragments predicted to provide the greatest phylogenetic nformativeness for the desired epoch are also labeled. (PDF 456 kb)

Additional file 4: **Table S3.** The top 10 loci estimated for effectively resolving major nodes basal in the *Leotiomycetes* phylogeny based on the PhyDesign analysis (Additonal file 3: Figure S1). Those used in our analyses marked with *. (DOCX 13 kb)

Additional file 5: **Table S7.** Depth of gene coverage across the specimens sampled. (DOCX 12 kb)

Additional file 6: **Figure S2.** ML tree based on ITS sequences. Labels include Genbank accession number, voucher number, family name accepted by Baral (2016) in brackets (as “( - )” if not treated in that work), and family name accepted on the basis of the 15-gene (Figs. 2, 3, 4, 5 and 6) and ITS phylogenies. Bootstrap values > 50% are indicated. The phylogenetic tree is rooted with *Sarea* and *Tiarosporella*. The alignment used for this analysis and a nexus version of the tree can be downloaded from the Manaaki Whenua – Landcare Research datastore, see https://doi.org/10.7931/T5YV-BE95. (PDF 7796 kb)

Additional file 7: **Table S6.** Summary of the family level relationships of the genera treated, comparing the taxonomy of Baral (2016) with the relationships suggested in our analyses (genera where higher taxon has changed are in bold). Where a genus was not treated by Baral (2016) the family level classification provided by Index Fungorum in provided. Also provided is the type status of the specimens used in our analyses, and where the suggested relationship is based on the ITS analysis only. (PDF 566 kb)

Additional file 8: **Table S4.** Basic data on the draft genome assemblies of 10 Leotiomycetes specimens deposited as NCBI Bioproject PRJNA487672. Data compares results from two assemblers. (DOCX 19 kb)

Additional file 9: **Table S5.** Number of genes detected using BUSCO for each of the genomes used in genome phylogeny. Completeness value based on the BUSCO Pezizomycotina database (contains 3156 reference genes). (DOCX 19 kb)

## Abbreviations

5.8S: Ribosomal DNA 5.8S region
AFTOL-1: Assembling the Fungal Tree of Life project
EF1a: Elongation factor 1-alpha gene
IMC11: 11th International Mycological Congress in Puerto Rico
ITS: Ribosomal DNA internal transcribed spacer region
LSU: Ribosomal DNA large subunit region
MCM7: Minichromosome maintenance complex component 7 gene
ML: Maximum likelihood phylogenetic analysis
mtSSU: Mitochondrial small subunit gene
NCBI: National Center for Biotechnology Information
RPA1: Replication protein A 70 KDa DNA-binding subunit gene
RPA2: Replication protein A 32 KDa subunit gene
RPB1: DNA-directed RNA polymerase II subunit 1 gene
RPB2: DNA-directed RNA polymerase II subunit 2 gene
RPC2: DNA-Directed RNA polymerase III 127.6 KDa polypeptide gene
SF3B1: Splicing factor 3B subunit 1 gene
SSU: Ribosomal DNA small subunit region
TFB4: General transcription and DNA repair factor IIH subunit gene
