# Supplementary material for: A multigene phylogeny toward a new phylogenetic classification of Leotiomycetes
Source: IMA Fungus. 2019 Jun 7;10:1. doi: 10.1186/s43008-019-0002-x (PMC7325659; doi:10.1186/s43008-019-0002-x)
Supplement: Supplementary file 1 — Table S1. Specimens sampled, their taxonomic name, the family in which the genera were placed by Baral (2016) unless otherwise indicated, the family accepted based on the analysis in this paper, the sequences available for those specimens, either as Sanger sequences (with Genbank accesion number, otherwise available from alignments in https:/doi.org/10.7931/T5YV-BE95) or as extracts from genomes despoited in JGI and NCBI (Extracts available from alignments in https://doi.org/10.7391/T5YV-BE95). Newly generated sequences and genomes are in bold. (PDF 872 kb) [file 43008_2019_2_MOESM1_ESM.pdf]

Suppl Data Table S1. Specimens sampled, their taxonomic name, the family in which the genera were placed by Baral (2016) unless otherwise indicated, the family accepted based on the analyses in this paper, and the sequences available for those specimens, either as Sanger sequences (with Genbank accession number, otherwise available from alignments in https://doi.org/10.7931/T5YV-BE95) or as extracts from genomes despoited in JGI and NCBI (extracts available from alignments in https://doi.org/10.7931/T5YV-BE95). Newly generated sequences and genomes are in bold.

Code	species	current higher taxon (1)	Accepted higher taxon	type status (2)	Voucher or strain	NCBI genome biosample	JGI genome	notes	SSU	ITS	LSU	RPB1	RPB2	RPA1	mtSSU	TEF1	Btub	MCM7	RPC2	TFB4	RPA2	a-tub	SF3B1
CBS109321	Acephala applanata	Mollisiaceae	Mollisiaceae	exType specimen	CBS 109321					NR_119482	KF951051	KT225541											
Acema1	Acephala macrosclerotiorum	Mollisiaceae	Mollisiaceae		CBS 123555		Acema1 (12)			NR_121349		genome	genome	genome		genome	genome	genome	genome	genome	genome	genome	genome
DSM104360	Alatospora acuminata	Leotiaceae (5)	Leotiales incertae sedis	Type species	DSM 104360 (6)			= CCM-F12186	**MK226454**	AY204588	**MK226457**	**MK241480**	**MK241444**			**MK241454**	**MK241467**						
A_acu_Hosoya	Albotricha acutipila	Lachnaceae	Lachnaceae	Type species	FC-2262, TNS-F-16740 (7)			ITS of specimen ex Japan a little different to KT005452 (ex Sweden - type locality)	**LC434582**	AB481234	AB481317	**LC431679**	AB481354		**LC431751**		**MWLR Datastore**						
A_alb_Hosoya	Albotricha albotestacea	Lachnaceae	Lachnaceae		FC-2094, TNS-F-16497 (7)					AB481235	AB481303		AB481340		**LC431747**		**MWLR Datastore**						
KL391	Ameghiniella australis	Cordieriticaeae	Cordieriticaeae	Type species	FH 01146515				KX090893	**MH752070**	KX090841	KX090787				KX090690							
F51377	'Amicodisca' castaneae		Helotiales (Han Clade 7)		KUS F51377					JN033389	JN086692		JN086843		JN086766								
Amore1	Amorphotheca resinae	Amorphothecaceae	Amorphothecaceae		ATCC 22711		Amore1 (4, 13)		genome	EU030278	EU030280	genome	genome	genome	genome	genome	genome	genome	genome	genome	genome	genome	genome
D368	Aotearoamyces nothofagi	Tympanidaceae	Leotiales incertae sedis	Type species	ICMP 21969 (8)				**MG807391**	**KM677201**	**MG807387**							**MH700578**					
D781	Arachnopeziza araneosa	Arachnopezizaceae	Arachnopezizaceae		ICMP 21731 (8)	**SAMN09907757** (3, 4)			genome	**MH578553**	genome	**MH682248**	genome	genome	genome	genome	genome	**MH700571**	genome	genome	genome	genome	genome
TNS_F11212	Arachnopeziza aurata	Arachnopezizaceae	Arachnopezizaceae		TNS-F-11212 (7)				**LC434570**	JN033436	**AB546936**		JN086881		JN086806								
A_aurel_Hosoya	Arachnopeziza aurelia	Arachnopezizaceae	Arachnopezizaceae	Type species	TNS-F-40009 (7)				**LC434591**	**LC425050**	**LC429386**	**LC431691**	JN086880		JN086805								
TNS_F12768	Arachnopeziza obtusipila	Arachnopezizaceae	Arachnopezizaceae		TNS-F-12768 (7)				**LC434571**	JN033445	JN086746	**LC431670**	JN086890		JN086815	**MWLR Datastore**							
DSM104345	Articulospora tetracladia	Discinella-Pezoloma lineage	Discinella-Pezoloma lineage	Type species	DSM 104345 (6)				**MK226453**	**MK226461**	**MK226456**	**MK241471**	**MK241436**			**MK241447**	**MK241460**						
KUS_F52351	Ascocoryne cylichnium	Gelatinodiscaceae	Gelatinodiscaceae		TNS-F-15227 (7)				**LC434577**	JN033406	JN086709	**LC431673**			JN086782								
Ascsa1	Ascocoryne sarcoides	Gelatinodiscaceae	Gelatinodiscaceae	Type species	NRRL 50072		Ascsa1 (4, 28)		genome	genome	genome	genome	genome	genome	genome	genome	genome	genome	genome	genome	genome	genome	genome
D369	Ascocoryne sp.	Gelatinodiscaceae	Gelatinodiscaceae		ICMP 22539 (8)					**MH682237**		**MH682251**						**MH700579**					
Blugr1	Blumeria graminis f.sp. hordei	Erysiphaceae	Erysiphaceae	Type species	DH14		Blugr1 (4, 14)		genome	genome		genome	genome	genome	genome	genome	genome	genome	genome	genome	genome	genome	genome
Botci1	Botrytis cinerea	Sclerotiniaceae	Sclerotiniaceae	Type species	B05.10		Botci1 (4, 15)		genome			genome	genome	genome	genome	genome	genome	genome	genome	genome	genome	genome	genome
KUS_F52031	Brunnipila fuscescens	Lachnaceae	Lachnaceae		KUS-F52031; TNS-F-16635 (7)				**LC434580**	JN033392	**LC424945**	**LC431677**	JN086846		**LC431750**		**MWLR Datastore**						
M141	Bryoscyphus dicrani	Helotiaceae	Helotiaceae		TUR 185318				EU940034	EU940183	EU940107		EU940323										
AFTOL_ID_916	Bulgaria inquinans	Phacidiaceae	Phacidiaceae	Type species	CBS 118.31				DQ471008	KJ663831	DQ470960	DQ471152	DQ470910			DQ471079							
Bulin1	Bulgaria inquinans	Phacidiaceae	Phacidiaceae	Type species	CBS 118.31		Bulin1 (4, 25)					genome	genome	genome		genome	genome	genome	genome	genome	genome	genome	genome
TL2011	Bulgariella pulla	incertae sedis	Helotiaceae	Type species	TL2011				KU845534	KU845537	KU845535		KU845545			KU845541							
Mo12	Cadophora malorum	Ploettnerulaceae	Ploettnerulaceae		Mo12	SAMEA3925016			genome	genome	genome	genome	genome	genome	genome	genome	genome	genome	genome	genome	genome	genome	genome
Cadsp1	Cadophora sp DSE1049	Ploettnerulaceae	Ploettnerulaceae		DSE1049		Cadsp1 (4, 16)		genome	genome	genome	genome	genome	genome		genome	genome	genome	genome	genome	genome	genome	genome
VPRI42388	Cairneyella variabilis	Helotiales incertae sedis (5)	Helotiales (hyaloscyphoid clade)	exType specimen	CSIRO, FRR 6070	SAMN04263868 (4)			genome	KF836358	genome	genome	genome	genome	genome	genome	genome	genome	genome	genome	genome	genome	genome
HMAS187063	Calycellinopsis xishuangbanna	Dermateaceae (5)	Cenangiaceae	Type species	HMAS 187063 (9)				GU936124		KR094163	**MH729338**	**MH729345**										
AFTOL_ID_1301	Calycina citrina	Pezizellaceae	Pezizellaceae		CBS 139.62			as Bisporella citrina	FJ176815		FJ176871		J238354		FJ190632								
D702	Calycina citrina	Pezizellaceae	Pezizellaceae		ICMP 22538 (8)					**MH682235**		**MH682243**		**MH700581**				**MH700575**					
B_disc_Hosoya	Calycina discedens	Pezizellaceae	Pezizellaceae		TNS-F-37025 (7)			as Bisporella discedens	**LC434589**	LC169492	**LC429377**	**LC431690**	**LC431724**		**LC431758**								
CBS_466_73	'Calycina herbarum'		Helotiaceae		CBS 466.73	SAMN04158973		Hymenoscyphus sp., genetically distinct from Calycina				genome	genome		genome	genome	genome	genome	genome	genome	genome	genome	genome
Bisps1	Calycina sp.	Pezizellaceae	Pezizellaceae		PMI 857		Bisps1 (4, 12)	as Bisporella sp.	genome	genome	genome	genome	genome	genome		genome	genome	genome	genome	genome	genome	genome	genome
KL174	Cenangiopsis quercicola	Cenangiaceae	Cenangiaceae	Type species	TAAM 178677				KX090862	LT158425	KX090811	KX090760	KX090713			KX090663							
KL243	'Cenangium' acuum		Rutstroemiaceae		TAAM 198515				KX090873	LT158439	KX090822	KX090767	KX090720			KX090674							
KL390	Cenangium ferruginosum	Cenangiaceae	Cenangiaceae	Type species	TAAM 198451				KX090892	LT158471	KX090840		KX090739										
BPI843553	Chaetomella oblonga	Chaetomellaceae	Chaetomellaceae	Type species	CBS 110.76				AY487084	AY487082	AY487083												
Chalo1	Chalara longipes	Pezizellaceae	Hyaloscyphaceae		BDJ		Chalo1 (4, 12)	based on SSU, could be congeneric with AF203463, a putative Chalara fusarioides	genome	genome	genome	genome	genome	genome			genome	genome	genome	genome	genome	genome	genome
D1686	Chlorencoelia torta	Cenangiaceae	Cenangiaceae		ICMP 21732 (8)	**SAMN09907751** (3, 4)			genome	**MH682234**	genome	genome	genome	genome	genome	genome	genome	genome	genome	genome	genome	genome	genome
KP606	Chlorencoelia versiformis	Cenangiaceae	Cenangiaceae	Type species	TU 107606				KX090894			KX090788	KX090740			KX090692							
IHIA39	Chlorociboria aeruginascens	Chlorociboriaceae	Chlorociboriaceae		IHIA39	SAMN06706673 (4)			genome	genome	genome	genome	genome	genome	genome	genome	genome	genome	genome	genome	genome	genome	genome
C_aerugascens_Hosoya	Chlorociboria aeruginascens	Chlorociboriaceae	Chlorociboriaceae		TNS-F-36241 (7)				**LC434588**	**LC425045**	**LC429376**	**LC431689**	**LC431723**										
KL247	Chlorociboria aeruginella	Chlorociboriaceae	Chlorociboriaceae		TAAM 198514 (10)				KX090875	**MH752067**		KX090769	KX090722			KX090676							
AFTOL_ID_151	Chlorociboria aeruginosa	Chlorociboriaceae	Chlorociboriaceae	Type species	OSC 100056				AY544713	DQ491501	AY544669	DQ471125	DQ470886		AY544734	DQ471053							
C_aerugnosa_Hosoya	Chlorociboria aeruginosa	Chlorociboriaceae	Chlorociboriaceae		TNS-F-13596 (7)				**LC434578**	**LC425047**	**LC429383**	**LC431687**											
D1530	Chlorociboria halonata	Chlorociboriaceae	Chlorociboriaceae		ICMP 15625				JN939862	AY755355	JN939933	JN985211	JN985511					JN993298					
D788	Chlorociboria poutoensis	Chlorociboriaceae	Chlorociboriaceae		ICMP 15618 (8)					AY755352		**MH682247**						**MH700576**					
FUNNZ2018_2904	Chlorociboriaceae sp.		Chlorociboriaceae		PDD 112225				**MK248054**	**MK248036**	**MK248011**	**MK241482**	**MK241484**					**MK241483**					
AFTOL_ID_67	Chloroscypha cf. enterochroma	Gelatinodiscaceae	Gelatinodiscaceae		OSC 100020				AY544700		AY544656				AY544735								
HMAS275558	Chlorosplenium chlora	incertae sedis	Mollisiaceae	Type species	HMAS 275558 (9)			specimen from China, LSU differs slightly from C. chlora ex N America ex Genbank	**MH729331**		**MH729335**	**MH729340**	**MH729347**										
C_bat_Hosoya	Ciboria batschiana	Sclerotiniaceae	Sclerotiniaceae		TNS-F-44241 (7)			SSU and RPB1 from TNS F40078	**LC434592**	AB926056	AB926143	**LC431686**	AB926188				**MWLR Datastore**						
KL365	Ciboria batschiana	Sclerotiniaceae	Sclerotiniaceae		TU 104222 (10)				**MH758758**	LT158466	**MH748088**												
ICMP19812	Ciborinia camelliae	Sclerotiniaceae	Sclerotiniaceae		ICMP 19812	SAMN03840770 (4)			genome	genome		genome	genome	genome	genome	genome	genome	genome	genome	genome	genome	genome	genome
1927H	Ciborinia whetzelii	Sclerotiniaceae	Sclerotiniaceae	Type species	1927.H (Oslo)				Z73714	Z73768	Z73742												
F52678	Cistella albidolutea	Hyaloscyphaceae	Helotiales (Stamnaria lineage/Han Clade 9)		KUS-F52678					JN033429	JN086732		JN086872		JN086798								
HRI11	Clarireedia homeocarpa	Rutstroemiaceae	Rutstroemiaceae		HRI11	SAMN04267405					genome	genome	genome	genome	genome	genome	genome	genome	genome	genome	genome	genome	genome
C_atro_Hosoya	Claussenomyces prasinulus	Tympanidaceae	Leotiales incertae sedis		TNS-F-18149 (7)				**MWLR Datastor**	**LC425048**	**LC429384**	**LC431674**	**LC431719**										
KL218	Claussenomyces prasinulus	Tympanidaceae	Leotiales incertae sedis		TU 104191				KX090866		KX090815					KX090667							
AFTOL_ID_147	Coccomyces dentatus	Rhytismataceae	Rhytismataceae		OSC 100021				AY544701	DQ491499	AY544657		DQ247789		AY544736	DQ497605							
Cocst1	Coccomyces strobi	Rhytismataceae	Rhytismataceae		CBS 202.91		Cocst1 (4, 25)	AFTOL-ID 1250	DQ471027	genome	DQ470975	genome	genome	genome	FJ190622	genome	genome	genome	genome	genome	genome	genome	genome
BP5796	Coleophoma crateriformis	Leotiomycetes incertae sedis (5)	Dermateaceae	Type species	BP5796	SAMN07177961						genome	genome	genome			genome	genome	genome	genome	genome	genome	genome
BP6252	Coleophoma cylindrispora	Leotiomycetes incertae sedis (5)	Dermateaceae		BP6252	SAMN07195328						genome	genome	genome	genome		genome	genome	genome	genome	genome	genome	genome
CBS120878	'Collophorina' paarla		Leotiales incertae sedis		CBS 120878				GQ154632	GQ154575	GQ154611												
CBS120873	Collophorina rubra	Tympanidaceae	Tympanidaceae	exType specimen	CBS 120873				GQ154627	GQ154547	GQ154606												
AFTOL_ID_1264	Connersia rilstonii	Pseudeurotiaceae	Helotiaceae	exType specimen	CBS 537.74				AF096174	KJ755499	FJ176866	FJ238435	KJ755473		FJ190625	FJ238398							
192	Cordierites guianensis	Cordieriticaeae	Cordieriticaeae	Type species					EU107262		EU107270												
AFTOL_ID_272	Crinula caliciiformis	Tympanidaceae	Leotiomycetes incertae sedis	Type species	DAOM 231117			anamorphic Holwaya	AY544729	KT225524	AY544680		KT225542		AY544738								
604a	Crocicreas gramineum	incertae sedis	Helotiales (pezizelloid clade)	Type species	YSU-F-03527 (11)					**MH733945**	**MH733950**	**MK244982**						**MK244987**					
D2514	Cryptohymenium pycnidiophorum	Dermateaceae (5)	Helotiales (sclerotinioid clade)	type species	PDD 111534					**MH578491**	**MH985296**	**MH986707**	**MH986703**					**MH986710**					
CBS_268_39	Cryptosporiopsis nigra	Dermateaceae	Dermateaceae	Type species	CBS 268.39			as P. ocellata teleomorph of C. nigra, type of Cryptosporiopsis		KR859232	KR859024		KR859339				KR859302						
AFTOL_ID_166	Cudoniella clavus	Helotiaceae	Helotiaceae		OSC 100054				DQ470992	DQ491502	DQ470944	DQ471128	DQ470888		FJ713604	DQ471056							
AFTOL_ID_1296	Cyclaneusma minus	Marthamycetaceae	Marthamycetaceae	exType specimen	CBS 496.73				FJ176812	NR_153910	FJ176868		FJ238351		FJ190629	FJ238400							
R930	Cyclaneusma minus	Marthamycetaceae	Marthamycetaceae		ICMP 17358 (8)				KJ606669	KJ606680	KJ606674		**MH700583**		**MH698452**								
D_long_Hosoya	Dasyscyphella longistipitata	Lachnaceae	Lachnaceae		FC-2013 (7)			SSU, mtSSU and Btub from FC-2018 (TNS-F-16433)	**LC434581**	AB481239	AB481294		AB481331		**LC431745**		**MWLR Datastore**						
F17834	Dematioscypha dematiicola	Hyaloscyphaceae	Helotiales (Han Clade 7)		TNS-F-17834					JN033438	JN086739		JN086883		JN086808								
AFTOL_ID_941	Dermea acerina	Dermateaceae	Dermateaceae		CBS 131.68				DQ247809	AF141164	DQ247801	DQ471164	DQ247791		DQ976373	DQ471091							
10106	Dicephalospora rufocornea	Helotiaceae	Helotiaceae		HMAS 275559 (9)				**MH729332**	KU668565	**MH729336**	**MH729341**	**MH729348**										
D_ruf_Hosoya	Dicephalospora rufocornea	Helotiaceae	Helotiaceae		TNS-F-40024 (7)				**LC434557**	AB926055	AB926123	**LC431684**	AB926193				**MWLR Datastore**						
KL317	Diplocarpa bloxamii	Cordieritidaceae	Cordieritidaceae		C-F74826				KX090885			KX090834	KX090745			KX090688							
NL1	Diplocarpon mali	Drepanopezizaceae	Drepanopezizaceae		NL1	SAMN06564146 (4)		as Marssonina coronariae	genome	KY672995		genome	genome	genome		genome	genome	genome	genome	genome	genome	genome	genome
DortE4	Diplocarpon rosae	Drepanopezizaceae	Drepanopezizaceae	Type species	DortE4	SAMN06163654 (4)			genome	genome	genome	genome	genome	genome	genome	genome	genome	genome	genome	genome	genome	genome	genome
M168	Discinella schimperi	Discinella-Pezoloma lineage	Discinella-Pezoloma lineage		TUR 185323				EU940043	EU940192	EU940116		EU940328		EU940265								
KL111	'Encoelia' fimbriata		Cordieritidaceae		TAAM 165728				KX090852	**MH752063**	KX090800		KX090703			KX090655							
KL107	Encoelia furfuracea	Cenangiaceae	Cenangiaceae	Type species	TAAM 165633				KX090850	LT158416	KX090798	KX090749	KX090701			KX090653							
KL164	'Encoelia' heteromera		Cordieritidaceae		HB 8448				KX090861		KX090809	KX090758	KX090712			KX090662							
M193	Epiglia gloeocapsae	Mniacea lineage	Mniaceaceae	type species	TUR 185311				EU940055	EU940204	EU940128		EU940341		EU940272								
Erynec	Erysiphe necator	Erysiphaceae	Erysiphaceae		strain c		Erynec1 (4, 17)		genome			genome	genome	genome		genome	genome	genome	genome	genome	genome	genome	genome
CCM_F00681	Filosporella cf. annelidica		Helotiaceae		CCM F-00681 (6)				**MK226452**	**MK226458**	**MK226455**	**MK241473**	**MK241438**			**MK241449**							
CCM_F13091	Filosporella fistucella	Ascomycota incertae sedis (5)	Helotiales incertae sedis		CCM F-13091 (6)				**MK226451**	KC834047	KC834021	**MK241477**				**MK241456**	**MK241462**						
CCM_F_18699	Flagellospora curvula	Nectriaceae (5)	Leotiales incertae sedis	Type species	CCM F-18699 (6)			ITS and LSU ex CCM F-20899	**MK226450**	KC834050	KC834023	**MK241479**				**MK241453**	**MK241468**						
T6G9	Gamarada debralockiae	Hyaloscyphaceae (5)	Helotiales (Han Clade 4)	exType specimen	CSIRO, FRR 6190	SAMN07652639			genome	genome	genome	genome	genome	genome		genome	genome	genome	genome	genome	genome	genome	genome
G_ast_Hosoya	Gelatinipulvinella astraeicola	Helicogoniaceae	Helicogoniaceae	Type species	TNS-F-17846 (7)				**LC434573**	**LC425040**	**LC429381**	**LC431667**	**LC431715**		**LC431762**								
G_fung_Hosoya	Gelatinopsis fungicola	Helicogoniaceae	Helicogoniaceae		TNS-F-44003 (7)				**LC434551**	**LC425051**	**LC429387**	**LC431666**	**LC431725**		**LC431765**								
Glalo1	Glarea lozoyensis	Helotiaceae	Helotiaceae	exType specimen	ATCC 20868		Glalo1 (4, 18)		genome	NR_137138	genome	genome	genome	genome	genome	genome	genome	genome	genome	genome	genome	genome	genome
KL150	Godronia fuliginosa	Godroniaceae	Godroniaceae		HB 6640 (10)					**MH752064**	**MH748090**	**MH753364**											
Golci1	Golovinomyces cichoracearum	Erysiphaceae	Erysiphaceae		UCSC1		Golci1 (4, 26)		genome		genome	genome	genome	genome		genome	genome	genome	genome	genome	genome	genome	genome
Greab	Gremmeniella abietina	Godroniaceae	Godroniaceae	Type species	DAOM 170408		Greab1 (4, 29)	separate Asian and European haplotypes	genome	KC352960	genome	genome	KC533103	genome		genome	genome	genome	genome	genome	genome	genome	genome
CCM_F400	Gyoerffyella rotula	Discinella-Pezoloma lineage	Discinella-Pezoloma lineage		CCM F-400 (6)				**MK226449**	KC834061	KC834029	**MK241469**	**MK241434**			**MK241445**	**MK241458**						
TNS_F24336	Hamatocanthoscypha laricionis	Pezizellaceae	Pezizellaceae	Type species	TNS-F-24336				JN086830	JN033455	JN086755		JN086905			**MWLR Datastore**							
D2211	Helotiaceae sp.		Helotiaceae		ICMP 22540 (8)				**MH682222**	**MH682236**	**MH682242**		**MH700585**			**MH700586**		**MH700568**					
KL20	Heyderia abietis	Cenangiaceae	Cenangiaceae	Type species	TAAM 165961				KX090845	LT158426		KX090747	KX090699			KX090650							
KL216	Heyderia pusilla	Cenangiaceae	Cenangiaceae		TU 104257				KX090865	LT158430		KX090762	KX090715			KX090665							
D1881	Hispidula tokerau	Hyaloscyphaceae	Helotiaceae		ICMP 22800 (8)					**MH578493**		**MH682244**						**MH700567**					
H_muc_Hosoya	Holwaya mucida	Tympanidaceae	Leotiomycetes incertae sedis	Type species	TNS-F-29145 (7)				**LC434585**	**LC425042**	**LC429385**	**LC431688**	**LC431716**		**LC431763**								
TU112863	Holwaya mucida	Tympanidaceae	Leotiomycetes incertae sedis	Type species	TU 112863 (10)				KX090898	**MH752062**	KX090844	KX090793	KX090744										
KACC45613	Hyalopeziza nectrioides	Hyaloscyphaceae	Hyaloscyphaceae		KACC 45613					JN033381	JN086684		JN086836		JN086761								
M220	Hyaloscypha vitreola	Hyaloscyphaceae	Hyaloscyphaceae	Type species	TUR (S. Huhtinen 05/71)					FJ477059	FJ477058						FJ477054						
H_caud_Hosoya	Hymenoscyphus caudatus	Helotiaceae	Helotiaceae		TNS-F-40056 (7)				**LC434560**	AB926065	AB926134		AB926195		**LC431760**		**MWLR Datastore**						
CBS133217	Hymenoscyphus fraxineus	Helotiaceae	Helotiaceae		CBS 133217	SAMN04145411			genome	genome	genome	genome	genome	genome	genome	genome	genome	genome	genome	genome	genome	genome	genome
CBS_650_92	Hymenoscyphus fructigenus	Helotiaceae	Helotiaceae	Type species	CBS 650.92	SAMN04157984			genome	GU586933		genome	genome	genome	genome	genome	genome	genome	genome	genome	genome	genome	genome
H_fruct_Hosoya	Hymenoscyphus fructigenus	Helotiaceae	Helotiaceae	Type species	TNS-F-44644 (7)				**LC434563**	AB926057	AB926144		AB926189		**LC431756**								
KUS_F51352	Hymenoscyphus ginkgonis	Helotiaceae	Helotiaceae		KUS F51352 (7)			SSU from TNS-F-11208	**LC434552**	NR_119669	EU096525												
CBS122016	Hymenoscyphus infarciens	Helotiaceae	Helotiaceae		CBS 122016	SAMN04145410			genome		genome	genome	genome	genome	genome	genome	genome	genome	genome	genome	genome	genome	genome
D358	'Hymenoscyphus' ohakune		Helotiaceae		ICMP 19601 (8)				**MH682221**	NR_137109	**MH682241**	**MH682245**											
CBS_341_76	'Hymenoscyphus' repandus		Helotiaceae		CBS 341.76	SAMN04145415			genome		genome	genome	genome	genome	genome	genome	genome	genome	genome	genome	genome	genome	genome
CBS111550	Hymenoscyphus salicellus	Helotiaceae	Helotiaceae		CBS 111550	SAMN04145413			genome	genome	genome	genome	genome	genome	genome	genome	genome	genome	genome	genome	genome	genome	genome
CBS_480_97	Hymenoscyphus scutula	Helotiaceae	Helotiaceae		CBS 480.97	SAMN04151791			genome	KC481695		genome	genome	genome	genome	genome	genome	genome	genome	genome	genome	genome	genome
H_scut_Hosoya	Hymenoscyphus scutula	Helotiaceae	Helotiaceae		TNS-F-17507 (7)					AB926114	AB926118		AB926175		**LC431753**		**MWLR Datastore**						
H_var_Hosoya	'Hymenoscyphus' varicosporoides		Helotiaceae		FC-2038; TNS-F-16472 (7)				**LC434566**	AB481291	AB481292	**LC431678**	AB481329		**LC431746**								
Hymvar1	'Hymenoscyphus' varicosporoides		Helotiaceae				Hymvar1 (4, 12)		genome	genome	genome	genome	genome	genome		genome	genome	genome	genome	genome	genome	genome	genome
H_men_Hosoya	Hymenoscyphus virgultorum	Helotiaceae	Helotiaceae		TNS-F-40052 (7)				**MWLR Datastor**	AB926063	AB926132		AB926211										
D1086	Hymenotorrendiella dingleyae	Helotiaceae	Helotiaceae		ICMP 22793 (8)	**SAMN09907749** (3, 4)			genome	**MH578484**	genome	genome	genome	genome	genome	genome	genome	genome	genome	genome	genome	genome	genome
TNS_F13588	Hyphodiscus hyaloscyphoides	Hyaloscyphaceae	Helotiales (Han Clade 4)		TNS-F-13588 (7)				**LC434569**	AB546944	AB546945	**LC431668**	JN086892										
D1413	Hyphodiscus sp.	Hyaloscyphaceae	Helotiales (Han Clade 4)		ICMP 21723 (8)	**SAMN09907750** (3, 4)			genome	**MH682230**	genome	genome	genome	genome	genome	genome	genome	genome	genome	genome	genome	genome	genome
TNS_F17940	Hyphopeziza pygmaea	Hyaloscyphaceae	Helotiales (Han Clade 4)	Type species	TNS F17940					JN033448	JN086748		JN086894		JN086819								
TTT414	Hypoderma cordylines	Rhytismataceae	Rhytismataceae		ICMP 16705 (8)					**MH682238**		**MH682254**						**MH700580**					
R156	Hypoderma rubi	Rhytismataceae	Rhytismataceae	Type species	ICMP 17339 (8)				**MH682224**	JF683419	HM140526				HM143801								
I_rad_Hosoya	Incrucipulum radiatum	Lachnaceae	Lachnaceae		FC-2283, TNS-F-16769 (7)				**LC434584**	AB481261	AB481322		AB481359		**LC431752**		**MWLR Datastore**						
CBS_175_74	Infundichalara microchona	incertae sedis	Pezizellaceae	exType specimen	CBS 175.74				HQ609486	KR859078	HQ609479		KR859318				KR859284						
KL299	Ionomidotis frondosa	Cordieriticaeae	Cordieriticaeae		TNS-F-45265				KX090882			KX090775				KX090685							
KL231	Ionomidotis fulvotingen	Cordieriticaeae	Cordieriticaeae		TU 104510				KX090870		KX090819	KX090765	KX090719			KX090671							
KL154	Ionomidotis irregularis	Cordieriticaeae	Cordieriticaeae	Type species	TAAM 198520 (10)			specimen HB 8233	KX090856	**MH752065**	KX090804	KX090754				KX090658							
KL301	Ionomidotis olivascens	Cordieriticaeae	Cordieriticaeae		TNS-F-39269 (10)				KX090883	**MH752068**	KX090833	KX090776	KX090732			KX090686							
FC_2354	Lachnellula subtilissima	Lachnaceae	Lachnaceae		FC-2354					AB481247	AB481326		AB481363										
KL364	Lachnellula subtilissima	Lachnaceae	Lachnaceae		TU 104479 (10)				**MH758757**	**MH752069**	**MH748087**												
KL362	Lachnellula willkommii	Lachnaceae	Lachnaceae		TU 104064 (10)				**MH758756**		**MH748086**												
KUS_F52080	Lachnum abnorme	Lachnaceae	Lachnaceae		KUS-F52080 (7)			SSU and RPB1 from TNS-F-16617	**LC434554**	JN033395	JN086698	**LC431676**	JN086849		JN086772		**MWLR Datastore**						
D664	Lachnum nothofagi	Lachnaceae	Lachnaceae		ICMP 21728 (8)	**SAMN09907754** (3, 4)			genome	**MH682233**	genome	**MH682252**	**genome**	genome	genome	genome	genome	**MH700570**	genome	genome	genome	genome	genome
L_palm_Hosoya	Lachnum palmae	Lachnaceae	Lachnaceae		TNS-F-13500 (7)				**LC434575**	**LC425039**	**LC429382**		**LC431718**										
FC2137	Lachnum virgineum	Lachnaceae	Lachnaceae		FC-2137, TNS-F-16583 (7)				**LC434553**	AB705235	AB705275		AB481343		**LC431748**		**MWLR Datastore**						
AFTOL_ID_49	Lachnum virgineum	Lachnaceae	Lachnaceae		OSC 100002					DQ491485	AY544646.1	DQ842030	DQ470877		AY544745	DQ497602							
L_brun_Hosoya	'Lambertella' brunneola		Rutstroemiaceae		TNS-F-44244 (7)				**LC434593**	**LC425046**	**MWLR Datastore**	**LC431692**	**LC431720**										
HMAS_Lamb	Lambertella corni-maris	Rutstroemiaceae	Rutstroemiaceae	Type species	HMAS 279664 (9)				**MH729333**		KR094168	**MH729342**											
L_corn_Hosoya	Lambertella corni-maris	Rutstroemiaceae	Rutstroemiaceae	Type species	TNS-F-40083 (7)				**LC434562**	AB926069	AB926139		AB926184		**LC431755**								
L_pyr_Hosoya	Lambertella pyrolae	Rutstroemiaceae	Rutstroemiaceae		TNS-F-40132 (7)				**LC434565**	AB926081	AB926164		AB926209										
AFTOL_ID_1262	'Lambertella' subrenispora		Rutstroemiaceae		CBS 811.85				KF545534	KF545329			AB926220			DQ471101	KF545224	KF545466					
D804	Lanzia allantospora	Rutstroemiaceae	Rutstroemiaceae		ICMP 15649 (8)					AY755334								**MH700573**					
B_lon_hosoya	Lasiobelonium lonicerae	Lachnaceae	Lachnaceae		FC-2270; TNS-F-16667 (7)			Lasiobelonuium lonicerae = Belonidium lonicerae fide Species Fungorum	**LC434583**	AB481284	AB481319	**LC431680**	AB481356				**MWLR Datastore**						
CCM_F21799	Lemonniera aquatica	incertae sedis	Discinella-Pezoloma lineage	Type species	CCM F-21799 (6)				**MK226448**	**MK226460**	DQ267627	**MK241472**	**MK241437**			**MK241448**	**MK241461**						
AFTOL_ID_1	Leotia lubrica	Leotiaceae	Leotiaceae	Type species	OSC 100001					DQ491484	AY544644	DQ471113	DQ470876		AY544746	DQ471041							
Leptod1	'Leptodontidium' sp. PMI412		Ploettnerulaceae		PMI412		Leptod1 (4, 25)	Based on ITS ex CBS 329.53 (ExType culture), Leptodontidium is genetically distinct from PMI412. Ribosomal not available from the isolate with a genome, only ribosomal genes available from reliable identified isolates.				genome	genome	genome		genome	genome	genome	genome	genome	genome	genome	genome
AFTOL_ID_1397	Leuconeurospora pulcherrima	Pseudeurotiaceae	Pseudeurotiaceae	exType specimen	CBS 343.76				FJ176828	KF049206	FJ176884	FJ238440	FJ238367		FJ190639	FJ238409							
01NH01	Leuconeurospora sp 01NH01	Pseudeurotiaceae	Pseudeurotiaceae		01NH01					JX270336	KF017814		KF017699			KF017754	KF017645						
LL95	Llimoniella terricola	Cordieriticaeae	Cordieriticaeae		Diederich LD				KX090895		KX090842	KX090789	KX090741			KX090693							
Lorju1	Loramyces juncicola	Loramycetaceae	Loramycetaceae	Type species	ATCC 46458		Lorju1 (4, 30)		genome			genome	genome	genome		genome	genome	genome	genome	genome	genome	genome	genome
Lorma1	Loramyces macrosporus	Loramycetaceae	Loramycetaceae		CBS 235.53		Lorma1 (4, 30)	AFTOL-ID 913	DQ471005	NR_138379	DQ470957	genome	genome	genome	FJ190599	genome	genome	genome	genome	genome	genome	genome	genome
Marbr1	Marssonina brunnea	Drepanopezizaceae	Drepanopezizaceae				Marbr1 (4, 19)		genome	genome	genome	genome	genome	genome		genome	genome	genome	genome	genome	genome	genome	genome
R1033	Marthamyces cf. emarginatus	Marthamycetaceae	Marthamycetaceae	Type species	ICMP 17398 (8)				**MH682226**	**MH682227**	**HM140558**				HM143831								
R365	Marthamyces dracophylli	Marthamycetaceae	Marthamycetaceae		ICMP 17381 (8)					**MH682228**			**MH700584**		**MH698450**								
Melbi2	Meliniomyces bicolor	Hyaloscyphaceae	Hyaloscyphaceae				Melbi2 (4, 13)		genome	genome	genome	genome	genome	genome		genome	genome	genome	genome	genome	genome	genome	genome
Melva1	Meliniomyces variabilis	Hyaloscyphaceae	Hyaloscyphaceae	Type species			Melva1 (4, 13)		genome	genome		genome	genome	genome		genome	genome	genome	genome	genome	genome	genome	genome
AFTOL_ID_907	Meria laricis	Cenangiaceae	Cenangiaceae	Type species	CBS 298.52			CBS 298.52; ITS as AFTOL-ID 244, but the same CBS number	DQ471002	KT225534	DQ470954	DQ471146	DQ470904		FJ190598	DQ471073							
KL220	Microglossum olivaceum	Leotiaceae	Leotiaceae	Type species	TU 101778 (10)				KX090868	**MH752066**	KX090817	KX090764	KX090718			KX090669							
AFTOL_ID_1292	Microglossum rufum	Leotiaceae	Leotiaceae		OSC 100641				DQ471033		DQ470981	DQ471179	DQ470933			DQ471104							
F52489	Microscypha ellisii	Pezizellaceae	Pezizellaceae		KUS-F52489					JN033428	JN086731				JN086797								
F52303	Microscypha sp.	Pezizellaceae	Pezizellaceae		KUS-F52303					JN033403	JN086706		JN086857		JN086779								
M145	Mniaecia jungermanniae	Mniacea lineage	Mniaceaceae		TUR 168869				EU940036	EU940185	EU940109				EU940259								
M167	Mniaecia nivea	Mniacea lineage	Mniaceaceae		TUR 185325				EU940042	EU940188	EU940115				EU940264								
10544	Moellerodiscus lentus	Rutstroemiaceae	Rutstroemiaceae		HMAS 275557 (9)				**MH729334**	KU668566	**MH729337**	**MH729343**	**MH729344**										
M_pin_Hosoya	Moellerodiscus pinicola	Rutstroemiaceae	Rutstroemiaceae		TNS-F-40115 (7)				**LC434564**	AB926078	AB926162		AB926192		**LC431757**								
M_ame_Hosoya	'Mollisia' amenticola		Mollisiaceae		TNS-F-30411 (7)				**LC434586**	**LC425041**	**LC429379**												
AFTOL_ID_76	Mollisia cinerea	Mollisiaceae	Mollisiaceae	Type species	OSC 100029				DQ470990	DQ491498	DQ470942	DQ471122	DQ470883		DQ976372	DQ471051							
D365	'Mollisia' sp.		Drepanopezizaceae		ICMP 21729 (8)	**SAMN09907753** (3, 4)		Cadophora clade (Ploettnerulaceae)	genome	**MG195562**	genome	genome	genome	genome	genome	genome	genome	genome	genome	genome	genome	genome	genome
KUS_F52307	Mollisina uncinata	Pezizellaceae	Pezizellaceae		KUS-F52307					JN033404	JN086707		JN086858		JN086780								
CCM_F10289	Mycofalcella calcarata	Ascomycota incertae sedis (5)	Helotiaceae	exType specimen	CCM F-10289 (6)				**MK226447**	KC834065	KC834033	**MK241474**	**MK241439**			**MK241450**							
KKUK1	Myriodiscus sparassoides	Tympanidaceae	Tympanidaceae	Type species	KKUK1				JX219377	JX219379	JX219381												
LMK759	Myriosclerotinia curreyana	Sclerotiniaceae	Sclerotiniaceae		LMK759	SAMN03448624 (4)			genome	genome	genome	genome	genome	genome		genome	genome	genome	genome	genome	genome	genome	genome
LMK746	Myriosclerotinia duriaeana	Sclerotiniaceae	Sclerotiniaceae		LMK746	SAMN03448623				genome		genome	genome	genome	genome	genome	genome	genome	genome	genome	genome	genome	genome
LMK735	Myriosclerotinia scirpicola	Sclerotiniaceae	Sclerotiniaceae	Type species	LMK735	SAMN03448621				genome		genome	genome	genome	genome	genome	genome	genome	genome	genome	genome	genome	genome
AFTOL_ID_1295	Naemacyclus fimbriatus	Marthamycetaceae	Marthamycetaceae	Type species	CBS 289.61			N. fimbriatus = N. pinastri, the type species of Naemacyclus (DiCosmo et al. 1983, Eur. J. For. Path. 13: 206-212)	FJ176811		FJ176867					FJ238399							
D2031	Neobulgaria alba	Gelatinodiscaceae	Gelatinodiscaceae		ICMP 18395 (8)	**SAMN09907752** (3, 4)			HM116778	HM116779	HM116780	**MH682250**	genome	genome	genome	genome	genome	**MH700577**	genome	genome	genome	genome	genome
AFTOL_ID_1259	Neobulgaria pura	Gelatinodiscaceae	Gelatinodiscaceae	Type species	CBS 477.97						FJ176865	FJ238434	FJ238350			FJ238397							

Code	species	current higher taxon (1)	Accepted higher taxon	type status (2)	Voucher or strain	NCBI genome biosample	JGI genome	notes	SSU	ITS	LSU	RPB1	RPB2	RPA1	mtSSU	TEF1	Btub	MCM7	RPC2	TFB4	RPA2	a-tub	SF3B1
CBS102863	Neofabraea malicorticis	Dermateaceae	Dermateaceae	Type species	CBS 102863			epitype [#MBT201265]		NR 144926	KR858876		KR859325			KX982708	KR859290						
AFTOL ID 149	Neofabraea malicorticis	Dermateaceae	Dermateaceae		OSC 100041				AY544706		AY544662	DQ471124	DQ470885		AY544751	DQ847414							
OR74A	Neurospora crassa	OUTGROUP			OR74A		Neucr2 (4, 20)		X04971	XR_898134	AF286411	XM_959004	XM_324476			XM_959775							
Oidma1	Oidiodendron maius	Myxotricaceae (5)	Myxotrichaceae				Oidma1 (4, 21)		genome	genome	genome	genome	genome	genome		genome	genome	genome	genome	genome	genome	genome	genome
KACC45226	Olla millepunctata	Hyaloscyphaceae	Hyaloscyphaceae		KACC 45226					JN033380	JN086683		JN086835		JN086760								
CBS124810	Parafabraea eucalypti	Dermateaceae	Dermateaceae	exType specimen	CBS 124810					NR 132835	KR858882		KR859331			KX982731	KR866109						
AFTOL_ID_938	Pezicula carpinea	Dermateaceae	Dermateaceae		CBS 282.39				DQ471016		DQ470967	DQ842032	DQ479934		FJ190608	DQ479932							
CBS 923 96	Pezicula carpinea	Dermateaceae	Dermateaceae	exType specimen	CBS 923.96			epitype [#MBT201267]		NR 144927	KR858899		KF376158			KF376219	KF376279						
NRRL12192	Pezicula radicicola	Dermateaceae	Dermateaceae		NRRL 12192	SAMN07709029 (4)						genome	genome	genome		genome	genome	genome	genome	genome	genome	genome	genome
D852	Pezizellaceae sp.		Helotiales (Han Clade 4)		ICMP 22537 (8)					**MH682232**		**MH682253**						**MH700574**					
D2518	Pezizellaceae sp.		Pezizellaceae		PDD 111530				**MH985298**	**MH578481**	**MH985295**	**MH986708**	**MH986704**					**MH986711**					
E17	Pezoloma cf. websteri	Discinella-Pezoloma lineage	Discinella-Pezoloma lineage		FH (J. Karakehian 13051805)				**MH754756**	**MH754757**	**MH754758**		**MK244998**										
M283	Pezoloma ciliifera	Discinella-Pezoloma lineage	Discinella-Pezoloma lineage		TUR 174364				EU940068	EU940219	EU940143				EU940283								
AFTOL ID 1253	Phacidium lacerum	Phacidiaceae	Phacidiaceae		CBS 130.30				DQ471028	KJ663841	DQ470976	DQ471174	KJ663923		FJ190623	FJ238396							
P_epi_Hosoya	Phaeohelotium epiphyllum	Helotiaceae	Helotiaceae		TNS-F-40042 (7)				**LC434558**	AB926061	AB926130		AB926219		**LC431759**								
KUS_F52576	Phialina lachnobrachyoides	Pezizellaceae	Pezizellaceae		KUS-F52576					JN033424	JN086727		JN086868										
CBS 300 62	Phialocephala dimorphospora	Mollisiaceae	Mollisiaceae	exType specimen	CBS 300.62			epitype (27)		NR_135931	AB671465	KP965549											
Phisc1	'Phialocephala' scopiformis		Mollisiaceae		CBS 120377		Phisc1 (4, 22)		genome	genome	genome	genome	genome	genome		genome	genome	genome	genome	genome	genome	genome	genome
D728	Phialocephala sp.	Mollisiaceae	Mollisiaceae		ICMP 21725 (8)	**SAMN09907756** (3, 4)		culture from ascospores	genome	**MG195462**	genome	**MH682249**	genome	genome	genome	genome	genome	**MH700572**	genome	genome	genome	genome	genome
UAMH11012	'Phialocephala' subalpina		Mollisiaceae		UAMH 11012	SAMEA3883069 (4)			genome	genome	genome	genome	genome	genome	genome	genome	genome	genome	genome	genome	genome	genome	genome
CBS109875	Phlyctema vagabunda	Dermateaceae	Dermateaceae	Type species	CBS 109875					KR859275	KR859069		KR859346			KX982718	KR866088						
KL98	Piceomphale bulgarioides	Sclerotiniaceae	Sclerotiniaceae	Type species	TAAM 165289				KX090848	LT158483	KX090797		KX090700										
R_bulg_Hosoya	Piceomphale bulgarioides	Sclerotiniaceae	Rutstroemiaceae		TNS-F-40005 (7)			as Rutstroemia bulgarioides	**LC434556**	AB926053	AB926122		AB926176										
BPI843555	Pilidium acerinum	Chaetomellaceae	Chaetomellaceae	exType specimen	CBS 736.68				AY487093	NR_119500	AY487092												
D792	'Pirottaea' palmicola		Helotiaceae		ICMP 13383 (8)	**SAMN09907758** (3, 4)			genome	KM677205	genome	genome	genome	genome	genome	genome	genome	genome	genome	genome	genome	genome	genome
D809	'Pirottaea' palmicola		Helotiaceae		ICMP 13384 (8)					KM677207		**MH682246**						**MH700569**					
CBS_345_73	Pleuroascus nicholsonii	Pseudeurotiaceae	Helotiaceae	exType specimen	CBS 345.73			neotype (24)	AF096182.	KJ755519	AF096196												
P_pseud_Hosoya	Poculum pseudosydowianum	Rutstroemiaceae	Rutstroemiaceae		TNS-F-40071 (7)				**LC434561**	AB904505	AB926136		AB926182		**LC431754**		**MWLR Datastore**						
TNS F12764	Polydesmia pruinosa	incertae sedis	Helotiales (sclerotinioid clade)	Type species	TNS-F-12764 (7)				**LC434574**	JN033453	JN086753	**LC431672**	JN086899										
REF050	Polyphilus frankenii	Hyaloscyphaceae (5)	Helotiales (Stamnaria lineage/Han Clade 9)		REF050				MG719730	JN859270	MG719709		MG719751										
REF052	Polyphilus sieberi	Hyaloscyphaceae (5)	Helotiales (Stamnaria lineage/Han Clade 9)	exType specimen	REF052				MG719712	JN859272	MG719692		MG719733										
AFTOL_ID_744	Potebniamyces pyri	Phacidiaceae	Phacidiaceae	Type species	S001			type species P. discolor = P. pyri	DQ470997	DQ491510	DQ470949	DQ471142	DQ470900			DQ471068							
D683	Proliferodiscus dingleyae	Lachnaceae	Lachnaceae		ICMP 21730 (8)	**SAMN09907755** (3, 4)			genome	**MH682231**	genome	genome	genome	genome	genome	genome	genome	genome	genome	genome	genome	genome	genome
R918	Propolis farinosus	Marthamycetaceae	Marthamycetaceae		ICMP 17354 (8)				**MH682223**	**MH682229**	HM140562		**MH700582**		**MH698451**								
ILLS_60497	Propolis versicolor	Marthamycetaceae	Marthamycetaceae		ILLS 60497					JQ256425	JN012015							JQ256451					
AFTOL ID 1912	Pseudeurotium zonatum	Pseudeurotiaceae	Pseudeurotiaceae	exType specimen	CBS 329.36				DQ471040	NR 111127	DQ470988	DQ471186	DQ470940		FJ190655	DQ471112							
CBS 130532	Pseudofabraea citricarpa	Dermateaceae	Dermateaceae	exType specimen	CBS 130532					KR859280	KR859074		KR859351				KR859314						
Pseel	Pseudographis elatina	Triblidiaceae	Rhytismataceae	Type species	CBS 651.97		Pseel1 (25)		genome	genome		genome	genome	genome	genome	genome	genome	genome	genome	genome	genome	genome	genome
Pseudest	Pseudogymnoascus destructans	Pseudeurotiaceae	Pseudeurotiaceae		20631-21		Pseudest1 (4, 23)		genome	genome	genome	genome	genome	genome		genome	genome	genome	genome	genome	genome	genome	genome
UAMH1990	Pseudogymnoascus roseus	Pseudeurotiaceae	Pseudeurotiaceae	Type species	UAMH 1990					KJ755524		KJ755469	KJ755495										
D2527	'Pseudopeziza' colensoi		Cenangiaceae		PDD 112240					**MH921874**	**MH985297**	**MH986706**	**MH986705**					**MH986709**					
F52105	Psilachnum staphyleae	Pezizellaceae	Helotiales (Stamnaria lineage/Han Clade 9)		KUS F52105					JN033396	JN086699		JN086850		JN086773								
P_pro_Hosoya	'Pyrenopeziza' protrusa		Mollisiaceae		TNS-F-23154 (7)				**LC434587**	**LC426322**	**LC429375**	**LC431683**	**LC431722**										
Rhesp1	Rhexocercosporidium sp.	Ploettnerulaceae	Ploettnerulaceae		MPI-PUGE-AT-0058		Rhesp1 (4, 12)			genome JGI	genome JGI	genome JGI	genome JGI	genome		genome JGI	genome JGI	genome JGI	genome	genome	genome	genome	genome
CBS 110605	Rhizodermea veluwensis	Dermateaceae	Dermateaceae	exType specimen	CBS 110605					NR 137757	KR859076		KR859353				KR859316						
Rhier1	Rhizoscyphus ericae	Hyaloscyphaceae	Hyaloscyphaceae	Type species	UAMH 7357		Rhier1 (4, 13)					genome	genome	genome		genome	genome	genome	genome	genome	genome	genome	genome
Ertz16200	Rhymbocarpus fuscoatrae	Cordieriticaceae	Cordieriticaceae		BR (Ertz 16200)				KJ559593		KJ559571												
04CH_RAC_A_6_1	Rhynchosporium agropyri	Ploettnerulaceae	Ploettnerulaceae		04CH-RAC-A.6.1	SAMEA3895814						genome	genome	genome	genome	genome	genome	genome	genome	genome	genome	genome	genome
UK7	Rhynchosporium commune	Ploettnerulaceae	Ploettnerulaceae	Type species	UK7	SAMEA3895816 (4)		R. commune = type species R. graminicola fide Species Fungorum				genome	genome	genome	genome	genome	genome	genome	genome	genome	genome	genome	genome
02CH4_6a_1	Rhynchosporium secalis	Ploettnerulaceae	Ploettnerulaceae		02CH4-6a.1	SAMEA3895815						genome	genome	genome	genome	genome	genome	genome	genome	genome	genome	AJ006211	genome
F52443	'Rodwayella' citrinula		Helotiales (Stamnaria lineage/Han Clade 9)		KUS-F52443					JN033414	JN086717		JN086862		JN086787								
R sub Hosoya	Roesleria subterranea	Roesleriaceae	Helotiaceae	Type species	TNS-F-38701 (7)				**LC434568**	AB628057	AB628056	**LC431682**	**LC431717**		**LC431764**								
HB9951c	Rommelaarsia flavovirens	Helotiales incertae sedis (5)	Helotiales (Stamnaria lineage/Han Clade 9)	exType specimen	H.B. 9951				**MH712452**	KT958774	KT958771	**MK244985**											
DH267	'Roseodiscus' formosus		incertae sedis Helotiales		FH (SBRH 686) (11)					KT972711	KT972712	**MK244981**	**MK244997**			**MK244991**							
DH257	Roseodiscus rhodoleucus	incertae sedis	Helotiales (Stamnaria lineage/Han Clade 9)	Type species	HB 8488a (11)				KT972703	KT972704	KT972705	**MK244979**				**MK244990**		**MK244986**					
CBS111548	Rutstroemia echinophila	Rutstroemiaceae	Rutstroemiaceae		CBS 111548	SAMN02903859 (4)		as Lanzia echinophila	KF545541	KF545321		genome	genome	genome	genome	genome	KF545196	genome	genome		genome	genome	genome
Rutfi1	Rutstroemia firma	Rutstroemiaceae	Rutstroemiaceae	Type species	CBS 115.86		Rutfi1 (4, 30)		genome	genome	genome	genome	genome	genome		KF545499	KF545228	KF545462	genome	genome	genome	genome	genome
AFTOL_ID_923	Rutstroemia firma	Rutstroemiaceae	Rutstroemiaceae	Type species	CBS 341.62				DQ471010	KF545334	DQ470963	DQ471155	DQ470912			DQ471082	KF545205	KF545461					
CBS115975	Rutstroemia sydowiana	Rutstroemiaceae	Rutstroemiaceae		CBS 115975	SAMN02903521 (4)			genome	KF545331	genome	genome	genome	genome	genome	genome	genome	genome	genome	genome	genome	genome	genome
KL160	Rutstroemia tiliacea	Rutstroemiaceae	Rutstroemiaceae		TAAM 132844			specimen HB 6734	KX090860	LT158423	KX090808	KX090757	KX090711			KX090661							
Sclsc	Sclerotinia sclerotiorum	Sclerotiniaceae	Sclerotiniaceae	Type species	ATCC 18683		Sclsc1 (4, 15)		genome	genome	genome	genome	genome	genome		genome	genome	genome	genome	genome	genome	genome	genome
S_scl_Hosoya	Sclerotinia sclerotiorum	Sclerotiniaceae	Sclerotiniaceae	Type species	TNS-F-40021 (7)				**LC434555**	AB926054	**LC429380**		**LC431713**				**MWLR Datastore**						
IHIA52	Scytalidium lignicola	Leotiomycetes incertae sedis (5)	Helotiales incertae sedis		DSM 105466	SAMN06704609			genome	genome	genome	genome	genome	genome	genome	genome	genome	genome	genome	genome	genome	genome	genome
Spafl1	Spathularia flavida	Cudoniaceae	Cudoniaceae	Type species			Spafl1 (25)		genome	genome	genome	genome	genome	genome	genome	genome	genome	genome	genome	genome	genome	genome	genome
AFTOL_ID_1291	Spathularia velutipes	Cudoniaceae	Cudoniaceae		OSC 100640				FJ997860		FJ997861		FJ997863			FJ997862							
NBRC108774	Stamnaria americana	Stamnaria lineage	Helotiales (Stamnaria lineage/Han Clade 9)		NBRC 108774				AB773855		AB773854	AB773856	AB773857			AB773858							
940	Stamnaria austriaca	Stamnaria lineage	Helotiales (Stamnaria lineage/Han Clade 9)		GZU (Gruber 151/225) (11)				MH712450	KT972708	KT972709	**MK244983**	**MK244996**			**MK244993**		**MK244989**					
S bak Hosoya	Strossmayeria bakeriana	Strossmayeria lineage	Strossmayeria lineage		TNS-F-11199 (7)				**LC434572**	**LC425043**	**LC429373**	**LC431671**	**LC431714**		**LC431761**								
T_fusc_Hosoya	Tapesia fusca	Mollisiaceae	Mollisiaceae	Type species	TNS-F-17463 (7)				**LC434579**	**LC425049**	**LC429378**	**LC431675**	**LC431721**										
R912	Terriera minus	Rhytismataceae	Rhytismataceae		ICMP 13974 (8)				**MH682225**	AY100665	HM140571				HM143843								
CCM F26199	Tetracladium marchalianum	incertae sedis	Helotiales (Stamnaria lineage/Han Clade 9)	Type species	CCM F-26199 (6)				AY204614	AY204621	AY204612	**MK241481**	**MK241443**			**MK241455**	**MK241466**						
AFTOL ID 5005	Thelebolus ellipsoideus	Thelebolaceae	Thelebolaceae		CBS 113937				FJ176840		FJ176895	FJ238445	FJ238378		FJ190657								
AFTOL ID 5016	Thelebolus globosus	Thelebolaceae	Thelebolaceae		CBS 113940				FJ176851		FJ176905	FJ238446	FJ238385		FJ190662	FJ238418							
Themi	Thelebolus microsporus	Thelebolaceae	Thelebolaceae		ATCC 90970		Themi1 (4, 30)					genome	genome	genome		genome	genome	genome	genome		genome	genome	genome
Thest1	Thelebolus stercoreus	Thelebolaceae	Thelebolaceae	Type species			Thest1 (25)		genome	genome	genome	genome	genome	genome	genome	genome	genome	genome	genome		genome	genome	genome
ILLS61044	Thuemenidium atropurpureum	Leotiaceae	Leotiaceae		ILLS 61044					JQ256427	JQ256441	KC222176											
T mol Hosoya	Trichopeziza mollisima	Lachnaceae	Lachnaceae		TNS-F-16760 (7)				**LC434567**	**AB481285**	**LC429374**	**LC431681**	**AB481360**				**MWLR Datastore**						
KUS_F52218	Trichopeziza sulphurea	Lachnaceae	Lachnaceae	Type species	KUS-F52218					JN033398	JN086701		JN086852		JN086775								
T ota Hosoya	Trichopezizella otanii	Lachnaceae	Lachnaceae		FC-2156, TNS-F-16579 (7)				**LC434590**	AB481287	AB481307	**LC431685**	AB481345				**MWLR Datastore**						
CCM F14598	Tricladium obesum	Helotiaceae	Helotiaceae		CCM F-14598 (6)				**MK235119**	KC834068	KC834035	**MK241475**	**MK241440**			**MK241452**	**MK241464**						
CCM F16599	Tricladium splendens	Helotiaceae	Helotiaceae	Type species	CCM F-16599 (6)				AY204631	AY204635	GQ477333	**MK241476**	**MK241441**			**MK241451**	**MK241465**						
M160	Trizodia acrobia	Mniacea lineage	Mniaceaceae	type_species	TUR 165562				EU940041	EU940191	EU940114		EU940327		EU940263								
KL332	Trochila craterium	Cenangiaceae	Cenangiaceae	Type species	BPI 880163				KX090886			KX090779											
KL336	Trochila laurocerasi	Cenangiaceae	Cenangiaceae		BPI 879818				KX090887	LT158460	KX090835	KX090780	KX090734			KX090689							
AFTOL ID 1319	Tryblidiopsis pinastri	Rhytismataceae	Rhytismataceae	Type species	CBS 445.71				DQ471035	JF793678	DQ470983	DQ471181	DQ470935		AF431963	DQ471106							
LQH8	Tympanis confusa	Tympanidaceae	Tympanidaceae		F (Quijada LQH8) (31)					**MH810146**	**MH810148**		**MH818601**										
AA1	Tympanis tsugae	Tympanidaceae	Tympanidaceae		F (Quijada AA1) (31)					**MH810147**	**MH810149**					**MH818600**							
F18014	'Urceolella' carestiana		Helotiales (Stamnaria lineage/Han Clade 9)		TNS-F-18014					JN033443	JN086744		JN086888		JN086813								
CBS 608 77	Urceolella crispula	Hyaloscyphaceae	Helotiales (Stamnaria lineage/Han Clade 9)	type species	CBS 608.77			=KACC45486		JN033379	JN086682				JN086759								
JW1033	Vandijckella johannae	Vandijckellaceae (5)	Vandijckellaceae	exType specimen	CBS 143182					LT904725	LT904726		LT904707				LT904703						
CCM_F19494	'Varicosporium' delicatum		incertae sedis (mollisioid clade)		CBS 574.95 (6)				**MK226446**	JQ412864	KC834036	**MK241478**	**MK241442**			**MK241457**	**MK241463**						
CCM F04276	Varicosporium elodeae	Helotiaceae (5)	Discinella-Pezoloma lineage	Type species	CCM F-04276 (6)				AY425613	**MK226459**	KC834037	**MK241470**	**MK241435**			**MK241446**	**MK241459**						
TNS F18030	Venturiocistella japonica	Hyaloscyphaceae	Helotiales (Han Clade 4)		TNS-F-18030 (7)					JN033447	AB546954	**LC431669**	JN086893		JN086818								
AFTOL_ID_1322	Vibrissea truncorum	Vibrisseaceae	Vibrisseaceae	Type species	CBS 258.91				FJ176818	EU434854	FJ176874	FJ238438	FJ238356		FJ190635	FJ238405							
AR13	Xerombrophila crystallifera	Gelatinodiscaceae	Gelatinodiscaceae	Type species	TAAM 122762 (10)				**MH758755**	**MH752071**	**MH748085**												
KL251	Xeropilidium dennisii	Chaetomellaceae	Chaetomellaceae	exType specimen	TU 104501				KX090876	LT158441	KX090824	KX090770	KX090723			KX090677							
AFTOL ID 51	Xylaria hypoxylon	OUTGROUP			OSC 100004		Xylhyp1 (4, 25)		AY544692	DQ491487	AY544648	DQ471114	DQ470878			DQ471042							

(1) based on Baral 2016 unless otherwise noted
(2) ExType specimen = type specimen of the type species of the genus; type species = specimens identified as the type species of the genus
(3) newly released Manaaki Whenua genome
(4) genome included in genome phylogeny
(5) Index Fungorum taxonomy
(6) new sequences generated by Chistiane Baschien, Leibniz-Institute DSMZ, German Collection of Microorganisms and Cell Cultures, Inhoffenstraße 7B, 38124 Braunschweig, Germany.
(7) new sequences generated by Tsuyoshi Hosoya, National Museum of Nature and Science, Dept. of Botany; Amakubo 4-1-1, Tsukuba, Ibaraki 305-0005, Japan.
(8) new sequences generated by Duckchul Park, Manaaki Whenua Landcare Research, Private Bag 92170, Auckland, New Zealand.
(9) new sequences generated by Wen-Ying Zhuang, Chinese Academy of Sciences, State Key Laboratory of Mycology, Institute of Microbiology, Beijing 100101, China.
(10) new sequences generated by Kadri Pärtel, Institute of Ecology and Earth Sciences, University of Tartu, Lai 40, Tartu EE-51005, Estonia.
(11) new sequences generated by Danny Haelewaters, Organismic and Evolutionary Biology, Harvard University, 22 Divinity Avenue, Cambridge, MA 02138, USA.
(12) Genome provided by F. Martin, the Mycorrhizal Genomics Initiative and the 1000 Fungal Genomes consortium
(13) Martino E, et al. (2018) Comparative genomics and transcriptomics depict ericoid mycorrhizal fungi as versatile saprotrophs and plant mutualists. New Phytol. 217: 1213-1229. doi: 10.1111/nph.14974.
(14) Frantzeskakis L, et al. (2018) Signatures of host specialization and a recent transposable element burst in the dynamic one-speed genome of the fungal barley powdery mildew pathogen. BMC Genomics 19: 381. doi: 10.1186/s12864-018-4750-6.
(15) Amselem J, et al. (2011) Genomic analysis of the necrotrophic fungal pathogens Sclerotinia sclerotiorum and Botrytis cinerea. PLoS Genet. 7(8):e1002230. doi: 10.1371/journal.pgen.1002230.
(16) Knapp DG, et al. (2018) Comparative genomics provides insights into the lifestyle and reveals functional heterogeneity of dark septate endophytic fungi. Sci Rep. 8: 6321. doi: 10.1038/s41598-018-24686-4.
(17) Jones L, et al. (2014) Adaptive genomic structural variation in the grape powdery mildew pathogen, Erysiphe necator. BMC Genomics 15:1081. doi: 10.1186/1471-2164-15-1081.
(18) Chen L, et al. (2013) Genomics-driven discovery of the pneumocandin biosynthetic gene cluster in the fungus Glarea lozoyensis. BMC Genomics 14: 339. doi: 10.1186/1471-2164-14-339.
(19) Zhu S, et al. (2012) Sequencing the genome of Marssonina brunnea reveals fungus-poplar co-evolution. BMC Genomics 13: 382. doi: 10.1186/1471-2164-13-382.
(20) Galagan JE, et al. (2003) The genome sequence of the filamentous fungus Neurospora crassa. Nature 422:859-68. doi: 10.1038/nature01554.
(21) Kohler A, et al. (2015) Convergent losses of decay mechanisms and rapid turnover of symbiosis genes in mycorrhizal mutualists. Nat Genet. 47:410-415. doi: 10.1038/ng.3223.
(22) Walker AK, et al. (2016) Full Genome of Phialocephala scopiformis DAOMC 229536, a Fungal Endophyte of Spruce Producing the Potent Anti-Insectan Compound Rugulosin. Genome Announc. 4(1). pii: 4/1/e01768-15. doi: 10.1128/genomeA.01768-15.
(23) Drees KP, et al. (2016) Use of Multiple Sequencing Technologies To Produce a High-Quality Genome of the Fungus Pseudogymnoascus destructans, the Causative Agent of Bat White-Nose Syndrome. Genome Announc. 4(3). pii: 4/3/e00445-16. doi: 10.1128/genomeA.00445-16.
(24) Suh S-O, Blackwell M (1999) Molecular Phylogeny of the Cleistothecial Fungi Placed in Cephalothecaceae and Pseudeurotiaceae. Mycologia 91: 836-848.
(25) Genome provided by Joseph Sapatfora and collaborators through the Assembling the Fungal Tree of Life project, funded by NSF.
(26) Genome provided by Mary Wildermuth and Shauna Somerville, University of California Berkely through the JGI CSP #1657 Comparative Genomics of Powdery Mildews and Associated Host Plants project, using biological material provided by K. Scheibel and Dr. S. Somerville (University of California, Berkeley).
(27) Tanney JB, Douglas B, Seifert KA (2016) Sexual and asexual states of some endophytic Phialocephala species of Picea. Mycologia 108: 255–280. DOI: 10.3852/15-136
(28) Gianoulis TA, Griffin MA, Spakowicz DJ, Dunican BF, Alpha CJ, Sboner A, et al. (2012) Genomic Analysis of the Hydrocarbon-Producing, Cellulolytic, Endophytic Fungus Ascocoryne sarcoides. PLoS Genet 8(3): e1002558.
(29) Genome provided by Richard Hamelin, through work conducted by the U.S. Department of Energy Joint Genome Institute, a DOE Office of Science User Facility, supported by the Office of Science of the U.S. Department of Energy under Contract No. DE-AC02-05CH11231.
(30) Genome provided by Jon Magnuson, Pacific Northwest National Laboratory, USA, through the 1000 Fungal Genomes project with supported of the Office of Science of the U.S. Department of Energy under Contract No. DE-AC02-05CH11231.
(31) new sequences generated by Luis Quijada, Organismic and Evolutionary Biology, Harvard University, 22 Divinity Avenue, Cambridge, MA 02138, USA.
