# Supplementary material for: A multigene phylogeny toward a new phylogenetic classification of Leotiomycetes
Source: IMA Fungus. 2019 Jun 7;10:1. doi: 10.1186/s43008-019-0002-x (PMC7325659; doi:10.1186/s43008-019-0002-x)
Supplement: Supplementary file 2 — Table S2. Sequences used in ITS phylogeny, Additional file 6: Figure S2. Accepted family based on ITS and 15-gene analyses in this paper. (PDF 761 kb) [file 43008_2019_2_MOESM2_ESM.pdf]

Suppl. Data Table S2. Sequences used in ITS phylogeny, Suppl. Data Fig 2. Accepted family based on ITS and 15-gene analyses in this paper.
(1) Family from Baral (2016) unless otherwise stated
(2) Family from Index Fungorum where not treated by Baral (2016)
(3) ExType represents a type specimen; TypeSpecies a specimen that has been identified as the type species of the genus

Genbank accession or code	Species	Voucher	Type status of specimen (3)	Family (1, 2)	Accepted family	Notes
NR_119482	Acephala applanata	CBS 109321	exTypeSpecimen	Mollisiaceae	Mollisiaceae	
Acema1	Acephala macrosclerotiorum	CBS 123555		Mollisiaceae	Mollisiaceae	ITS extracted from JGI genome
JX124323	Acidea extrema	MH-2012 strain 1180	exTypeSpecimen	Helotiales incertae sedis (2)	Discinella-Pezoloma lineage	
KF874619	Acidomelania panicicola	61R8	exTypeSpecimen	Mollisiaceae	Mollisiaceae	
AY204588	Alatospora acuminata	CCM F-12186	TypeSpecies	Leotiaceae (2)	Leotiales incertae sedis	neotype
DSM104360	Alatospora acuminata	DSM 104360	TypeSpecies	Leotiaceae (2)	Leotiales incertae sedis	GenBank AY204588
KT005452	Albotricha acutipila	D40d	Type Species	Lachnaceae	Lachnaceae	
AB481234	Albotricha acutipila	FC-2262, TNS-F-16740	TypeSpecies	Lachnaceae	Lachnaceae	
AB481235	Albotricha albotestacea	FC-2094, TNS-F-16497		Lachnaceae	Lachnaceae	
NR_157437	Allantophomopsiella pseudotsugae	CBS 320.53	exTypeSpecimen	Phacidiaceae	Phacidiaceae	
KJ663828	Allantophomopsiella pseudotsugae	CBS 562.63	TypeSpecies	Phacidiaceae	Phacidiaceae	
NR_132922	Allantophomopsis lunata	CBS 137781	exTypeSpecimen	Phacidiaceae	Phacidiaceae	epitype
KL391	Ameghiniella australis	FH 01146515	TypeSpecies	Cordieriticaeae	Cordieriticaeae	GenBank MH752070
JN033411	Amicodisca castaneae	KUS F51917		Hyaloscyphaceae	Hyaloscyphaceae	
JN033389	Amicodisca sp	KUS F51377		Hyaloscyphaceae	Hyaloscyphaceae	
MH221521	Amicodisca virella	SBRH 828	TypeSpecies	Hyaloscyphaceae	Hyaloscyphaceae	
NR_119450	Amorphotheca resinae	ATCC 200942	exTypeSpecimen	Amorphothecaceae	Amorphothecaceae	
EU030278	Amorphotheca resinae	ATCC 22711		Amorphothecaceae	Amorphothecaceae	JGI genome also available for this specimen
KM272369	Amylocarpus encephaloides	017cN	TypeSpecies	Helotiales incertae sedis	Helotiaceae	
AJ133431	Antarctomyces psychrotrophicus	IMI 378528	exTypeSpecimen	Pezizellaceae	Thelebolaceae	
NR_111345	Aquapoterium pinicola	ATCC MYA-4213	exTypeSpecimen	Helotiales incertae sedis (2)	Helotiales incertae sedis	
MH578553	Arachnopeziza araneosa	ICMP 21731		Arachnopezizaceae	Arachnopezizaceae	MWLR genome also available for this specimen
JN033436	Arachnopeziza aurata	TNS-F-11212		Arachnopezizaceae	Arachnopezizaceae	
A_aurel_Hosoya	Arachnopeziza aurelia	TNS-F-40009	TypeSpecies	Arachnopezizaceae	Arachnopezizaceae	GenBank LC425050
JN033445	Arachnopeziza obtusipila	TNS-F-12768		Arachnopezizaceae	Arachnopezizaceae	
MH578549	Arachnopeziza sp on Dianella	ICMP 22831		Arachnopezizaceae	Arachnopezizaceae	
KC834042	Arbusculina fragmentans	CCM F-13486		Pezizomycotina incertae sedis (2)	Hyaloscyphaceae	
KP234351	'Articulospora' proliferata	CCM F-11200				
DSM_104345	Articulospora tetracladia	DSM 104345	TypeSpecies	Discinella-Pezoloma lineage	Discinella-Pezoloma lineage	GenBank MK226461
AF260815	Ascocalyx abietina	ATCC 28379		Godroniaceae	Godroniaceae	
FJ746661	Ascocalyx abietina	ATCC 34573		Godroniaceae	Godroniaceae	
JN033406	Ascocoryne cylichnium	KUS F52351		Gelatinodiscaceae	Gelatinodiscaceae	
HM152546	Ascocoryne sarcoides	CBS 171.56	TypeSpecies	Gelatinodiscaceae	Gelatinodiscaceae	
Ascsa1	Ascocoryne sarcoides	NRRL 50072	TypeSpecies	Gelatinodiscaceae	Gelatinodiscaceae	ITS extracted from JGI genome
MH682237	Ascocoryne sp	ICMP 22839		Gelatinodiscaceae	Gelatinodiscaceae	
KY462798	Ascotremella faginea	GM20151028	TypeSpecies	Gelatinodiscaceae	Gelatinodiscaceae	
KP161274	Asperopilum juncicola	PDD 56797	Type Species	Hyaloscyphaceae	Lachnaceae	
MH578456	Asterocalyx cf mirabilis	PDD 62628		Helotiales incertae sedis	Helotiales incertae sedis	
MH578480	Austropezia samuelsii	ICMP 22791	TypeSpecies	Arachnopezizaceae	Pezizellaceae	
KM880187	Banksiamyces sp.	PDD 105253		Helotiales incertae sedis	Helotiales incertae sedis	
KT598374	Barrenia panicia	RUTPP-WSF1R37	exTypeSpecimen	Helotiales incertae sedis (2)	Mollisiaceae	
KU900903	Belonioscyphella hypnorum	Bel2 Type	TypeSpecies	Helotiales incertae sedis	Stamnaria lineage/Han Clade 9	
KF499363	Bicornispora exophiala	AH 15779	exTypeSpecimen	Rutstroemiaceae	Rutstroemiaceae	
KF499361	Bicornispora seditiosa	WU 32445		Rutstroemiaceae	Rutstroemiaceae	
KY462800	Bispora antennata	G.M. 2014-09		Helotiaceae	Helotiaceae	anamorph of Bisporella sensu stricto
D2517	Bivallum zelandicum	PDD 112248		Rhytismataceae	Rhytismataceae	GenBank MH921846
KY853426	Bloxamia truncata	FMR 11240	TypeSpecies	Pezizellaceae	Pezizellaceae	Bloxamiaceae (Hernández-Restrepo et al. 2017)
KY653161	Blumeria graminis	OE2013PM31		Erysiphaceae	Erysiphaceae	
DH_14	Blumeria graminis hordei	DH-14	TypeSpecies	Erysiphaceae	Erysiphaceae	ITS extracted from JGI genome
KY929501	Blumeriella kerriae	JS20160615		Drepanopezizaceae	Drepanopezizaceae	
KX858917	Botrytis cinerea	1810	TypeSpecies	Sclerotiniaceae	Sclerotiniaceae	
KF727423	Brunaudia phormigena	PDD 75309	Type Species		Helotiaceae	

Genbank accession or code	Species	Voucher	Type status of specimen (3)	Family (1, 2)	Accepted family	Notes
JN033392	Brunnipila fuscescens	KUS F52031		Lachnaceae	Lachnaceae	
JX393084	Bryoclaviculus campylopi	PDD 101074	exTypeSpecimen	Bryoglossum lineage	Bryoglossum lineage	
AY789285	Bryoglossum gracile	DAOM 178087	Type Species	Bryoglossum lineage	Bryoglossum lineage	
KJ663831	Bulgaria inquinans	CBS 118.31	TypeSpecies	Phacidiaceae	Phacidiaceae	
MH578505	Bulgariella pulla	ICMP 22812	TypeSpecies	Helotiales incertae sedis	Helotiaceae	
KU845537	Bulgariella pulla	TL2011	TypeSpecies	Helotiales incertae sedis	Helotiaceae	
NR_111040	Byssoascus striatosporus	UAMH 3572	exTypeSpecimen	Myxotrichaceae	Myxotrichaceae	
AY249073	Cadophora fastigiata	CBS 307.49	TypeSpecies	Ploettnerulaceae	Ploettnerulaceae	
PRJEB13389	Cadophora malorum	Mo12		Ploettnerulaceae	Ploettnerulaceae	ITS extracted from NCBI genome
Cadsp1	Cadophora sp.	DSE1049		Ploettnerulaceae	Ploettnerulaceae	ITS extracted from JGI genome
KF836358	Cairneyella variabilis	VPRI 42388	exTypeSpecimen	incertae sedis	Helotiales hyaloscyphoid clade	
KT185667	Calloria urticae	G.M. 2015-04-12 5		Calloriaceae	Stamnaria lineage/Han Clade 9	
JN033382	Calycellina populina	CBS 247.62		Pezizellaceae	Pezizellaceae	
D1971	Calycellina sp.	ICMP 22839		Pezizellaceae	Pezizellaceae	GenBank MH921847; ex Knightia leaves
MH682235	Calycina citrina	ICMP 22830		Pezizellaceae	Pezizellaceae	
LC169492	Calycina discedens	TNS-F-37025		Pezizellaceae	Pezizellaceae	
JN033407	Calycina herbarum	KUS F52362	TypeSpecies	Pezizellaceae	Pezizellaceae	
KT185677	Calycina marina	TROM F26093		Pezizellaceae	Pezizellaceae	
Bisps1	Calycina sp.	PMI 857		Pezizellaceae	Pezizellaceae	as Bisporella sp.; ITS extracted from JGI genome
LT904864	Capitotricha bicolor	PRM 915564	Type Species	Lachnaceae	Lachnaceae	
KF033116	Cashiella sticheri	PDD 103198		Helotiales incertae sedis	Helotiales incertae sedis	
GU727557	Catenulifera brachyconia	CBS 700.73		Helotiales incertae sedis	Helotiales - Han Clade 4	
GU727561	Catenulifera brevicollaris	CBS 126.74		Helotiales incertae sedis	Helotiales - Han Clade 4	
GU727560	Catenulifera luxurians	CBS 647.75		Helotiales incertae sedis	Helotiales - Han Clade 4	
LT158425	Cenangiopsis quercicola	TAAM 178677	TypeSpecies	Cenangiaceae	Cenangiaceae	
KX090899	Cenangiopsis sp	Dgf/C7D-02-07-14		Cenangiaceae	Cenangiaceae	
LT158439	'Cenangium' acuum	TAAM 198515				
LT158444	Cenangium ferruginosum	OULU24432		Cenangiaceae	Cenangiaceae	
LT158471	Cenangium ferruginosum	TAAM 198451	TypeSpecies	Cenangiaceae	Cenangiaceae	
LT158458	Cenangium japonicum	CUP JA3745		Cenangiaceae	Cenangiaceae	
AY487082	Chaetomella oblonga	BPI 843553	TypeSpecies	Chaetomellaceae	Chaetomellaceae	
AY487085	Chaetomella raphigera	BPI 843551	TypeSpecies	Chaetomellaceae	Chaetomellaceae	
NR_145313	'Chalara' clidemiae	CBS 141319		Porodiplodiaceae (2)	Pezizellaceae	
Chalo1	Chalara longipes	JGIGenome			Hyaloscyphaceae	ITS extracted from JGI genome
KR611871	Cheirospora botryospora	CPC 24605	Type species	Ascomycota incertae sedis (2)	Mollisiaceae	
MH682234	Chlorencoelia torta	ICMP 22831		Cenangiaceae	Cenangiaceae	MWLR genome also available for this specimen
LT158479	Chlorencoelia versiformis	TU 119720	Type Species	Cenangiaceae	Cenangiaceae	
C_aerugascens_Hosoya	Chlorociboria aeruginascens	TNS-F-36241		Chlorociboriaceae	Chlorociboriaceae	Genbank LC425045
KL247	Chlorociboria aeruginella	TAAM 198514		Chlorociboriaceae	Chlorociboriaceae	GenBank MH752067
Z81426	Chlorociboria aeruginosa	Holst-Jensen 1785.P	Type Species	Chlorociboriaceae	Chlorociboriaceae	
DQ491501	Chlorociboria aeruginosa	OSC 100056	TypeSpecies	Chlorociboriaceae	Chlorociboriaceae	
AY755355	Chlorociboria halonata	PDD 71610		Chlorociboriaceae	Chlorociboriaceae	
AY755352	Chlorociboria poutoensis	ICMP 15618		Chlorociboriaceae	Chlorociboriaceae	
D1600	Chlorociboria sp.	ICMP 15637		Chlorociboriaceae	Chlorociboriaceae	GenBank MH921848
AY755361	'Chlorociboria' sp.	PDD 70096				
MG553993	Chlorosplenium chlora	BHIF736a	TypeSpecies	Helotiales incertae sedis	Mollisiaceae	
KF429256	Chlorovibrissea albofusca	ICMP 19443		Vibrisseaceae	Helotiales helotioid clade	
KF924737	Chlorovibrissea bicolor	ICMP 19895	Type Species	Vibrisseaceae	Helotiales helotioid clade	
KF429258	Chlorovibrissea chilensis	PDD 99891		Vibrisseaceae	Helotiales helotioid clade	
KF429261	Chlorovibrissea phialophora	PDD 83226		Vibrisseaceae	Helotiales helotioid clade	
PRJNA289037	Ciborinia camelliae	ICMP 19812		Sclerotiniaceae	Sclerotiniaceae	ITS extracted from NCBI genome
Z73768	Ciborinia whetzelii	1927.H (Oslo)	TypeSpecies	Sclerotiniaceae	Sclerotiniaceae	
JN033429	Cistella albidolutea	KUS F52678		Hyaloscyphaceae	Stamnaria lineage/Han Clade 9	
KC834043	Cladochasiella divergens	CCM F-13489	exTypeSpecimen	Ascomycota incertae sedis (2)	Discinella-Pezoloma lineage	
EF029222	Clathrosphaerina zalewskii	ICMP 15322	TypeSpecies	Hyaloscyphaceae (2)	Arachnopezizaceae	
EF029202	Clathrosporium intricatum	ICMP 14914	TypeSpecies	Helotiales incertae sedis (2)	Gelatinodiscaceae	
C_atro_Hosoya	Claussenomyces atrovirens	TNS-F-18149		Tympanidaceae	Leotiales incertae sedis	Genbank LC425048

Genbank accession or code	Species	Voucher	Type status of specimen (3)	Family (1, 2)	Accepted family	Notes
KM677201	'Claussenomyces' sp.	ICMP 21969		Tympanidaceae	Leotiales incertae sedis	
KC492060	Cleistothelebolus nipigonensis	Doveri	Type Species	Thelebolaceae	Thelebolaceae	
MH578517	Coccomyces crystalligenus	PDD 111539		Rhytismataceae	Rhytismataceae	
DQ491499	Coccomyces dentatus	OSC 100021		Rhytismataceae	Rhytismataceae	
R999	Coccomyces globosus	ICMP 17374		Rhytismataceae	Rhytismataceae	GenBank MH921849
KJ606678	Coccomyces lauraceus	ICMP 17399		Rhytismataceae	Rhytismataceae	
MH578530	Coccomyces phyllocladi	PDD 112232		Rhytismataceae	Rhytismataceae	
R970	Coccomyces radiatus	ICMP 17372		Rhytismataceae	Rhytismataceae	GenBank MH921850
MG386025	Cochlearomyces eucalypti	CBS 142622	exTypeSpecimen	Cochlearomycetaceae	Cochlearomycetaceae	
NR_154804	Coleophoma camelliae	CBS 101376		Leotiomycetes incertae sedis	Dermateaceae	
NR_154805	Coleophoma ericicola	CBS 301.72		Leotiomycetes incertae sedis	Dermateaceae	
KU728491	Coleophoma paracylindrospora	CBS 109074		Leotiomycetes incertae sedis	Dermateaceae	
NR_154807	Coleophoma parafusiformis	CBS 132692		Leotiomycetes incertae sedis	Dermateaceae	
NR_111760	Coleophoma proteae	CBS 132532		Leotiomycetes incertae sedis	Dermateaceae	
NR_154838	Coleophoma xanthosiae	CPC 29214		Leotiomycetes incertae sedis	Dermateaceae	
NR_111443	Collembolispora barbata	CBS 115944	exTypeSpecimen	Helotiales incertae sedis (2)	Ploettnerulaceae	
GQ154575	'Collophorina' paarla	CBS 120878			Leotiales incertae sedis	
GQ154547	Collophorina rubra	CBS 120873	exTypeSpecimen	Tympanidaceae	Tympanidaceae	
AJ293880	Colpoma quercinum	strain C3	Type Species	Rhytismataceae	Rhytismataceae	
KJ755499	Connersia rilstonii	CBS 537.74	exTypeSpecimen	Pseudeurotiaceae	Helotiaceae	
Z81428	Coprotinia minutula	Holst Jensen 1916.P	Type Species	Sclerotiniaceae	Sclerotiniaceae	ITS tree Lachnaceae but on long branch, ITS1 only and not great quality; also on long branch in Hoslt Jensen phylogeny
KT225524	Crinula caliciiformis	AFTOL-ID 272	TypeSpecies	Tympanidaceae	Leotiomycetes incertae sedis	
KT462570	Cristulariella depraedans	KUS F26976	Type Species	Sclerotiniaceae	Sclerotiniaceae	
604a	Crocicreas gramineum	YSU-F-03527	TypeSpecies	incertae sedis	Helotiales - Han Clade 4	GenBank MH733945
MH578514	'Crocicreas' multicuspidatum	PDD 112218				
LT158442	Crumenulopsis sororia	TU 104504		Cenangiaceae	Cenangiaceae	
MH578491	Cryptohymenium pycnidiophorum	PDD 111534	TypeSpecies	Helotiales incertae sedis	Helotiales sclerotinioid clade	
KR859232	Cryptosporiopsis nigra	CBS 268.39	TypeSpecies	Dermateaceae	Dermateaceae	
EU784189	Cudonia circinans	K(M)102575	Type Species	Cudoniaceae	Cudoniaceae	
DQ202512	Cudoniella acicularis	CBS 100273		Helotiaceae	Helotiaceae	
DQ491502	Cudoniella clavus	AFTOL-ID 166		Helotiaceae	Helotiaceae	
DQ202513	Cudoniella indica	CBS 430.94		Helotiaceae	Helotiaceae	
DQ202505	Cudoniella indica	SS 708		Helotiaceae	Helotiaceae	
KM503089	Curviclavula anemophila	CBS 138123	exTypeSpecimen	Helotiales incertae sedis (2)	Pezizellaceae	
AJ430397	Cyathicula coronata	ARON3093.H	TypeSpecies	Helotiaceae	Helotiaceae	
NR_153910	Cyclaneusma minus	CBS 496.73	exTypeSpecimen	Marthamycetaceae	Marthamycetaceae	
EU107253	Cyttaria darwinii	isolate 14 (Farlow)	TypeSpecies	Cyttariaceae	Cyttariaceae	
U92302	Darkera parca	Gernandt	exTypeSpecimen	Phacidiaceae	Phacidiaceae	
AB481239	Dasyscyphella longistipitata	FC-2013		Lachnaceae	Lachnaceae	
NR_154008	Davidhawksworthia ilicicola	CBS 734.94	exTypeSpecimen	Dermateaceae	Dermateaceae	
JQ688406	Deltopyxis triangulispora	Baral HB9625b	exTypeSpecimen	Leotiomycetes incertae sedis	Leotiomycetes incertae sedis	
HF677177	Dematioscypha delicatum	FMR 11585		cf Hyaloscyphaceae	Helotiales - Han Clade 7	
JN033438	Dematioscypha dematiicola	TNS-F-17834	type species	cf Hyaloscyphaceae	Helotiales - Han Clade 7	
PDD102323	'Dermatea' fumosa	PDD 102323				GenBank MH921851
AF141164	Dermea acerina	CBS 131.68		Dermateaceae	Dermateaceae	
AF141159	Dermea cerasi	CBS 136.46	TypeSpecies	Dermateaceae	Dermateaceae	
KF727411	Dicephalospora chrysotricha	ICMP 19952		Helotiaceae	Helotiaceae	
10106	Dicephalospora rufocornea	HMAS 275559		Helotiaceae		GenBank KU668565
AB926055	Dicephalospora rufocornea	TNS-F-40024		Helotiaceae	Helotiaceae	
D2146	'Dicephalospora rufo-cornea'	ICMP 22840				GenBank MH921852; ex Phormium ex New Zealand
DQ202518	Dimorphospora foliicola	CBS 221.59	exTypeSpecimen	Helotiaceae (2)	Gelatinodiscaceae	
KY672995	Diplocarpon mali	NL1		Drepanopezizaceae	Drepanopezizaceae	NCBI genome also available for this specimen; as Marsonnina coronariae
DortE4	Diplocarpon rosae	DortE4	TypeSpecies	Drepanopezizaceae	Drepanopezizaceae	ITS extracted from NCBI genome
KP052773	Diplocarpon rosae	DR19	TypeSpecies	Drepanopezizaceae	Drepanopezizaceae	
KJ559532	Diplolaeviopsis cf ranula	Diederich 16989	Type Species	Cordieritidaceae	Cordieriticaeae	
KC412001	Discinella boudieri	Baral HB4326	TypeSpecies	Discinella-Pezoloma lineage	Discinella-Pezoloma lineage	

Genbank accession or code	Species	Voucher	Type status of specimen (3)	Family (1, 2)	Accepted family	Notes
KY462820	Duebenia compta	G.M. 2015-06-01-1		Calloriaceae	Stamnaria lineage/Han Clade 9	
AB516665	Dumontinia tuberosa	MAFF 241373	TypeSpecies	Sclerotiniaceae	Sclerotiniaceae	
KY462811	Durella connivens	GM-2015-05-16		Strossmayeria lineage	Strossmayeria lineage	
KY462812	'Durella' macrospora	Baral HB9974				
MF161317	'Durella' melanochlora	BHI-F652a				
NR_126148	Eleutheromyces subulatus	CBS 113.86	exTypeSpecimen	Helicogoniaceae	Helicogoniaceae	epitype (MBT178280)
LT158475	Elliottinia kerneri	TU 104529	Type Species	Sclerotiniaceae	Sclerotiniaceae	
AF203469	Elytroderma deformans	CBS 181.68	Type Species	Rhytismataceae	Rhytismataceae	
KL111	Encoelia fimbriata	TAAM 165728	TypeSpecies	Cordieriticaeae	Cordieriticaeae	GenBank MH752063
LT158416	Encoelia furfuracea	TAAM 165633		Cenangiaceae	Cordieriticaeae	
LT158438	Encoelia glauca	Baral HB9232		Encoeliaceae	Cordieriticaeae	
LT158462	'Encoelia' pruinosa	NY02533482				KL344
KF727424	Endoscypha perforans	PDD 102231	Type Species	Hyaloscyphaceae	Helotiaceae	
KY661614	Epicladonia sandstedei	RP106	Type Species	Ascomycota incertae sedis (2)	Phacidiaceae	
KX501126	Eriopezia caesia	SBRH 843	Type Species	Arachnopezizaceae	Arachnopezizaceae	
KP794929	Erysiphe lespedezae	CFSZ 95119		Erysiphaceae	Erysiphaceae	
KY660860	Erysiphe necator	4_74		Erysiphaceae	Erysiphaceae	
U92304	Fabrella tsugae	Gernandt	TypeSpecies	Cenangiaceae	Cenangiaceae	
CCM_F00681	Filosporella annelidica	CCM F-00681		Ascomycota incertae sedis (2)	Helotiales incertae sedis	GenBank MK226458
KC834047	Filosporella fistucella	CCM F-13091		Ascomycota incertae sedis (2)	Helotiales incertae sedis	
KC834050	Flagellospora curvula	CCM F-18699	TypeSpecies	Nectriaceae (2)	Leotiales incertae sedis	
KP234354	Fontanospora eccentrica	CCM F-11513	TypeSpecies	Helotiales incertae sedis (2)	Discinella-Pezoloma lineage	
KF730813	Fontanospora eccentrica	UMB 882.11	Type Species	Helotiales incertae sedis (2)	Discinella-Pezoloma lineage	
DQ195779	Fulvoflamma eucalypti	CPC 11243	exTypeSpecimen	Rhytismatales incertae sedis (2)	Phacidiaceae	
KY853444	Fuscosclera lignicola	FMR11236	exTypeSpecimen	Dermateaceae (2)	Mollisiaceae	
PRJNA407395	Gamarada debralockiae	T6G9	TypeSpecies		Helotiales - Han Clade 4	ITS extracted from NCBI genome
G_ast_Hosoya	Gelatinipulvinella astraeicola	TNS-F-17846	TypeSpecies	Helicogoniaceae	Helicogoniaceae	Genbank LC425040
EU652349	Gelatinodiscus flavidus	OSC 65796	Type Species	Gelatinodiscaceae	Gelatinodiscaceae	
JX219380	Gelatinomyces siamensis	KKUK2	exTypeSpecimen	Tympanidaceae	Leotiales incertae sedis	synonym of Myriodiscus (Baral 2015)
G_fung_Hosoya	Gelatinopsis fungicola	TNS-F-44003		Helicogoniaceae	Helicogoniaceae	Genbank LC425051
KJ559542	Geltingia associata	TU 45655	Type Species	Helicogoniaceae	Helicogoniaceae	
KX501127	Gemmina gemmarum	SBRH 862	Type Species	Pezizellaceae	Pezizellaceae	
NR_111872	Geomyces auratus	CBS 108.14	exTypeSpecimen	Pseudeurotiaceae	Pseudeurotiaceae	
NR_137138	Glarea lozoyensis	ATCC 20868	exTypeSpecimen	Helotiaceae	Helotiaceae	JGI genome also available for this specimen
DQ235697	Gloeotinia temulenta	GS01	TypeSpecies	Helotiaceae	Helotiales incertae sedis	
KL150	Godronia fuliginosa	Baral HB6640		Godroniaceae	Godroniaceae	GenBank MH752064
AB077679	Golovinomyces ambrosiae	MUMH731		Erysiphaceae	Erysiphaceae	
AB769417	Golovinomyces asterum var. solidaginis	MUMH941		Erysiphaceae	Erysiphaceae	
AB077685	Golovinomyces macrocarpus	MUMH864		Erysiphaceae	Erysiphaceae	
KC834058	Gorgomyces hungaricus	CCM F-12696	exTypeSpecimen	Ascomycota incertae sedis (2)	Leotiales incertae sedis	
JQ256423	Graddonia coracina	ILLS 60491	TypeSpecies	Helotiaceae	Helotiaceae	
KC352953	Gremmeniella abietina	DAOM 170387	Type Species	Godroniaceae	Godroniaceae	
Greab	Gremmeniella abietina	DAOM 170408	TypeSpecies	Godroniaceae	Godroniaceae	GenBank KC352960
KX358851	Grovesiella abieticola	RFap2016WA 2	Type Species	Tympanidaceae	Godroniaceae	
KC460209	Grovesinia moricola	KUS F26901		Sclerotiniaceae	Sclerotiniaceae	
Z81433	Grovesinia pyramidalis	LMK38	TypeSpecies	Sclerotiniaceae	Sclerotiniaceae	
NR_111199	Gymnostellatospora canadensis	UAMH 8899		Myxotrichaceae	Pseudeurotiaceae	
NR_111200	Gymnostellatospora frigida	UAMH 9304		Myxotrichaceae	Pseudeurotiaceae	
KC834061	Gyoerffyella rotula	CCM F-400	TypeSpecies	Discinella-Pezoloma lineage	Discinella-Pezoloma lineage	type status, Marvanova, TBMS 65: 555-565, 1975
AF169303	Halenospora varia	ATCC 28878	TypeSpecies	Leotiaceae (2)	Helotiaceae	as Zalerion varium
JN033455	Hamatocanthoscypha laricionis	TNS-F-24336	type species	Pezizellaceae	Pezizellaceae	
D2116	Hamatocanthoscypha sp.	ICMP 22841		Pezizellaceae	Pezizellaceae	GenBank MH921853
AB329720	Haradamyces foliicola	MAFF 411026	exTypeSpecimen	Sclerotiniaceae	Sclerotiniaceae	
KR078433	Helicocentralis hyalina	SS04733.01	exTypeSpecimen	Leotiomycetes incertae sedis (2)	Helotiales hyaloscyphoid clade	
EF029238	'Helicodendron' luteoalbum	ICMP 15709				
KR078436	Helicodendron paradoxum	CBS 300.50	Type Species	Helotiales incertae sedis (2)	Gelatinodiscaceae	
EF029223	Helicodendron paradoxum	ICMP 15323	TypeSpecies	Helotiales incertae sedis (2)	Gelatinodiscaceae	

Genbank accession or code	Species	Voucher	Type status of specimen (3)	Family (1, 2)	Accepted family	Notes
EF029195	'Helicodendron' tubulosum	ICMP 14615				
EF029197	'Helicodendron' websteri	ICMP 14766				
EF029196	'Helicodendron' westerdijkae	ICMP 14616				
MH682236	Helotiaceae sp	ICMP 22837				
EU440381	Helotiales ex Damnamenia	PDD 93520				
KT876979	Helotiales sp.	Baral HB9934a				
D2515	Helotiales sp.	PDD 112247				GenBank MH921854; tiny blue green on Dracophyllum leaf
MH578568	'Helotium' elaeocarpi	ICMP 22808				
KY462819	'Helotium' uvidulum	G.M. 2015-06-06-1				
KY462821	Heterosphaeria patella	G.M. 201408041	TypeSpecies	Heterosphaeriaceae	Heterosphaeriaceae	
AY789297	Heyderia abietis	HMAS 71954	Type Species	Cenangiaceae	Cenangiaceae	
AY789290	Heyderia abietis	OSC 60392	Type Species	Cenangiaceae	Cenangiaceae	
LT158426	Heyderia abietis	TAAM 165961	TypeSpecies	Cenangiaceae	Cenangiaceae	
LT158430	Heyderia pusilla	TU 104257		Cenangiaceae	Cenangiaceae	
Au09_2	Hispidula sp.	ICMP 22842		Hyaloscyphaceae	Helotiaceae	GenBank MH921855; on Dicksonia
Au09_41	Hispidula sp.	ICMP 22843		Hyaloscyphaceae	Helotiaceae	GenBank MH921856; on Nothofagus
MH578493	Hispidula tokerau	ICMP 22800		incertae sedis	Helotiaceae	
H_muc_Hosoya	Holwaya mucida	TNS-F-29145	TypeSpecies	Tympanidaceae	Leotiomycetes incertae sedis	GenBank LC425042
TU112863	Holwaya mucida	TU 112863	TypeSpecies	Tympanidaceae	Leotiomycetes incertae sedis	GenBank MH752062
EU040232	Hyalodendriella betulae	CBS 261.82	exTypeSpecimen	Helotiales incertae sedis (2)	Pezizellaceae	
JN033416	Hyalopeziza leuconica	KUS F52474		Hyaloscyphaceae	Hyaloscyphaceae	
JN033381	Hyalopeziza nectrioides	CBS 597.77		Hyaloscyphaceae	Hyaloscyphaceae	
JN033439	Hyalopeziza sp	TNS-F-17879		Hyaloscyphaceae	Hyaloscyphaceae	
JN033442	Hyalopeziza sp	TNS-F-17975		Hyaloscyphaceae	Hyaloscyphaceae	
JN033449	Hyalopeziza sp	TNS-F-18048		Hyaloscyphaceae	Hyaloscyphaceae	
JN033437	Hyaloscypha albohyalina var albohyalina	TNS-F-11213		Hyaloscyphaceae	Hyaloscyphaceae	
JN033456	Hyaloscypha albohyalina var monodictys	TNS-F-5013		Hyaloscyphaceae	Hyaloscyphaceae	
JN033426	Hyaloscypha albohyalina var spiralis	KUS F52652		Hyaloscyphaceae	Hyaloscyphaceae	
JN033394	Hyaloscypha aureliella	KUS F52070		Hyaloscyphaceae	Hyaloscyphaceae	
EU940230	Hyaloscypha fuckelii	TUR 172135		Hyaloscyphaceae	Hyaloscyphaceae	
JN943614	Hyaloscypha hepaticicola	M339		Hyaloscyphaceae	Hyaloscyphaceae	
JN033451	Hyaloscypha leuconica var. bulbopilosa	TNS-F-18073		Hyaloscyphaceae	Hyaloscyphaceae	
KY769526	Hyaloscypha minuta	G.M. 20150406.2	TypeSpecies	Hyaloscyphaceae	Hyaloscyphaceae	
TTT2178	Hyaloscypha sp.	ICMP 22844		Hyaloscyphaceae	Hyaloscyphaceae	GenBank MH921857
JN033378	Hyaloscypha vitreola	CBS 127.91		Hyaloscyphaceae	Hyaloscyphaceae	
FJ477059	Hyaloscypha vitreola	Huhtinen M220	TypeSpecies	Hyaloscyphaceae	Hyaloscyphaceae	
KC834062	Hydrocina chaetocladia	CCM F-10890	exTypeSpecimen	Discinella-Pezoloma lineage	Helotiales incertae sedis	
NR_154907	'Hymenoscyphus' aurantiacus	HMAS 264143			Helotiaceae	
AB926065	Hymenoscyphus caudatus	TNS-F-40056		Helotiaceae	Helotiaceae	
CBS133217	Hymenoscyphus fraxineus	CBS 133217		Helotiaceae	Helotiaceae	ITS extracted from NCBI genome
GU586933	Hymenoscyphus fructigenus	CBS 650.92	TypeSpecies	Helotiaceae	Helotiaceae	
AB926057	Hymenoscyphus fructigenus	TNS-F-44644	TypeSpecies	Helotiaceae	Helotiaceae	
NR_119669	Hymenoscyphus ginkgonis	KUS F51352		Helotiaceae	Helotiaceae	
NR_137108	'Hymenoscyphus' haasticus	ICMP 19598		Helotiaceae		
CBS122016	Hymenoscyphus infarciens	CBS 122016		Helotiaceae	Helotiaceae	ITS extracted from NCBI genome
NR_137110	'Hymenoscyphus' kiko	ICMP 19613		Helotiaceae		
AB926063	Hymenoscyphus menthae	TNS-F-40052		Helotiaceae	Helotiaceae	
D1039	'Hymenoscyphus' metrosideri	ICMP 22845				GenBank MH921858
NR_137109	'Hymenoscyphus' ohakune	ICMP 19601		Helotiaceae		
PRJNA297738	'Hymenoscyphus' repandus	CBS 341.76				ITS extracted from NCBI genome
CBS111550	Hymenoscyphus salicellus	CBS 111550		Helotiaceae	Helotiaceae	ITS extracted from NCBI genome
KC481695	Hymenoscyphus scutula	CBS 480.97		Helotiaceae	Helotiaceae	NCBI genome also available for this specimen
AB926114	Hymenoscyphus scutula	TNS-F-17507		Helotiaceae	Helotiaceae	
AB481291	'Hymenoscyphus' varicosporoides	FC-2038		Helotiaceae		
Hymvar1	'Hymenoscyphus' varicosporoides	Hymvar1		Helotiaceae		ITS extracted from JGI genome
KC164666	'Hymenoscyphus' waikaia	PDD 66379		Helotiaceae		
MH578484	Hymenotorrendiella dingleyae	ICMP 22793		Helotiaceae	Helotiaceae	NCBI genome also available for this specimen

Genbank accession or code	Species	Voucher	Type status of specimen (3)	Family (1, 2)	Accepted family	Notes
MH578483	Hymenotorrendiella eucalypti	ICMP 22792	TypeSpecies	Helotiaceae	Helotiaceae	
AB546944	Hyphodiscus hyaloscyphoides	TNS-F-13588		Hyaloscyphaceae	Helotiales - Han Clade 4	
MH682230	Hyphodiscus sp.	ICMP 22833		Hyaloscyphaceae	Helotiales - Han Clade 4	NCBI genome also available for this specimen
AB546953	Hyphodiscus theiodeus	TNS-F-32000	Type Species	Hyaloscyphaceae	Helotiales - Han Clade 4	
JN033448	Hyphopeziza pygmaea	TNS-F-17940		Hyaloscyphaceae	Helotiales - Han Clade 4	
MH682238	Hypoderma cordylines	ICMP 22834		Rhytismataceae	Rhytismataceae	
R898	Hypoderma liliense	ICMP 18323		Rhytismataceae	Rhytismataceae	GenBank MH921859
JF683419	Hypoderma rubi	ICMP 17339	TypeSpecies	Rhytismataceae	Rhytismataceae	
KT876985	Incrucipulum ciliare	Baral HB9922	TypeSpecies	Lachnaceae	Lachnaceae	
AB481261	Incrucipulum radiatum	FC-2283, TNS-F-16769		Lachnaceae	Lachnaceae	
KR859078	Infundichalara microchona	CBS 175.74	exTypeSpecimen	Helotiales incertae sedis (2)	Pezizellaceae	
KL154	Ionomidotis irregularis	TAAM 198520	TypeSpecies	Cordieriticaeae	Cordieriticaeae	GenBank MH752065
KL301	Ionomidotis olivascens	TNS-F-39269		Cordieriticaeae	Cordieriticaeae	GenBank MH752068
AY236423	Kohninia linnaeicola	ARON 3886	TypeSpecies	Sclerotiniaceae	Sclerotiniaceae	
MH578545	Lachnellula rhopalostyilidis	ICMP 22817		Lachnaceae	Lachnaceae	
AB481247	Lachnellula subtilissima	FC 2354		Lachnaceae	Lachnaceae	
NR_154125	Lachnopsis catarinensis	CPC 24723	exTypeSpecimen	Hyaloscyphaceae	Lachnaceae	
JN033395	Lachnum abnorme	KUS F52080		Lachnaceae	Lachnaceae	
D2142	Lachnum berggrenii	ICMP 22847		Lachnaceae	Lachnaceae	GenBank MH921860
D2179	Lachnum filiceum	ICMP 22848		Lachnaceae	Lachnaceae	GenBank MH921861
MH682233	Lachnum nothofagi	ICMP 22835		Lachnaceae	Lachnaceae	NCBI genome also available for this specimen
L_palm_Hosoya	Lachnum palmae	TNS-F-13500		Lachnaceae	Lachnaceae	GenBank LC425039
D832	Lachnum palmae	ICMP 22849		Lachnaceae	Lachnaceae	GenBank MH921862
D2018asc1	Lachnum pteridicola	ICMP 22850		Lachnaceae	Lachnaceae	GenBank MH921863
D1189	Lachnum pteridophyllum	ICMP 22851		Lachnaceae	Lachnaceae	GenBank MH921864
MH578538	Lachnum sp.	ICMP 22822		Lachnaceae	Lachnaceae	ex Dacrydium cupressinum
DQ491485	Lachnum virgineum	AFTOL-ID 49		Lachnaceae	Lachnaceae	
AB705235	Lachnum virgineum	FC 2137		Lachnaceae	Lachnaceae	
MH578513	'Lachnum' willisii	PDD 112217				
KT185666	Laetinaevia carneoflavida	G.M. 2014-07-25		Calloriaceae	Stamnaria lineage/Han Clade 9	
L_brun_Hosoya	'Lambertella' brunneola	TNS-F-44244		Rutstroemiaceae	Rutstroemiaceae	GenBank LC425046
AB926069	Lambertella cornimaris	TNS-F-40083	TypeSpecies	Rutstroemiaceae	Rutstroemiaceae	
D1109	Lambertella corni-maris	ICMP 22852	TypeSpecies	Rutstroemiaceae	Rutstroemiaceae	GenBank MH921865
AB926081	Lambertella pyrolae	TNS-F-40132		Rutstroemiaceae	Rutstroemiaceae	
KF545329	Lambertella subrenispora	CBS 811.85		Rutstroemiaceae	Rutstroemiaceae	
AY755334	Lanzia allantospora	ICMP 22836		Rutstroemiaceae	Rutstroemiaceae	
MH137197	Lanzia griseliniae	ICMP 20108		Rutstroemiaceae	Rutstroemiaceae	
MH578497	'Lanzia' ovispora	ICMP 22804				
KX228287	Lareunionomyces syzygii	CPC 26531	exTypeSpecimen	Neolauriomycetaceae (2)	Helotiales pezizelloid clade	
AB481284	Lasiobelonium lonicerae	FC 2270		Lachnaceae	Lachnaceae	
NR_137151	Lauriomyces bellulus	CBS 517.93		Lauriomycetaceae	Lauriomycetaceae	
NR_155326	Lauriomyces cylindricus	BCC 18576		Lauriomycetaceae	Lauriomycetaceae	
NR_155327	Lauriomyces ellipticus	BCC 4007		Lauriomycetaceae	Lauriomycetaceae	
KX649976	'Lauriomyces' sakaeratensis	SFC 01642				
CCM_F21799	Lemonniera aquatica	CCM F-21799	TypeSpecies	incertae sedis	Discinella-Pezoloma lineage	GenBank MK226460
NR_121306	Leohumicola verrucosa	CBS 115880	exTypeSpecimen	Leotiomycetes incertae sedis (2)	Stamnaria lineage/Han Clade 9	
EU784369	Leotia lubrica	K(M)67114	Type Species	Leotiaceae	Leotiaceae	
DQ491484	Leotia lubrica	OSC 100001	TypeSpecies	Leotiaceae	Leotiaceae	
AY129285	Leptodontidium elatius	CBS 329.53	exTypeSpecimen	Helotiales incertae sedis (2)	Helotiales - Han Clade 4	
AY354250	Leptodontidium elatius	olrim127 1		Helotiales incertae sedis (2)	Helotiales - Han Clade 4	
DQ069032	'Leptodontidium' orchidicola	aurim605				
MG597459	Leptodontidium sp.	isolate 0242		Helotiales incertae sedis (2)	Helotiales - Han Clade 4	
KY031672	'Leptodontidium' sp.	MR80				
KF049206	Leuconeurospora pulcherrima	AFTOL-ID 1397	exTypeSpecimen	Pseudeurotiaceae	Pseudeurotiaceae	
JX270336	Leuconeurospora sp	01NH01		Pseudeurotiaceae	Pseudeurotiaceae	
R870	Lophodermium agathidis	ICMP 17348		Rhytismataceae	Rhytismataceae	GenBank MH921866
KF727414	Lophodermium conigenum	PDD 103381		Rhytismataceae	Rhytismataceae	

Genbank accession or code	Species	Voucher	Type status of specimen (3)	Family (1, 2)	Accepted family	Notes
EF191235	Lophodermium eucalypti	ICMP 16796		Rhytismataceae	Rhytismataceae	
MH578524	Lophodermium hauturuanum	PDD 112227		Rhytismataceae	Rhytismataceae	
MH578540	Lophodermium rectangulare	PDD 112235		Rhytismataceae	Rhytismataceae	
R927	Lophodermium tindalii	ICMP 17357		Rhytismataceae	Rhytismataceae	GenBank MH921867
NR_138379	Loramyces macrosporus	CBS 235.53		Loramycetaceae	Mollisiaceae	JGI genome also available for this specimen
KP984784	Macroskyttea parmotrematis	UGDA	exTypeSpecimen	Helotiales incertae sedis (2)	Cordieritidaceae	
MH037296	Malbranchea flavorosea	ATCC 34529		Myxotrichaceae	Myxotrichaceae	
Marbr1	Marssonina brunnea	Marbr1		Drepanopezizaceae	Drepanopezizaceae	ITS extracted from JGI genome
Au97_130	Marthamyces barbatus	ICMP 22853		Marthamycetaceae	Marthamycetaceae	GenBank MH921868
MH682228	Marthamyces dracophylli	ICMP 22838		Marthamycetaceae	Marthamycetaceae	
Au97_123	Marthamyces emarginatus	ICMP 22854	TypeSpecies	Marthamycetaceae	Marthamycetaceae	GenBank MH921869; ex Eucalyptus Australia; type substrate
Au96_140	Marthamyces gilvus	ICMP 22855		Marthamycetaceae	Marthamycetaceae	GenBank MH921870
MH682227	Marthamyces sp.	ICMP 17398		Marthamycetaceae	Marthamycetaceae	ex Metrosideros, New Zealand
NR_126150	Mastigosporium album	CBS 138013	exTypeSpecimen	Ascomycota incertae sedis (2)	Ploettnerulaceae	
GQ406809	Medeolaria farlowii	DHP 07-637	Type Species	Medeolariaceae	Medeolariaceae	
Melbi2	Meliniomyces bicolor	Melbi2		Hyaloscyphaceae	Hyaloscyphaceae	ITS extracted from JGI genome
Melva1	Meliniomyces variabilis	Melva1	TypeSpecies	Hyaloscyphaceae	Hyaloscyphaceae	ITS extracted from JGI genome
NR_121313	Meliniomyces variabilis	UAMH 8861	exTypeSpecimen	Hyaloscyphaceae	Hyaloscyphaceae	
KY485137	Meloderma desmazierii	DAOM 89807	Type Species	Rhytismataceae	Rhytismataceae	
R239	'Meloderma' dracophylli	ICMP 17343		Rhytismataceae	Rhytismataceae	GenBank MH921871
KT225534	Meria laricis	CBS 298.52	TypeSpecies	Cenangiaceae	Cenangiaceae	
EU784373	Microglossum olivaceum	K(M)135599	TypeSpecies	Leotiaceae	Leotiaceae	
KL220	Microglossum olivaceum	TU 101778	TypeSpecies	Leotiaceae	Leotiaceae	GenBank MH752066
JN033418	Microscypha ellisii	KUS F52489		Pezizellaceae	Pezizellaceae	
KC834064	Miniancora allisoniensis	CCM F-30487	exTypeSpecimen	Ascomycota incertae sedis (2)	Leotiales incertae sedis	
AY789282	Mitrula borealis	DAOM 160731		Mitrulaceae	Mitrulaceae	
AY789434	Mitrula elegans	DMWV04-78		Mitrulaceae	Mitrulaceae	
AY789281	Mitrula lunulatospora	DAOM 160732		Mitrulaceae	Mitrulaceae	
AY789424	Mitrula paludosa	MBH 50636	TypeSpecies	Mitrulaceae	Mitrulaceae	
KX273438	Mitrulinia ushuaiae	PDD 105643	Type Species	Helotiales incertae sedis	Helotiaceae	
KX273437	Mitrulinia ushuaiae	PDD 107564	Type Species	Helotiales incertae sedis	Helotiaceae	
KU668566	Moellerodiscus lentus	HMAS 275557		Rutstroemiaceae	Rutstroemiaceae	
AB926078	Moellerodiscus pinicola	TNS-F-40115		Rutstroemiaceae	Rutstroemiaceae	
M_ame_Hosoya	'Mollisia' amenticola	TNS-F-30411		Mollisiaceae		GenBank LC425041
DQ491498	Mollisia cinerea	OSC 100029	TypeSpecies	Mollisiaceae	Mollisiaceae	
HM116746	'Mollisia' dextrinospora	ICMP 18083				
AY259138	Mollisia fusca	CBS 234.71		Mollisiaceae	Mollisiaceae	
AY259137	'Mollisia fusca'	CBS 486.48				
MG195562	'Mollisia' sp.	ICMP 21729				
JN033404	'Mollisina' uncinata	KUS F52307				
Z73778	Monilinia fructicola	LMK125	TypeSpecies	Sclerotiniaceae	Sclerotiniaceae	
AH009124	Mycoarthris corallinus	Landvik	exTypeSpecimen	Hyaloscyphaceae (2)	Stamnaria lineage/Han Clade 9	
AB469683	Mycochaetophora gentianae	MAFF 239231		Ploettnerulaceae	Ploettnerulaceae	
KC834065	Mycofalcella calcarata	CCM F-10289	exTypeSpecimen	incertae sedis	Helotiaceae	
AB254177	Mycopappus alni	CBS 893.85	TypeSpecies	Sclerotiniaceae	Sclerotiniaceae	
NR_137807	Mycosymbioces mycenophila	OSC 148294	exTypeSpecimen	Leotiomycetes incertae sedis (2)	Leotiales incertae sedis	
JX219379	Myriodiscus sparassoides	KKUK1	TypeSpecies	Tympanidaceae	Tympanidaceae	
LMK759	Myriosclerotinia curreyana	NCBI Genome		Sclerotiniaceae	Sclerotiniaceae	ITS extracted from NCBI genome
LMK746	Myriosclerotinia duriaeana	NCBI Genome		Sclerotiniaceae	Sclerotiniaceae	ITS extracted from NCBI genome
LMK735	Myriosclerotinia scirpicola	NCBI Genome	TypeSpecies	Sclerotiniaceae	Sclerotiniaceae	ITS extracted from NCBI genome
NR_119412	Myxocephala albida	CBS 962.87	exTypeSpecimen	Sordariomycetes (2)	Helotiales incertae sedis	
AF062813	Myxotrichum chartarum	UAMH 1997	Type Species	Myxotrichaceae	Myxotrichaceae	
Bills_F267_639	Naemacyclus fimbriatus	Gerald Bills F267-639	TypeSpecies	Marthamycetaceae	Marthamycetaceae	ITS available from ITS alignment, MWLR DataStore
AY853224	Naevala minutissima	CBS 115934	TypeSpecies	Calloriaceae	Discinella-Pezoloma lineage	
HM116779	Neobulgaria alba	ICMP 18395		Gelatinodiscaceae	Gelatinodiscaceae	MWLR genome also available for this specimen
DQ257366	Neobulgaria pura	CUP 063609	Type Species	Gelatinodiscaceae	Gelatinodiscaceae	
NR_156388	Neocrinula lambertiae	CBS 143423		Neocrinulaceae (2)	Neocrinulaceae	

Genbank accession or code	Species	Voucher	Type status of specimen (3)	Family (1, 2)	Accepted family	Notes
KY173412	Neocrinula xanthorrhoeae	CPC 29474	exTypeSpecimen	Neocrinulaceae (2)	Neocrinulaceae	
KY173414	Neocrinula xanthorrhoeae	CPC 29474	TypeSpecies	Neocrinulaceae (2)	Neocrinulaceae	
NR_121301	Neocudoniella radicella	UAMH 5794		Gelatinodiscaceae	Bryoglossum lineage	
MH578547	Neodasyscypha cerina	ICMP 22829		Lachnaceae	Lachnaceae	
U57812	Neodasyscypha cerina	JHH3916 NYS	Type Species	Lachnaceae	Lachnaceae	
NR_144926	Neofabraea malicorticis	CBS 102863	exTypeSpecimen	Dermateaceae	Dermateaceae	neotype
LC228670	Nervostroma depraedans	JCM 14252	exTypeSpecimen	Sclerotiniaceae	Sclerotiniaceae	
JF412009	Oculimacula yallundae	CBS 494.80	TypeSpecies	Ploettnerulaceae	Ploettnerulaceae	
NR_111035	Oidiodendron fuscum	UAMH 8511	exTypeSpecimen	Myxotrichaceae	Myxotricaceae	
Oidma1	Oidiodendron maius	JGIGenome		Myxotrichaceae	Myxotricaceae	ITS extracted from JGI genome
JN033380	Olla millepunctata	CBS 100302		Hyaloscyphaceae	Hyaloscyphaceae	
HQ533049	Orbiliopsis callistea	PDD 97932		Helotiales incertae sedis	Helotiales incertae sedis	
Z73797	Ovulinia azaleae	ATCC 1189	TypeSpecies	Sclerotiniaceae	Sclerotiniaceae	
KC833168	Pachycudonia constrictospora	C307	TypeSpecies	Cudoniaceae	Cudoniaceae	
NR_132835	Parafabraea eucalypti	CBS 124810	exTypeSpecimen	Dermateaceae	Dermateaceae	
KT876978	Patinella hyalophaea	Baral HB9739	TypeSpecies	Helotiales incertae sedis	Leotiomycetes incertae sedis	
U57988	Perrotia flammea	JHH4497 NYS	Type Species	Lachnaceae	Lachnaceae	
NR_144927	Pezicula carpinea	CBS 923.96	exTypeSpecimen	Dermateaceae	Dermateaceae	epitype
D1095	Pezicula sp.	ICMP 22856		Dermateaceae	Dermateaceae	GenBank MH921872; on Nothofagus
KJ559546	'Pezizella' epithallina	TU 39378				assumed to be misnamed, in Han Clade 9
MH578481	Pezizellaceae sp Phormium	PDD 111530				
MH578459	Pezizellaceae sp.	ICMP 22770				
MH682232	Pezizellaceae sp.	ICMP 22832				
MH578465	Pezizellaceae sp. Collospermum	ICMP 22776				
MH578469	Pezizellaceae sp. Knightia	ICMP 22780				
MH578474	Pezizellaceae sp. Nothofagus leaf	ICMP 22785				
MH578482	Pezizellaceae sp. on Cortaderia	PDD 111531				
KC412000	Pezoloma ciliifera	F265954		Discinella-Pezoloma lineage	Discinella-Pezoloma lineage	
KJ663841	Phacidium lacerum	AFTOL-ID 1253		Phacidiaceae	Phacidiaceae	
NR_145381	Phacidium lauri	CBS 308.68	exTypeSpecimen	Phacidiaceae	Phacidiaceae	epitype
D2457	Phaeohelotium confusum	PDD 112244		Helotiaceae	Helotiaceae	GenBank MH921873
KT876976	Phaeohelotium epiphyllum	Baral HB9911		Helotiaceae	Helotiaceae	
AB926061	Phaeohelotium epiphyllum	TNS-F-40042		Helotiaceae	Helotiaceae	
KC411992	Phaeohelotium geogenum	Baral HB7222A		Helotiaceae	Helotiaceae	
KC411991	Phaeohelotium monticola	Baral HB8612	TypeSpecies	Helotiaceae	Helotiaceae	
KC411988	Phaeohelotium undulatum	AH7337		Helotiaceae	Helotiaceae	
JN033424	Phialina lachnobrachyoides	KUS F52576		Pezizellaceae	Pezizellaceae	
MG195534	Phialocephala bamuru	PDD 56863		Mollisiaceae	Mollisiaceae	
NR_135931	Phialocephala dimorphospora	CBS 300.62	exTypeSpecimen	Mollisiaceae	Mollisiaceae	
MG195479	Phialocephala oblonga	PDD 58596		Mollisiaceae	Mollisiaceae	
NR_111319	Phialocephala piceae	UAMH 10851	exTypeSpecimen	Mollisiaceae	Mollisiaceae	
Phisc1	Phialocephala scopiformis	JGIGenome		Mollisiaceae	Mollisiaceae	ITS exctracted from JGI genome
MG195462	Phialocephala sp.	ICMP 21725		Mollisiaceae	Mollisiaceae	
PRJEB12348	Phialocephala subalpina	UAMH11012		Mollisiaceae	Mollisiaceae	ITS exctracted from NCBI genome
KR859275	Phlyctema vagabunda	CBS 109875	TypeSpecies	Dermateaceae	Dermateaceae	
LT158483	Piceomphale bulgarioides	TAAM 165289	TypeSpecies	Sclerotiniaceae	Sclerotiniaceae	
NR_119500	Pilidium acerinum	BPI 843555	exTypeSpecimen	Chaetomellaceae	Chaetomellaceae	epitype
KM677205	'Pirottaea' palmicola	ICMP 13383				MWLR genome also available for this isolate
KJ755519	Pleuroascus nicholsonii	CBS 345.73	TypeSpecies	Pseudeurotiaceae	Helotiaceae	
AB904505	Poculum pseudosydowianum	TNS-F-40071		Rutstroemiaceae	Rutstroemiaceae	
AY775056	Polydesmia pruinosa	CBS 160.92	TypeSpecies	Helotiales incertae sedis	Helotiales sclerotinioid clade	as Brefeldochium pruinosum (anamorph of P. pruinosa)
JN033453	Polydesmia pruinosa	TNS-F-12764	TypeSpecies	Helotiales incertae sedis	Helotiales sclerotinioid clade	
MG719690	Polyphilus sieberi	TT3B	TypeSpecies	Hyaloscyphaceae	Stamnaria lineage/Han Clade 9	
MH327809	Porodiplodia livistonae	CBS 144428	exTypeSpecimen	Porodiplodiaceae (2)	Pezizellaceae	
DQ491510	Potebniamyces pyri	AFTOL-ID 744	TypeSpecies	Phacidiaceae	Phacidiaceae	
U57990	Proliferodiscus alboviridis	Cantrell GA34	TypeSpecies	Lachnaceae	Lachnaceae	
MH682231	Proliferodiscus dingleyae	ICMP 21730		Lachnaceae	Lachnaceae	

Genbank accession or code	Species	Voucher	Type status of specimen (3)	Family (1, 2)	Accepted family	Notes
MH682229	Propolis farinosus	ICMP 17354		Marthamycetaceae	Marthamycetaceae	
JQ256425	Propolis versicolor	ILLS 60497		Marthamycetaceae	Marthamycetaceae	
EF029224	Pseudaegerita corticalis	ICMP 15324	TypeSpecies	Hyaloscyphaceae	Hyaloscyphaceae	
EF029235	'Pseudaegerita' viridis	ICMP 15542				
NR_111127	Pseudeurotium zonatum	CBS 329.36	exTypeSpecimen	Pseudeurotiaceae	Pseudeurotiaceae	AFTOL-ID 1912
KR859280	Pseudofabraea citricarpa	CBS 130532	exTypeSpecimen	Dermateaceae	Dermateaceae	
Pseel	Pseudographis elatina	CBS 651.97	TypeSpecies	Triblidiaceae	Rhytismataceae	ITS exctracted from JGI genome
Pseudest	Pseudogymnoascus destructans	JGIGenome		Pseudeurotiaceae	Pseudeurotiaceae	ITS exctracted from JGI genome
KJ755524	Pseudogymnoascus roseus	UAMH 1990	TypeSpecies	Pseudeurotiaceae	Pseudeurotiaceae	
MG385670	Pseudopezicula tetraspora	ATCC 6229	TypeSpecies	cf Drepanopezizaceae	Discinella-Pezoloma lineage	
D2527	'Pseudopeziza' colensoi	PDD 112240				GenBank MH921874
EU729125	Pseudopeziza medicaginis	Ap1		Drepanopezizaceae	Drepanopezizaceae	
KJ663860	Pseudophacidium ledi	CBS 377.59	TypeSpecies	Phacidiaceae	Phacidiaceae	
JN033396	Psilachnum staphyleae	KUS F52105		Pezizellaceae	Stamnaria lineage/Han Clade 9	
JX001640	Psychrophila antarctica	MMW-2013a isolate 92	exTypeSpecimen	Helotiaceae (2)	Helotiales incertae sedis	sister to Arachnopezizaceae in ITS tree, but support needs confirming with additional genes
KF859927	Pycnopeziza sympodialis	CBS 332.39	TypeSpecies	Sclerotiniaceae	Sclerotiniaceae	
MF143627	Pyrenopeziza brassicae	Cyc029a		Ploettnerulaceae	Ploettnerulaceae	
MF187547	Pyrenopeziza brassicae	PC18		Ploettnerulaceae	Ploettnerulaceae	
P_pro_Hosoya	'Pyrenopeziza' protrusa	TNS-F-23154				GenBank LC426322
AJ430224	'Pyrenopeziza' revincta	ARON3150.P				
JN033413	'Pyrenopeziza' sp.	KUS F52417				
MF593628	'Pyrenopeziza' velebitica	CNF 2 10097				
AF462422	Rhabdocline pseudotsugae	RPPWA29	Type Species	Cenangiaceae	Cenangiaceae	
NR_111086	Rhexocercosporidium carotae	CBS 418.65	exTypeSpecimen	Ploettnerulaceae	Ploettnerulaceae	
Rhesp1	Rhexocercosporidium sp.	MPI-PUGE-AT-0058		Ploettnerulaceae	Ploettnerulaceae	ITS extracted from JGI genome
NR_145279	Rhizocladosporium argillaceum	CBS 241.67	exTypeSpecimen	Helotiales incertae sedis (2)	Helotiales incertae sedis	
NR_137757	Rhizodermea veluwensis	CBS 110605	exTypeSpecimen	Dermateaceae	Dermateaceae	
NR_111110	Rhizoscyphus ericae	UAMH 6735	exTypeSpecimen	Hyaloscyphaceae	Hyaloscyphaceae	
JN033414	'Rodwayella' citrinula	KUS F52443		Pezizellaceae		
KT876974	Rodwayella sessilis	Baral HB9913	TypeSpecies	Pezizellaceae	Pezizellaceae	
EF060298	Roesleria subterranea	CBS 407.51	TypeSpecies	Roesleriaceae	Helotiaceae	
R_sub_Hosoya	Roesleria subterranea	TNS-F-38701	TypeSpecies	Roesleriaceae	Helotiaceae	GenBank AB628057
KT958773	Rommelaarsia flavovirens	Baral HB9951b	exTypeSpecimen	Helotiales incertae sedis (2)	Stamnaria lineage/Han Clade 9	
KT972711	'Roseodiscus' formosus	FH (SBRH 686)				
DH257	Roseodiscus rhodoleucus	HB 8488a	TypeSpecies	incertae sedis	Stamnaria lineage/Han Clade 9	GenBank KT972704
AB926053	Rutstroemia bulgarioides	TNS-F-40005		Rutstroemiaceae	Rutstroemiaceae	
KF545321	Rutstroemia echinophila	CBS 111548		Rutstroemiaceae	Rutstroemiaceae	
KF545334	Rutstroemia firma	AFTOL-ID 923	TypeSpecies	Rutstroemiaceae	Rutstroemiaceae	
KF545331	Rutstroemia sydowiana	CBS 115975		Rutstroemiaceae	Rutstroemiaceae	
LT158423	Rutstroemia tiliacea	TAAM 132844		Rutstroemiaceae	Rutstroemiaceae	
NR_137991	Sabahriopsis eucalypti	CPC 24957	exTypeSpecimen	Helotiales incertae sedis (2)	Cordieritidaceae	
AY590786	Sarea difformis	olrim59	Type Species			
DQ195784	Satchmopsis brasiliensis	CBS 420.93	TypeSpecies	Pezizomycotina incertae sedis (2)	Leotiales incertae sedis	
DQ195786	Satchmopsis brasiliensis	CPC 11017	TypeSpecies	Pezizomycotina incertae sedis (2)	Leotiales incertae sedis	
LT158464	'Sclerencoelia' fascicularis	TU 104508				
AY789408	Scleromitrula shiraiana	Hirayama062001	TypeSpecies	Sclerotiniaceae	Rutstroemiaceae	
Z81448	Scleromitrula spiraeicola	strain 1336.1	Type Species	Sclerotiniaceae	Rutstroemiaceae	
AF141169	Scleropezicula alnicola	CBS 474.97	TypeSpecies	Pezizellaceae	Pezizellaceae	
Sclsc	Sclerotinia sclerotiorum	ATCC 18683	TypeSpecies	Sclerotiniaceae	Sclerotiniaceae	ITS extracted from JGI genome
KF859932	Sclerotinia sclerotiorum	DAOM 241671	Type Species	Sclerotiniaceae	Sclerotiniaceae	
IHIA52	Scytalidium lignicola	DSM 105466		Leotiomycetes incertae sedis (2)	Helotiales incertae sedis	ITS extracted from NCBI genome
NR_121314	Scytalidium lignicola	UAMH 1502	exTypeSpecimen	Leotiomycetes incertae sedis (2)	Helotiales incertae sedis	
U57991	Solenopezia solenia	JHH4169 NYS	Type Species	Lachnaceae	Lachnaceae	
JX124327	Soosiella minima	MH-2012 strain 1230	exTypeSpecimen	Helotiales incertae sedis (2)	Helotiales - Han Clade 4	
Spafl1	Spathularia flavida	Spafl1	TypeSpecies	Cudoniaceae	Cudoniaceae	ITS extracted from JGI genome
AF433155	Spathularia flavida	wz95	Type Species	Cudoniaceae	Cudoniaceae	

Genbank accession or code	Species	Voucher	Type status of specimen (3)	Family (1, 2)	Accepted family	Notes
NR_119916	Sphaerographium nyssicola	CBS 128284		Chaetomellaceae	Chaetomellaceae	
HQ696658	Spirosphaera floriformis	CBS 402.52	exTypeSpecimen	Helotiales incertae sedis (2)	Helotiaceae	
KT972707	Stamnaria americana	D. Haelew. 258a		Stamnaria lineage	Stamnaria lineage/Han Clade 9	
MG662189	Stamnaria americana	D. Haelew. 941d		Stamnaria lineage	Stamnaria lineage/Han Clade 9	
KT972708	Stamnaria austriaca	940a		Stamnaria lineage	Stamnaria lineage/Han Clade 9	
MG662201	Stamnaria persooni	D. Haelew. 671a		Stamnaria lineage	Stamnaria lineage/Han Clade 9	
MG662203	Stamnaria yugrana	D. Haelew. 603a		Stamnaria lineage	Stamnaria lineage/Han Clade 9	
AY818330	Streptobotrys streptothrix	DAOM 37579	Type Species	Sclerotiniaceae		as Streptobotrys streptothrix
Z73801	Stromatinia rapulum	strain 1243.1	Type Species	Sclerotiniaceae	Sclerotiniaceae	
S_bak_Hosoya	Strossmayeria bakeriana	TNS-F-11199		Strossmayeria lineage	Strossmayeria lineage	GenBank LC425043
KT876977	Strossmayeria basitricha	Baral HB9904b		Strossmayeria lineage	Strossmayeria lineage	
KY462386	Strossmayeria sp.	DHP-CH-108		Strossmayeria lineage	Strossmayeria lineage	
JX989830	Synchaetomella acerina	DAOM 242271		Chaetomellaceae	Chaetomellaceae	
T_fusc_Hosoya	Tapesia fusca	TNS-F-17463	TypeSpecies	Mollisiaceae	Mollisiaceae	GenBank LC425049
AY129291	Teberdinia hygrophila	CBS 102670	exTypeSpecimen	Pseudeurotiaceae	Pseudeurotiaceae	
AY100665	Terriera minus	ICMP 13974		Rhytismataceae	Rhytismataceae	
KC834066	Tetrachaetum elegans	CBM11	TypeSpecies	Ascomycota incertae sedis (2)	Discinella-Pezoloma lineage	
JX029109	Tetracladium globosum	isolate 215		Helotiales incertae sedis	Stamnaria lineage/Han Clade 9	
CCM_F26199	Tetracladium marchalianum	CCM F-26199	TypeSpecies	Helotiales incertae sedis	Stamnaria lineage/Han Clade 9	
EU883424	Tetracladium palmatum	CCM F-10001		Helotiales incertae sedis	Stamnaria lineage/Han Clade 9	
KJ559535	Thamnogalla crombiei	Diederich 17553	Type Species	Cordieritidaceae	Cordieritidaceae	
GU269839	Thedgonia ligustrina	CBS 132025	TypeSpecies	Drepanopezizaceae	Drepanopezizaceae	
FJ839630	Thedgonia ligustrina	CPC 14754	Type Species	Drepanopezizaceae	Drepanopezizaceae	
Thest1	Thelebolus stercoreus	Thest1	Type Species	Thelebolaceae	Thelebolaceae	ITS extracted from JGI genome
JQ256427	Thuemenidium atropurpureum	ILLS 61044		Leotiaceae	Leotiaceae	
NR_132907	Tiarosporella paludosa	CPC 22701	exTypeSpecimen	Phacidiaceae	Leotiomycetes incertae sedis	epitype
KC412008	Torrendiella ciliata	F132996	TypeSpecies	Rutstroemiaceae	Rutstroemiaceae	
JN033398	Trichopeziza sulphurea	KUS F52218	TypeSpecies	Lachnaceae	Lachnaceae	
U57813	Trichopezizella nidulus	JHH4485 NYS	Type Species	Lachnaceae	Lachnaceae	
T_ota_Hosoya	Trichopezizella otanii	FC-2156, TNS-F-16579		Lachnaceae	Lachnaceae	GenBank AB481287
JQ417289	'Tricladium' kelleri	CBS 130985				
KC834068	Tricladium obesum	CCM F-14598		Helotiaceae (2)	Helotiaceae	
AY204635	Tricladium splendens	CCM F-16599	TypeSpecies	Helotiaceae (2)	Helotiaceae	
EU019300	Trimmatostroma salicis	CPC 13571	TypeSpecies	Mollisiaceae	Mollisiaceae	
LT158459	Trochila ilicina	BPI 877264		Cenangiaceae	Cenangiaceae	
LT158460	Trochila laurocerasi	BPI 879818		Cenangiaceae	Cenangiaceae	
KY657581	Tromeropsis microtheca	Baral HB10054	TypeSpecies	Leotiomycetes incertae sedis	Leotiomycetes incertae sedis	
FN868463	Tryblidiopsis pinastri	BLE11	TypeSpecies	Rhytismataceae	Rhytismataceae	
JF793678	Tryblidiopsis pinastri	CBS 445.71	TypeSpecies	Rhytismataceae	Rhytismataceae	
LQH8c	Tympanis confusa	Farlow (LQH8c)		Tympanidaceae	Tympanidaceae	GenBank MH810146
AA1a	Tympanis tsugae	Farlow (AA1a)		Tympanidaceae	Tympanidaceae	GenBank MH810147
HG326612	Unguicularia unguiculata	NK322	TypeSpecies	Hyaloscyphaceae	Helotiales incertae sedis	
JN033443	Urceolella carestiana	TNS-F-18014		Hyaloscyphaceae	Stamnaria lineage/Han Clade 9	
JN033379	Urceolella crispula	KACC 45486	Type Species	Hyaloscyphaceae	Stamnaria lineage/Han Clade 9	
KF212190	Valdensinia heterodoxa	Val5	Type Species	Sclerotiniaceae	Sclerotiniaceae	
LT904725	Vandijckella johannae	CBS 143182	exTypeSpecimen	Vandijckellaceae (2)	Vandijckellaceae	
CCM_F19494	'Varicosporium' delicatum	CCM F-19494, CBS 574.95				GenBank JQ412864
DQ202517	Varicosporium elodeae	CBS 541.92	TypeSpecies	Helotiaceae (2)	Discinella-Pezoloma lineage	
CCM_F04276	Varicosporium elodeae	CCM F-04276	TypeSpecies	Helotiaceae (2)	Discinella-Pezoloma lineage	GenBank MK226459
NR_111206	Variocladium giganteum	CBS 508.71		Helotiaceae (2)	Mollisiaceae	
LT158421	Velutarina alpestris	Baral HB8252a		Cenangiaceae	Cenangiaceae	
JN033447	Venturiocistella japonica	TNS-F-18030		Hyaloscyphaceae	Helotiales - Han Clade 4	
VCU21826	Verpatinia calthicola	LMK448	TypeSpecies	Sclerotiniaceae	Sclerotiniaceae	
EU434854	Vibrissea truncorum	AFTOL-ID 1322	TypeSpecies	Vibrisseaceae	Vibrisseaceae	
EU434855	Vibrissea truncorum	CBS 143.92	TypeSpecies	Vibrisseaceae	Vibrisseaceae	
KF429257	Vibrissea wood in stream	ICMP 19442		Vibrisseaceae	Vibrisseaceae	
HQ599580	Xenopolyscytalum pinea	CPC 14225	exTypeSpecimen	Helotiales incertae sedis (2)	Pezizellaceae	

Genbank accession or code	Species	Voucher	Type status of specimen (3)	Family (1, 2)	Accepted family	Notes
JX454953	Xerombrophila crystallifera	CBS 128289	exTypeSpecimen	Gelatinodiscaceae	Gelatinodiscaceae	
LT158441	Xeropilidium dennisii	TU 104501	exTypeSpecimen	Chaetomellaceae	Chaetomellaceae	
GQ272624	Xylogone sphaerospora	ATCC 34392	exTypeSpecimen	Helotiales incertae sedis (2)	Helotiales incertae sedis	
