# Supplementary material for: A multigene phylogeny toward a new phylogenetic classification of Leotiomycetes
Source: IMA Fungus. 2019 Jun 7;10:1. doi: 10.1186/s43008-019-0002-x (PMC7325659; doi:10.1186/s43008-019-0002-x)
Supplement: Supplementary file 3 — Figure S1. PhyDesign analysis. The upper image depicts the ultrametric tree for the taxa sampled using the single-copy orthologue dataset of Rokas et al. (2005) as modified by Taylor and Berbee (2006). Calibration points targeted the divergence of major Pezizomycotina lineages, approximately 250–400 million years ago, based on the fungal, animal and plant calibrations of Taylor and Berbee (2006). The lower image displays phylogenetic informativeness profiles for gene fragments from the datasets of Rokas et al. (2005), Taylor and Berbee (2006) and the AFTOL-1 project (Schoch et al. 2009). AFTOL-1 gene fragments are labeled with an asterisk (*). The five lesser-known fragments predicted to provide the greatest phylogenetic nformativeness for the desired epoch are also labeled. (PDF 456 kb) [file 43008_2019_2_MOESM3_ESM.pdf]

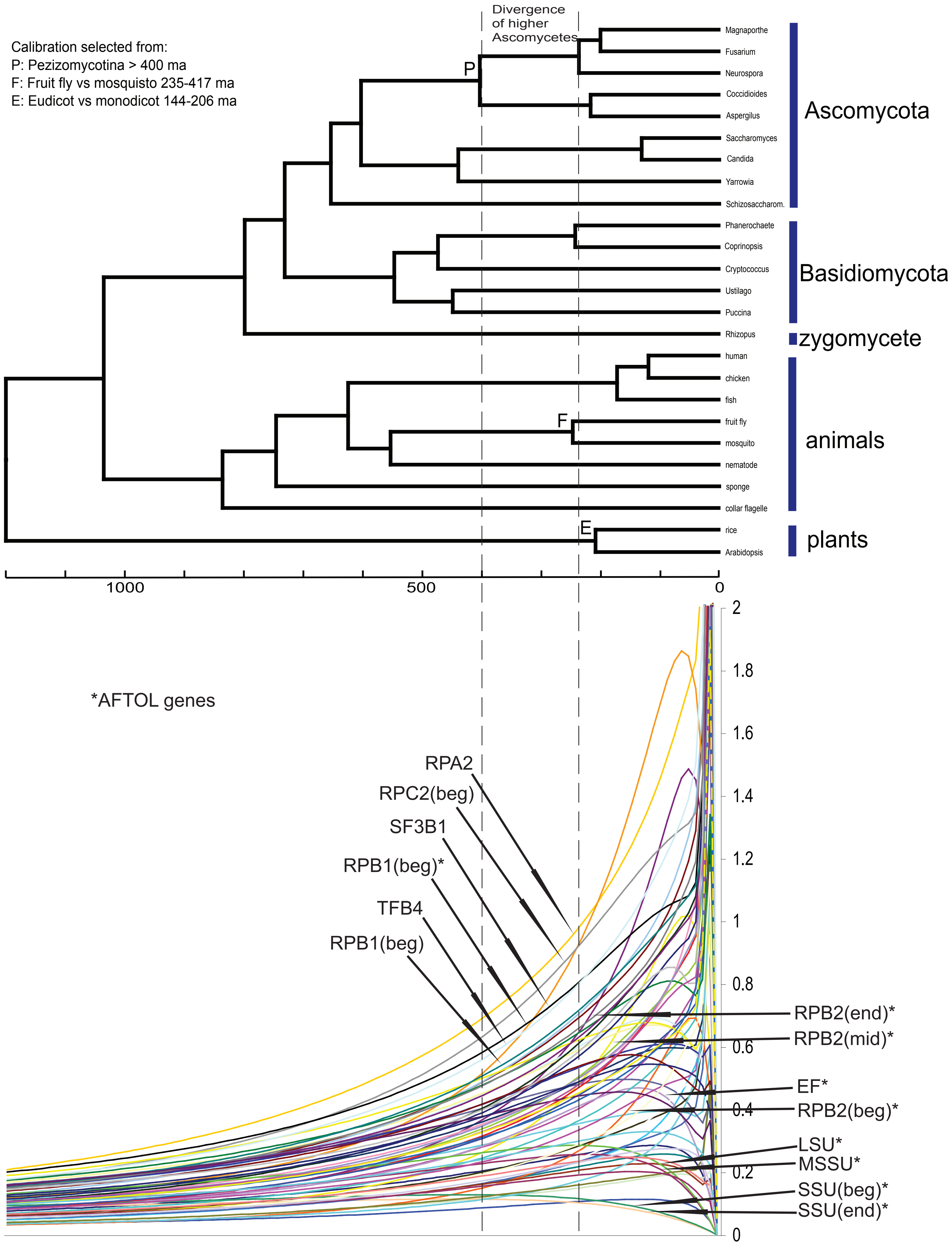

Divergence of higher Ascomycetes
Calibration selected from:
P: Pezizomycotina > 400 ma
F: Fruit fly vs mosquisto 235-417 ma
E: Eudicot vs monodicot 144-206 ma
P
F
E
Magnaporthe
Fusarium
Neurospora
Coccidioides
Aspergilus
Saccharomyces
Candida
Yarrowia
Schizosaccharom.
Phanerochaete
Coprinopsis
Cryptococcus
Ustilago
Puccina
Rhizopus
human
chicken
fish
fruit fly
mosquito
nematode
sponge
collar flagelle
rice
Arabidopsis
Ascomycota
Basidiomycota
zygomycete
animals
plants
1000
500
0
*AFTOL genes
RPA2
RPC2(beg)
SF3B1
RPB1(beg)*
TFB4
RPB1(beg)
RPB2(end)*
RPB2(mid)*
EF*
RPB2(beg)*
LSU*
MSSU*
SSU(beg)*
SSU(end)*
2
1.8
1.6
1.4
1.2
1
0.8
0.6
0.4
0.2
0
