# Supplementary material for: A multigene phylogeny toward a new phylogenetic classification of Leotiomycetes
Source: IMA Fungus. 2019 Jun 7;10:1. doi: 10.1186/s43008-019-0002-x (PMC7325659; doi:10.1186/s43008-019-0002-x)
Supplement: Supplementary file 4 — Table S3. The top 10 loci estimated for effectively resolving major nodes basal in the Leotiomycetes phylogeny based on the PhyDesign analysis (Additonal file 3: Figure S1). Those used in our analyses marked with *. (DOCX 13 kb) [file 43008_2019_2_MOESM4_ESM.docx]

**Additional file 4: Table S3.** The top 10 loci estimated for effectively resolving major nodes basal in the *Leotiomycetes* phylogeny. Those used in our analyses marked with *

| **Locus** | **length**  **(bp)** | **Phylogenetic**  **informativeness** | **Forward**  **(5’->3’)** | **Reverse**  **(5’->3’)** |
| --- | --- | --- | --- | --- |
| RPA2* | 1452 | 134.9 | TNTGYCCNGTNCAYACNCC | CRTCNCKYTCCATYTCNCC |
| RPC2* | 1164 | 125.6 | RGGNYTNGTNAARCARCA | DAYNCKNGTCATNCC |
| RPB1* | 1101 | 113.3 | GARTGYCCDGGDCAYTTYGG | TTCATYTCRTCDCCRTCRAARTC |
| SF3B1* | 1263 | 112.6 | GMARGCNMGNCAYACNGG | TCRTGNGGNCCDATNGCYT |
| TFB4* | 1227 | 109.8 | GAYGARGCNCAYAAYATHG | YRTGNCKCATNGCRTCRAA |
| RPA1* | 921 | 96.6 | GCNGAYTTYGAYGGNGAYG | CKNCCNGCCATRSWRTGRA |
| SNF2 | 1026 | 95.6 | SYGAYGAYATGGGNYTNGG | CKRTCCATNGYCTGNARRTC |
| HSP70-C | 1245 | 92.2 | YCARGTNGCNATGAAYCC | NCCRTTNGCRTCDAYRTC |
| SWI/SNF | 1089 | 92.0 | SYGAYGAYATGGGNYTNGG | CKRTCCATNGCYTGNARRTC |
| α-tubulin* | 1149 | 91.2 | RGTNGGNAAYGCNTGYTGGGA | CCATNCCYTCNCCNACRTACCA |
