# Supplementary material for: A multigene phylogeny toward a new phylogenetic classification of Leotiomycetes
Source: IMA Fungus. 2019 Jun 7;10:1. doi: 10.1186/s43008-019-0002-x (PMC7325659; doi:10.1186/s43008-019-0002-x)
Supplement: Supplementary file 5 — Table S7. Depth of gene coverage across the specimens sampled. (DOCX 12 kb) [file 43008_2019_2_MOESM5_ESM.docx]

**Additional file 5: Table S7.** Depth of gene coverage across the specimens sampled.

| **Number of genes** | **Number of specimens** |
| --- | --- |
| 15 | 30 |
| >10 | 67 |
| >5 | 142 |
| 4 | 39 |
| 3 | 34 |
| 2 | 5 |
