# Supplementary figures and images for: A multigene phylogeny toward a new phylogenetic classification of Leotiomycetes
Source: IMA Fungus. 2019 Jun 7;10:1. doi: 10.1186/s43008-019-0002-x (PMC7325659; doi:10.1186/s43008-019-0002-x)

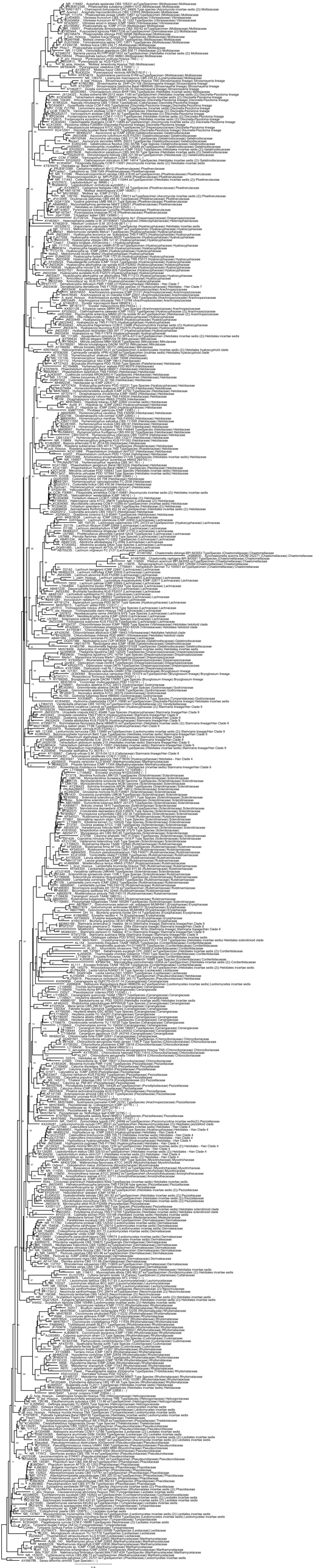

Supplement: Supplementary file 6 — Figure S2. ML tree based on ITS sequences. Labels include Genbank accession number, voucher number, family name accepted by Baral (2016) in brackets (as “( - )” if not treated in that work), and family name accepted on the basis of the 15-gene (Figs. 2, 3, 4, 5 and 6) and ITS phylogenies. Bootstrap values > 50% are indicated. The phylogenetic tree is rooted with Sarea and Tiarosporella. The alignment used for this analysis and a nexus version of the tree can be downloaded from the Manaaki Whenua – Landcare Research datastore, see https://doi.org/10.7931/T5YV-BE95. (PDF 7796 kb) [file 43008_2019_2_MOESM6_ESM.pdf]
