# Supplementary material for: A multigene phylogeny toward a new phylogenetic classification of Leotiomycetes
Source: IMA Fungus. 2019 Jun 7;10:1. doi: 10.1186/s43008-019-0002-x (PMC7325659; doi:10.1186/s43008-019-0002-x)
Supplement: Supplementary file 7 — Table S6. Summary of the family level relationships of the genera treated, comparing the taxonomy of Baral (2016) with the relationships suggested in our analyses (genera where higher taxon has changed are in bold). Where a genus was not treated by Baral (2016) the family level classification provided by Index Fungorum in provided. Also provided is the type status of the specimens used in our analyses, and where the suggested relationship is based on the ITS analysis only. (PDF 566 kb) [file 43008_2019_2_MOESM7_ESM.pdf]

Suppl Data Table S6. Summary of the family level relationships of the genera treated, comparing the taxonomy of Baral (2016) with the relationships suggested in our analyses (genera where higher taxon has changed are in bold). Where a genus was not treated by Baral (2016) the family level classification provided by Index Fungorum in provided. Also provided is the type status of the specimens used in our analyses, and where the suggested relationship is based on the ITS analysis only.

(1) based on Baral (2016) unless otherwise stated
(2) based on Index Fungorum, accessed 3 September 2018, http://www.indexfungorum.org/names/names.asp
(3) based on the ITS gene tree only
(4) type material with ITS data only

genus	current family (1, 2)	accepted family (3)	type status of specimens sequenced (4)	note
Acephala	Mollisiaceae	Mollisiaceae	exTypeSpecimen	
Acidea	Discinella-Pezoloma lineage	Discinella-Pezoloma lineage (3)	exTypeSpecimen	
Acidomelania	Mollisiaceae	Mollisiaceae (3)	exTypeSpecimen	
**Alatospora**	**Leotiaceae (2)**	**Leotiales incertae sedis**	**TypeSpecies**	
Albotricha	Lachnaceae	Lachnaceae	TypeSpecies	
Allantophomopsiella	Phacidiaceae	Phacidiaceae (3)	ExTypeSpecimen	
Allantophomopsis	Phacidiaceae	Phacidiaceae (3)	exTypeSpecimen	
Ameghiniella	Cordieritidacaeae	Cordieritidacaeae	TypeSpecies	
Amicodisca	Hyaloscyphaceae	Hyaloscyphaceae (3)	TypeSpecies (4)	A. castaneae in the 15 gene tree is distant to the type species A. virella in the ITS tree.
Amorphotheca	Amorphothecaceae	Amorphothecaceae	exTypeSpecimen (4)	
Amylocarpus	Helotiaceae	Helotiaceae (3)	TypeSpecies	closely related to 'Hymenoscphus' repandus CBS 341.76 in ITS tree
Antarctomyces	Thelebolaceae	Thelebolaceae (3)	exTypeSpecimen	
**Aotearoamyces**	**Tympanidaceae**	**Leotiales incertae sedis**	**TypeSpecies**	
Aotearoamyces	Tympanidaceae	Leotiales incertae sedis	TypeSpecies	
Aquapoterium	Helotiales incertae sedis (2)	Helotiales incertae sedis (3)	exTypeSpecimen	
Arachnopeziza	Arachnopezizaceae	Arachnopezizaceae	TypeSpecies	
**Arbusculina**	**Pezizomycotina incertae sedis (2)**	**Hyaloscyphaceae (3)**		
Articulospora	Discinella-Pezoloma lineage	Discinella-Pezoloma lineage	TypeSpecies	
Ascocalyx	Godroniaceae	Godroniaceae (3)		
Ascocoryne	Gelatinodiscaceae	Gelatinodiscaceae	TypeSpecies	
Ascotremella	Gelatinodiscaceae	Gelatinodiscaceae (3)	TypeSpecies	
**Asperopilum**	**Hyaloscyphaceae**	**Lachnaceae (3)**	**TypeSpecies**	
Asterocalyx	Helotiales incertae sedis	Helotiales incertae sedis (3)	TypeSpecies	
**Austropezia**	**Arachnopezizaceae**	**Pezizellaceae (3)**	**TypeSpecies**	
Banksiamyces	incertae sedis	Helotiales incertae sedis (3)		
**Barrenia**	**Helotiales incertae sedis (2)**	**Mollisiaceae (3)**	**exTypeSpecimen**	
Belonioscyphella	Helotiales incertae sedis	Helotiales (Stamnaria lineage/Han Clade 9) (3)	TypeSpecies	
Bicornispora	Rutstroemiaceae	Rutstroemiaceae (3)	TypeSpecies	
Bisporella	Helotiaceae	Helotiaceae (3)		based on Bispora, anamorph of Bisporella
Bivallum	Rhytismataceae	Rhytismataceae (3)		
Bloxamia	Pezizellaceae	Pezizellaceae (3)	TypeSpecies	
Blumeria	Erysiphaceae	Erysiphaceae	TypeSpecies	
Blumeriella	Drepanopezizaceae	Drepanopezizaceae (3)		
Botrytis	Sclerotiniaceae	Sclerotiniaceae	TypeSpecies	
**Brunaudia**	**Patellariaceae (2)**	**Helotiaceae (3)**	**TypeSpecies**	
Brunnipila	Lachnaceae	Lachnaceae		
Bryoclaviculus	Bryoglossum lineage	Bryoglossum lineage (3)	exTypeSpecimen	
Bryoglossum	Bryoglossum lineage	Bryoglossum lineage (3)	TypeSpecies	
Bryoscyphus	Helotiaceae	Helotiaceae		
Bulgaria	Phacidiaceae	Phacidiaceae	TypeSpecies	
**Bulgariella**	**Helotiales incertae sedis**	**Helotiaceae**	**TypeSpecies**	
Byssoascus	Myxotrichaceae	Myxotrichaceae (3)	exTypeSpecimen	
Cadophora	Ploettnerulaceae	Ploettnerulaceae	TypeSpecies (4)	
Cairneyella	Helotiales incertae sedis (2)	Helotiales incertae sedis (hyaloscyphoid clade)	exTypeSpecimen	
Calloria	Calloriaceae	Helotiales (Stamnaria lineage/Han Clade 9) (3)		
Calycellina	Pezizellaceae	Pezizellaceae (3)		
**Calycellinopsis**	**Dermateaceae (2)**	**Cenangiaceae**	**TypeSpecies**	
Calycina	Pezizellaceae	Pezizellaceae		
Capitotricha	Lachnaceae	Lachnaceae (3)	TypeSpecies	
**Cashiella**	**Helotiales incertae sedis**	**Helotiales incertae sedis (3)**		
Catenulifera	Helotiales incertae sedis (2)	Helotiales (Han Clade 4) (3)		
Cenangiopsis	Cenangiaceae	Cenangiaceae	TypeSpecies	
Cenangium	Cenangiaceae	Cenangiaceae	TypeSpecies	
Chaetomella	Chaetomellaceae	Chaetomellaceae	TypeSpecies	
Chaetoscypha	Helotiaceae	Helotiaceae		as "Pirottaea"
**Chalara**	**Pezizellaceae**	**Hyaloscyphaceae**	**TypeSpecies**	
**Cheirospora**	**Ascomycota incertae sedis (2)**	**Mollisiaceae (3)**	**TypeSpecies**	
Chlorencoelia	Cenangiaceae	Cenangiaceae	TypeSpecies	
Chlorociboria	Chlorociboriaceae	Chlorociboriaceae	TypeSpecies	
Chloroscypha	Gelatinodiscaceae	Gelatinodiscaceae		
**Chlorosplenium**	**Helotiales incertae sedis**	**Mollisiaceae**	**TypeSpecies**	
**Chlorovibrissea**	**Vibrisseaceae**	**Helotiales incertae sedis (helotioid clade)**	**TypeSpecies**	
Ciboria	Sclerotiniaceae	Sclerotiniaceae		
Ciborinia	Sclerotiniaceae	Sclerotiniaceae	TypeSpecies	
**Cistella**	**Hyaloscyphaceae**	**Helotiales (Stamnaria lineage/Han Clade 9)**		
**Cladochasiella**	**Ascomycota incertae sedis (2)**	**Discinella-Pezoloma lineage (3)**	**exTypeSpecimen**	
Clarireedia	Rutstroemiaceae	Rutstroemiaceae	TypeSpecies	
**Clathrosphaerina**	**Hyaloscyphaceae (2)**	**Helotiales incertae sedis (3)**	**TypeSpecies**	**sister to Arachnopezizaceae in ITS tree, but support needs confirming with additional genes; ITS sequence used matches that from ex type material (CBS 162.49, GenBank NR_159766)**
**Clathrosporium**	**Helotiales incertae sedis (2)**	**Gelatinodiscaceae (3)**	**TypeSpecies**	
**Claussenomyces**	**Tympanidaceae**	**Leotiales incertae sedis**		
Cleistothelebolus	Thelebolaceae	Thelebolaceae (3)	TypeSpecies	
Coccomyces	Rhytismataceae	Rhytismataceae		
Cochlearomyces	Cochlearomycetaceae (2)	Cochlearomycetaceae (3)	ExTypeSpecimen	cf Leotiales
**Coleophoma**	**Leotiomycetes incertae sedis (2)**	**Dermateaceae (3)**		
**Collembolispora**	**Helotiales incertae sedis (2)**	**Ploettnerulaceae (3)**	**exTypeSpecimen**	
Collophorina	Tympanidaceae	Tympanidaceae	exTypeSpecimen	
Colpoma	Rhytismataceae	Rhytismataceae (3)	TypeSpecies	
**Connersia**	**Pseudeurotiaceae**	**Helotiaceae**	**exTypeSpecimen**	
Coprotinia	Sclerotiniaceae	Sclerotiniaceae (3)	TypeSpecies	
Cordierites	Cordieritidacaeae	Cordieritidacaeae	TypeSpecies	
Cristulariella	Sclerotiniaceae	Sclerotiniaceae (3)	TypeSpecies	
Crocicreas	Helotiales incertae sedis	Helotiales incertae sedis (pezizelloid clade)	TypeSpecies	
Crumenulopsis	Cenangiaceae	Cenangiaceae (3)	TypeSpecies	
**Cryptohymenium**	**Dermateaceae (2)**	**Helotiales incertae sedis (sclerotinioid clade)**	**TypeSpecies**	
Cryptosporiopsis	Dermateaceae	Dermateaceae	TypeSpecies	
Cudonia	Cudoniaceae	Cudoniaceae (3)	TypeSpecies	
Cudoniella	Helotiaceae	Helotiaceae		"Hymenoscpyhus" varicosporioides clade
**Curviclavula**	**Helotiales incertae sedis (2)**	**Pezizellaceae (3)**	**exTypeSpecimen**	**Han Clade 1 subclade**

genus	current family (1, 2)	accepted family (3)	type status of specimens sequenced (4)	note
Cyathicula	Helotiaceae	Helotiaceae (3)	TypeSpecies	
Cyclaneusma	Marthamycetaceae	Marthamycetaceae	exTypeSpecimen	
Cyttaria	Cyttariaceae	Cyttariaceae	TypeSpecies	
Darkera	Phacidiaceae	Phacidiaceae (3)	exTypeSpecimen	
Dasyscyphella	Lachnaceae	Lachnaceae		
Davidhawksworthia	Dermateaceae (2)	Dermateaceae (3)	exTypeSpecimen	
Deltopyxis	Leotiomycetes incertae sedis	Leotiomycetes incertae sedis (3)	exTypeSpecimen	
**Dematioscypha**	**Hyaloscyphaceae**	**Helotiales (Han Clade 7)**	**TypeSpecies**	
Dermea	Dermateaceae	Dermateaceae		
Dicephalospora	Helotiaceae	Helotiaceae		
**Dimorphospora**	**Helotiaceae (2)**	**Gelatinodiscaceae (3)**	**exTypeSpecimen**	
Diplocarpa	Cordieritidacaeae	Cordieritidacaeae		
Diplocarpon	Drepanopezizaceae	Drepanopezizaceae	TypeSpecies	
Diplolaeviopsis	Cordieritidacaeae	Cordieritidacaeae (3)		
Discinella	Discinella-Pezoloma lineage	Discinella-Pezoloma lineage (3)	TypeSpecies	
Duebenia	Calloriaceae	Helotiales (Stamnaria lineage/Han Clade 9) (3)		
Dumontinia	Sclerotiniaceae	Sclerotiniaceae (3)	TypeSpecies	
Durella	Strossmayeria lineage	Strossmayeria lineage (3)		Durella connivens
Eleutheromyces	Helicogoniaceae	Helicogoniaceae (3)	exTypeSpecimen	
Elliottinia	Sclerotiniaceae	Sclerotiniaceae (3)	TypeSpecies	
Elytroderma	Rhytismataceae	Rhytismataceae (3)	TypeSpecies	
Encoelia	Cenangiaceae	Cenangiaceae	TypeSpecies	
**Endoscypha**	**Hyaloscyphaceae**	**Helotiaceae (3)**	**TypeSpecies**	
**Epicladonia**	**Ascomycota incertae sedis (2)**	**Phacidiaceae (3)**	**TypeSpecies**	
Epiglia	Mniaecia lineage	Mniaeciaceae	TypeSpecies	
Eriopezia	Arachnopezizaceae	Arachnopezizaceae (3)	TypeSpecies	
Erysiphe	Erysiphaceae	Erysiphaceae		
Fabrella	Cenangiaceae	Cenangiaceae (3)	TypeSpecies	
Filosporella	Ascomycota incertae sedis (2)	Helotiales incertae sedis		type not sequenced, other species genetically divergent
Flagellospora	Nectriaceae (2)	Leotiales incertae sedis	TypeSpecies	
**Fontanospora**	**Helotiales incertae sedis (2)**	**Discinella-Pezoloma lineage (3)**	**TypeSpecies**	
**Fulvoflamma**	**Rhytismatales incertae sedis (2)**	**Phacidiaceae (3)**	**exTypeSpecimen**	
**FuscoscIera**	**Dermateaceae (2)**	**Mollisiaceae (3)**	**exTypeSpecimen**	
**Gamarada**	**Hyaloscyphaceae (2)**	**Helotiales (Han Clade 4)**	**TypeSpecies**	
Gelatinipulvinella	Helicogoniaceae	Helicogoniaceae	TypeSpecies	
Gelatinodiscus	Gelatinodiscaceae	Gelatinodiscaceae (3)	TypeSpecies	
Gelatinomyces	Tympanidaceae	Tympanidaceae (3)	exTypeSpecimen	
Gelatinopsis	Helicogoniaceae	Helicogoniaceae		
Geltingia	Helicogoniaceae	Helicogoniaceae (3)	TypeSpecies	
Gemmina	Pezizellaceae	Pezizellaceae (3)	TypeSpecies	Han Clade 1 subclade
**Geomyces**	**Myxotrichaceae**	**Pseudeurotiaceae (3)**	**exTypeSpecimen**	
Glarea	Helotiaceae	Helotiaceae	exTypeSpecimen	
**Gloeotinia**	**Helotiaceae**	**Helotiales incertae sedis (3)**	**TypeSpecies**	**ITS close to Cyttaria and Myxocephala**
Godronia	Godroniaceae	Godroniaceae		
Golovinomyces	Erysiphaceae	Erysiphaceae		
**Gorgomyces**	**Ascomycota incertae sedis (2)**	**Leotiales incertae sedis (3)**	**exTypeSpecimen**	
Graddonia	Helotiaceae	Helotiaceae (3)		"Hymenoscpyhus" varicosporioides clade
Gremmeniella	Godroniaceae	Godroniaceae	TypeSpecies	
**Grovesiella**	**Tympanidaceae**	**Godroniaceae (3)**	**TypeSpecies**	
Grovesinia	Sclerotiniaceae	Sclerotiniaceae (3)	TypeSpecies	
**Gymnostellatospora**	**Myxotrichaceae (2)**	**Pseudeurotiaceae (3)**		
Gyoerffyella	Discinella-Pezoloma lineage	Discinella-Pezoloma lineage		
**Halenospora**	**Leotiaceae (2)**	**Helotiaceae**	**TypeSpecies**	**"Hymenoscpyhus" varicosporioides clade; = Zalerion varium**
**Hamatocanthoscypha**	**Pezizellaceae**	**Pezizellaceae**	**TypeSpecies**	
Haradamyces	Sclerotiniaceae	Sclerotiniaceae (3)	TypeSpecies	
Helicocentralis	Leotiomycetes incertae sedis (2)	Helotiales incertae sedis (hyaloscyphoid clade) (3)	exTypeSpecimen	sister to Cairneyella
**Helicodendron**	**Helotiales incertae sedis (2)**	**Gelatinodiscaceae (3)**	**TypeSpecies**	
Heterosphaeria	Heterosphaeriaceae	Heterosphaeriaceae (3)	TypeSpecies	
Heyderia	Cenangiaceae	Cenangiaceae	TypeSpecies	
**Hispidula**	**Hyaloscyphaceae**	**Helotiaceae**		
**Holwaya**	**Tympanidaceae**	**Leotiomycetes incertae sedis**	**TypeSpecies**	
**Hyalodendriella**	**Helotiales incertae sedis (2)**	**Pezizellaceae (3)**	**exTypeSpecimen**	**Han Clade 1 subclade**
Hyalopeziza	Hyaloscyphaceae	Hyaloscyphaceae		
Hyaloscypha	Hyaloscyphaceae	Hyaloscyphaceae	TypeSpecies	
**Hydrocina**	**Discinella-Pezoloma lineage**	**Helotiales incertae sedis (3)**	**exTypeSpecimen**	
Hymenoscyphus	Helotiaceae	Helotiaceae	TypeSpecies	
Hymenotorrendiella	Helotiaceae	Helotiaceae	TypeSpecies (4)	
**Hyphodiscus**	**Hyaloscyphaceae**	**Helotiales (Han Clade 4)**	**TypeSpecies (4)**	
**Hyphopeziza**	**Hyaloscyphaceae**	**Helotiales (Han Clade 4)**	**TypeSpecies (4)**	
Hypoderma	Rhytismataceae	Rhytismataceae	TypeSpecies	
Incrucipulum	Lachnaceae	Lachnaceae	TypeSpecies (4)	
**Infundichalara**	**incertae sedis**	**Pezizellaceae**	**exTypeSpecimen**	
Ionomidotis	Cordieritidacaeae	Cordieritidacaeae	TypeSpecies	
Kohninia	Sclerotiniaceae	Sclerotiniaceae (3)	TypeSpecies	
Lachnellula	Lachnaceae	Lachnaceae		
**Lachnopsis**	**Hyaloscyphaceae (2)**	**Lachnaceae (3)**	**exTypeSpecimen**	
Lachnum	Lachnaceae	Lachnaceae		
Laetinaevia	Calloriaceae	Helotiales (Stamnaria lineage/Han Clade 9) (3)		
Lambertella	Rutstroemiaceae	Rutstroemiaceae	TypeSpecies	
Lanzia	Rutstroemiaceae	Rutstroemiaceae		
Lareunionomyces	Neolauriomycetaceae (2)	Neolauriomycetaceae (3)	exTypeSpecimen	
Lasiobelonium	Lachnaceae	Lachnaceae		
**Lemonniera**	**Helotiales incertae sedis (2)**	**Discinella-Pezoloma lineage**	**TypeSpecies**	
Leohumicola	Leotiomycetes incertae sedis (2)	Helotiales (Stamnaria lineage/Han Clade 9) (3)	exTypeSpecimen	
Leotia	Leotiaceae	Leotiaceae	TypeSpecies	
Leptodontidium	Helotiales incertae sedis (2)	Helotiales (Han Clade 4) (3)	exTypeSpecimen	based on L. elatius ex ITS tree;
Leuconeurospora	Pseudeurotiaceae	Pseudeurotiaceae	exTypeSpecimen	
Llimoniella	Cordieritidacaeae	Cordieritidacaeae		
Lophodermium	Rhytismataceae	Rhytismataceae (3)		
**Loramyces**	**Loramycetaceae**	**Mollisiaceae**	**TypeSpecies**	
Macroskyttea	Helotiales incertae sedis (2)	Cordieritidacaeae (3)	exTypeSpecimen	
Malbranchea	Myxotrichaceae	Myxotrichaceae (3)		
**Margaritispora**	**Helotiales incertae sedis (2)**	**Discinella-Pezoloma lineage**	**TypeSpecies**	**based on LSU phylogeny using DQ267635 (unpubl. data)**
Marssonina	Drepanopezizaceae	Drepanopezizaceae		
Marthamyces	Marthamycetaceae	Marthamycetaceae	TypeSpecies	
**Mastigosporium**	**Ascomycota incertae sedis (2)**	**Ploettnerulaceae (3)**	**exTypeSpecimen**	
Medeolaria	Medeolariaceae	Medeolariaceae (3)	TypeSpecies	cf Dermateaceae but on long branch
Meliniomyces	Hyaloscyphaceae	Hyaloscyphaceae	TypeSpecies	= Hyaloscypha, Fehrer et al. 2019
Meloderma	Rhytismataceae	Rhytismataceae (3)	TypeSpecies	
Meria	Cenangiaceae	Cenangiaceae	TypeSpecies	
Microglossum	Leotiaceae	Leotiaceae	TypeSpecies	
Microscypha	Pezizellaceae	Pezizellaceae		
**Miniancora**	**Ascomycota incertae sedis (2)**	**Leotiales incertae sedis (3)**	**exTypeSpecimen**	

genus	current family (1, 2)	accepted family (3)	type status of specimens sequenced (4)	note
Mitrula	Mitrulaceae	Mitrulaceae (3)	TypeSpecies	
**Mitrulinia**	**Helotiales incertae sedis**	**Helotiaceae (3)**	**TypeSpecies**	
Mniaecia	Mniaecia lineage	Mniaeciaceae	TypeSpecies	
Moellerodiscus	Rutstroemiaceae	Rutstroemiaceae		
Mollisia	Mollisiaceae	Mollisiaceae	TypeSpecies	
Mollisina	Pezizellaceae	Pezizellaceae		
Monilinia	Sclerotiniaceae	Sclerotiniaceae (3)	TypeSpecies	
**Mycoarthris**	**Hyaloscyphaceae (2)**	**Helotiales (Stamnaria lineage/Han Clade 9) (3)**	**exTypeSpecimen**	
Mycochaetophora	Ploettnerulaceae	Ploettnerulaceae (3)		
**Mycofalcella**	**Ascomycota incertae sedis (2)**	**Helotiaceae**	**exTypeSpecimen**	**"Hymenoscpyhus" varicosporioides clade**
Mycopappus	Sclerotiniaceae	Sclerotiniaceae (3)	TypeSpecies	
Mycosymbioces	Leotiomycetes incertae sedis (2)	Leotiales incertae sedis (3)	exTypeSpecimen	
Myriodiscus	Tympanidaceae	Tympanidaceae	TypeSpecies	
Myriosclerotinia	Sclerotiniaceae	Sclerotiniaceae	TypeSpecies	
**Myxocephala**	**Sordariomycetes (2)**	**Helotiales incertae sedis (3)**	**exTypeSpecimen**	**ITS sister to Cyttaria**
Myxotrichum	Myxotrichaceae	Myxotrichaceae (3)	TypeSpecies	
Naemacyclus	Marthamycetaceae	Marthamycetaceae	TypeSpecies	
**Naevala**	**Calloriaceae**	**Discinella-Pezoloma lineage (3)**	**TypeSpecies**	
Neobulgaria	Gelatinodiscaceae	Gelatinodiscaceae	TypeSpecies	
Neocrinula	Neocrinulaceae (2)	Neocrinulaceae (3)	exTypeSpecimen	
Neocudoniella	Gelatinodiscaceae	Bryoglossum lineage (3)	exTypeSpecimen	
Neodasyscypha	Lachnaceae	Lachnaceae (3)	TypeSpecies	
Neofabraea	Dermateaceae	Dermateaceae	exTypeSpecimen	
Nervostroma	Sclerotiniaceae	Sclerotiniaceae (3)	exTypeSpecimen	
Oculimacula	Ploettnerulaceae	Ploettnerulaceae (3)	TypeSpecies	
Oidiodendron	Myxotrichaceae	Myxotrichaceae	TypeSpecies (4)	
Olla	Hyaloscyphaceae	Hyaloscyphaceae		
Orbiliopsis	Helotiales incertae sedis	Helotiales incertae sedis (3)		
Ovulinia	Sclerotiniaceae	Sclerotiniaceae (3)	TypeSpecies	
Pachycudonia	Cudoniaceae	Cudoniaceae (3)	TypeSpecies	
Parafabraea	Dermateaceae	Dermateaceae	exTypeSpecimen	
Patinella	Helotiales incertae sedis	Leotiomycetes incertae sedis (3)	TypeSpecies	sister to Holwaya
Perrotia	Lachnaceae	Lachnaceae (3)	TypeSpecies	
Pezicula	Dermateaceae	Dermateaceae	exTypeSpecimen	
Pezoloma	Discinella-Pezoloma lineage	Discinella-Pezoloma lineage		
Phacidium	Phacidiaceae	Phacidiaceae	exTypeSpecimen (4)	
Phaeohelotium	Helotiaceae	Helotiaceae		
Phialina	Pezizellaceae	Pezizellaceae		
Phialocephala	Mollisiaceae	Mollisiaceae	exTypeSpecimen	
Phlyctema	Dermateaceae	Dermateaceae	TypeSpecies	
Piceomphale	Sclerotiniaceae	Rutstroemiaceae	TypeSpecies	
Pilidium	Chaetomellaceae	Chaetomellaceae	exTypeSpecimen	
**Pleuroascus**	**Pseudeurotiaceae**	**Helotiaceae**	**TypeSpecies**	
Poculum	Rutstroemiaceae	Rutstroemiaceae		
Polydesmia	Helotiales incertae sedis	Helotiales incertae sedis (sclerotinioid clade)	TypeSpecies	
Polyphilus	Hyaloscyphaceae	Helotiales (Stamnaria lineage/Han Clade 9)	exTypeSpecimen	
Porodiplodia	Porodiplodiaceae	Pezizellaceae (3)	exTypeSpecimen	
Potebniamyces	Phacidiaceae	Phacidiaceae	TypeSpecies	
Proliferodiscus	Lachnaceae	Lachnaceae	TypeSpecies (4)	
Propolis	Marthamycetaceae	Marthamycetaceae		
Pseudaegerita	Hyaloscyphaceae	Hyaloscyphaceae (3)	TypeSpecies	= Hyaloscypha, Fehrer et al. 2019
Pseudeurotium	Pseudeurotiaceae	Pseudeurotiaceae	exTypeSpecimen	
Pseudofabraea	Dermateaceae	Dermateaceae	exTypeSpecimen	
**Pseudographis**	**Triblidiaceae**	**Rhytismataceae**	**TypeSpecies**	
Pseudogymnoascus	Pseudeurotiaceae	Pseudeurotiaceae	TypeSpecies	
**Pseudopezicula**	**Drepanopezizaceae**	**Discinella-Pezoloma lineage (3)**	**TypeSpecies**	
Pseudopeziza	Drepanopezizaceae	Drepanopezizaceae (3)		
Pseudophacidium	Phacidiaceae	Phacidiaceae (3)	TypeSpecies	
**Psilachnum**	**Pezizellaceae**	**Helotiales (Stamnaria lineage/Han Clade 9)**		
**Psychrophila**	**Helotiaceae (2)**	**Helotiales incertae sedis (3)**	**exTypeSpecimen**	**sister to Arachnopezizaceae in ITS tree, but support needs confirming with additional genes**
Pycnopeziza	Sclerotiniaceae	Sclerotiniaceae (3)	TypeSpecies	
Pyrenopeziza	Ploettnerulaceae	Ploettnerulaceae (3)		
Rhabdocline	Cenangiaceae	Cenangiaceae (3)	TypeSpecies	
Rhexocercosporidium	Ploettnerulaceae	Ploettnerulaceae	exTypeSpecimen	from ITS R. carotae
Rhizocladosporium	Helotiales incertae sedis (2)	Helotiales incertae sedis (3)	exTypeSpecimen	
Rhizodermea	Dermateaceae	Dermateaceae	exTypeSpecimen	
Rhizoscyphus	Hyaloscyphaceae	Hyaloscyphaceae	TypeSpecies	= Hyaloscypha, Fehrer et al. 2019
Rhymbocarpus	Cordieritidacaeae	Cordieritidacaeae		
Rhynchosporium	Ploettnerulaceae	Ploettnerulaceae	TypeSpecies	
Rodwayella	Pezizellaceae	Pezizellaceae (3)	TypeSpecies	
**Roesleria**	**Roesleriaceae**	**Helotiaceae**	**TypeSpecies**	
Rommelaarsia	Helotiales incertae sedis (2)	Helotiales (Stamnaria lineage/Han Clade 9)	exTypeSpecimen	
Roseodiscus	Helotiales incertae sedis	Helotiales (Stamnaria lineage/Han Clade 9)	TypeSpecies	
Rutstroemia	Rutstroemiaceae	Rutstroemiaceae	TypeSpecies	
Sabahriopsis	Helotiales incertae sedis (2)	Cordieritidacaeae (3)	exTypeSpecimen	
**Satchmopsis**	**Pezizomycotina incertae sedis (2)**	**Leotiales incertae sedis (3)**	**TypeSpecies**	
**Scleromitrula**	**Sclerotiniaceae**	**Rutstroemiaceae (3)**	**TypeSpecies**	
Scleropezicula	Pezizellaceae	Pezizellaceae (3)	TypeSpecies	
Sclerotinia	Sclerotiniaceae	Sclerotiniaceae	TypeSpecies	
Scytalidium	Leotiomycetes incertae sedis (2)	Helotiales incertae sedis	exTypeSpecimen (4)	ITS, sister to Xylogone
Solenopezia	Lachnaceae	Lachnaceae (3)	TypeSpecies	
Soosiella	Helotiales incertae sedis (2)	Helotiales (Han Clade 4) (3)	exTypeSpecimen	
Spathularia	Cudoniaceae	Cudoniaceae	TypeSpecies	
Sphaerographium	Chaetomellaceae	Chaetomellaceae (3)		
**Spirosphaera**	**Helotiales incertae sedis (2)**	**Helotiaceae**		**"Hymenoscpyhus" varicosporioides clade**
Stamnaria	Stamnaria lineage	Helotiales (Stamnaria lineage/Han Clade 9)		
Streptotinia	Sclerotiniaceae	Sclerotiniaceae (3)		as Streptobotrys, anamorph of Streptotinia
Stromatinia	Sclerotiniaceae	Sclerotiniaceae (3)	TypeSpecies	
Strossmayeria	Strossmayeria lineage	Strossmayeria lineage		
Synchaetomella	Chaetomellaceae	Chaetomellaceae (3)		
Tapesia	Mollisiaceae	Mollisiaceae	TypeSpecies	
Teberdinia	Pseudeurotiaceae	Pseudeurotiaceae (3)	exTypeSpecimen	Pseudeurotium anamorph
Terriera	Rhytismataceae	Rhytismataceae		
Tetrachaetum	Discinella-Pezoloma lineage	Discinella-Pezoloma lineage (3)	TypeSpecies	
Tetracladium	Helotiales incertae sedis	Helotiales (Stamnaria lineage/Han Clade 9)	TypeSpecies	
Thamnogalla	Cordieritidaceae	Cordieritidaceae (3)	TypeSpecies	
Thedgonia	Drepanopezizaceae	Drepanopezizaceae (3)	TypeSpecies	
Thelebolus	Thelebolaceae	Thelebolaceae	TypeSpecies	
Thuemenidium	Leotiaceae	Leotiaceae		
**Tiarosporella**	**Phacidiaceae**	**Leotiomycetyes incertae sedis (3)**	**exTypeSpecimen**	
Torrendiella	Rutstroemiaceae	Rutstroemiaceae (3)	TypeSpecies	
Trichopeziza	Lachnaceae	Lachnaceae	TypeSpecies	

genus	current family (1, 2)	accepted family (3)	type status of specimens sequenced (4)	note
Trichopezizella	Lachnaceae	Lachnaceae	TypeSpecies (4)	
Tricladium	Helotiaceae (2)	Helotiaceae	TypeSpecies	"Hymenoscpyhus" varicosporioides clade
Trimmatostroma	Mollisiaceae	Mollisiaceae (3)	TypeSpecies	
**Trizodia**	**Mniaecia lineage**	**Leotiomycetes incertae sedis**	**TypeSpecies**	
Trochila	Cenangiaceae	Cenangiaceae	TypeSpecies	
Tromeropsis	Leotiomycetes incertae sedis	Leotiomycetes incertae sedis (3)		
Tryblidiopsis	Rhytismataceae	Rhytismataceae	TypeSpecies	
Tympanis	Tympanidaceae	Tympanidaceae		
**Unguicularia**	**Hyaloscyphaceae**	**Helotiales incertae sedis (3)**	**TypeSpecies**	
**Urceolella**	**Hyaloscyphaceae**	**Helotiales (Stamnaria lineage/Han Clade 9)**	**TypeSpecies**	
Valdensinia	Sclerotiniaceae	Sclerotiniaceae (3)	TypeSpecies	
Vandijckella	Vandijckellaceae (2)	Vandijckellaceae	exTypeSpecimen	
**Varicosporium**	**Helotiaceae (2)**	**Discinella-Pezoloma lineage**	**TypeSpecies**	
Variocladium	Mollisiaceae	Mollisiaceae (3)		
Velutarina	Cenangiaceae	Cenangiaceae (3)	TypeSpecies	
**Venturiocistella**	**Hyaloscyphaceae**	**Helotiales (Han Clade 4)**		
Verpatinia	Sclerotiniaceae	Sclerotiniaceae (3)	TypeSpecies	
Vibrissea	Vibrisseaceae	Vibrisseaceae	TypeSpecies	
**Xenopolyscytalum**	**Helotiales incertae sedis (2)**	**Pezizellaceae (3)**	**exTypeSpecimen**	**Han Clade 1 subclade**
Xerombrophila	Gelatinodiscaceae	Gelatinodiscaceae	TypeSpecies	
Xeropilidium	Chaetomellaceae	Chaetomellaceae	exTypeSpecimen	
Xylogone	Helotiales incertae sedis (2)	Helotiales incertae sedis (3)	exTypeSpecimen	
Ypsilina	Ploettnerulaceae	Ploettnerulaceae (3)	TypeSpecies	
**Zymochalara**	**Pezizomycotina incertae sedis (2)**	**Pezizellaceae (3)**	**exTypeSpecimen**	
