# Supplementary material for: A multigene phylogeny toward a new phylogenetic classification of Leotiomycetes
Source: IMA Fungus. 2019 Jun 7;10:1. doi: 10.1186/s43008-019-0002-x (PMC7325659; doi:10.1186/s43008-019-0002-x)
Supplement: Supplementary file 8 — Table S4. Basic data on the draft genome assemblies of 10 Leotiomycetes specimens deposited as NCBI Bioproject PRJNA487672. Data compares results from two assemblers. (DOCX 19 kb) [file 43008_2019_2_MOESM8_ESM.docx]

**Additional file 8: Table S4**. Basic data on the draft genome assemblies of 10 Leotiomycetes specimens deposited as NCBI Bioproject PRJNA487672. Data compares results from two assemblers.

| 1. **Platanus assembler** | |  | | | |  | | |  | | |  | | | |  | | |  | | |  | | |  | | |
| --- | --- | --- | --- | --- | --- | --- | --- | --- | --- | --- | --- | --- | --- | --- | --- | --- | --- | --- | --- | --- | --- | --- | --- | --- | --- | --- | --- |
| **Code** | **Voucher** | | **Species** | | | | **Average Coverage** | | **Genome Size** | | **Genome size after QC (1)** | | | **GC content (%)** | | | **Number of scaffolds** | | **Number of scaffolds after QC** | | **Max sequence length** | | | **N50** | | | **Unique Genes (3)** |
| D781 | ICMP 21731 | | *Arachnopeziza araneosa* | | | | 128 | | 41,230,740 | | 39,684,587 | | | 45.92 | | | 13,209 | | 2,459 | | 268,785 | | | 53,417 | | | 21,358 |
| D1686 | ICMP 21732 | | *Chlorencoelia torta* | | | | 130 | | 49,115,237 | | 40,661,169 | | | 40.78 | | | 69,713 | | 6,764 | | 181,972 | | | 32,563 | | | 15,588 |
| D1086 | ICMP 22793 | | *Hymenotorrendiella dingleyae* | | | | 82 | | 59,899,713 | | 54,762,223 | | | 43.75 | | | 51,616 | | 7,203 | | 90,342 | | | 17,811 | | | 25,356 |
| D1413 | ICMP 21723 | | *Hyphodiscus* sp. | | | | 140 | | 35,238,611 | | 34,094,528 | | | 47.49 | | | 10,369 | | 1,669 | | 422,475 | | | 91,689 | | | 19,548 |
| D664 | ICMP 21728 | | *Lachnum nothofagi* | | | | 57 | | 70,391,041 | | 57,394,826 | | | 43.18 | | | 78,699 | | 6,885 | | 850,822 | | | 44,455 | | | 31,741 |
| D365 | ICMP 21729 | | *"Mollisia"* sp. (*Cadophora* clade) | | | | 147 | | 32,392,701 | | 31,994,671 | | | 46.92 | | | 2,706 | | 305 | | 1,103,264 | | | 269,068 | | | 14,692 |
| D728 | ICMP 21725 | | *Phialocephala sp.* | | | | 87 | | 50,699,329 | | 49,378,803 | | | 45.78 | | | 8,079 | | 911 | | 886,120 | | | 265,667 | | | 25,792 |
| D2031 | ICMP 18395 | | *Neobulgaria alba* | | | | 119 | | 41,828,152 | | 37,752,759 | | | 44.51 | | | 34,679 | | 4,265 | | 214,504 | | | 30,117 | | | 18,047 |
| D792 | ICMP 13383 | | *“Pirottaea” palmicola* | | | | 108 | | 55,082,384 | | 38,795,366 | | | 46.51 | | | 109,472 | | 1,516 | | 542,459 | | | 111,983 | | | 18,961 |
| D683 | ICMP 21730 | | *Proliferodiscus dingleyae* | | | | 142 | | 34,061,436 | | 33,986,942 | | | 48.10 | | | 1,195 | | 744 | | 348,158 | | | 95,482 | | | 19,186 |
|  |  | |  | | | |  | |  | |  | | |  | | |  | |  | |  | | |  | | |  |
| 1. **A5 MiSeq assembler** | | | |  |  | | |  | |  | | |  | |  | | |  | |  | | |  | | |  | |
| **Code** | **Voucher** | | **Species** | | | | **Average Coverage** | | **Genome Size** | | **Genome size after QC (2)** | | | **GC content (%)** | | | **Number of scaffolds** | | **Number of scaffolds after QC** | | **Max sequence length** | | | **N50** | | | **Unique Genes (3)** |
| D781 | ICMP 21731 | | *Arachnopeziza araneosa* | | | | 135 | | 40,453,245 | | 40,284,875 | | | 45.69 | | | 1,498 | | 992 | | 1,164,973 | | | 210,669 | | | 21,253 |
| D1686 | ICMP 21732 | | *Chlorencoelia torta* | | | | 137 | | 44,380,449 | | 43,501,201 | | | 38.78 | | | 6,881 | | 4,449 | | 1,733,051 | | | 325,507 | | | 15,303 |
| D1086 | ICMP 22793 | | *Hymenotorrendiella dingleyae* | | | | 90 | | 56,478,799 | | 56,111,224 | | | 43.70 | | | 3,867 | | 2,808 | | 3,335,922 | | | 69,680 | | | 25,167 |
| D1413 | ICMP 21723 | | *Hyphodiscus* sp. | | | | 148 | | 34,520,837 | | 34,380,922 | | | 47.42 | | | 1,074 | | 658 | | 1,489,265 | | | 538,937 | | | 19,455 |
| D664 | ICMP 21728 | | *Lachnum nothofagi* | | | | 65 | | 76,660,889 | | 73,808,206 | | | 40.60 | | | 26,077 | | 18,310 | | 830,123 | | | 10,582 | | | 42,655 |
| D365 | ICMP 21729 | | *"Mollisia"* sp. (*Cadophora* clade) | | | | 156 | | 32,146,331 | | 32,093,364 | | | 46.92 | | | 485 | | 323 | | 806,231 | | | 251,042 | | | 14,728 |
| D728 | ICMP 21725 | | *Phialocephala sp.* | | | | 95 | | 50,315,576 | | 50,231,846 | | | 45.50 | | | 1,295 | | 1,044 | | 1,151,817 | | | 192,801 | | | 26,031 |
| D2031 | ICMP 18395 | | *Neobulgaria alba* | | | | 125 | | 39,586,693 | | 39,320,073 | | | 43.66 | | | 2,641 | | 1,889 | | 1,099,189 | | | 268,321 | | | 17,670 |
| D792 | ICMP 13383 | | *“Pirottaea” palmicola* | | | | 108 | | 50,288,663 | | 44,723,096 | | | 45.03 | | | 24,080 | | 8,935 | | 277,300 | | | 58,028 | | | 20,016 |
| D683 | ICMP 21730 | | *Proliferodiscus dingleyae* | | | | 153 | | 34,013,099 | | 34,010,436 | | | 48.11 | | | 70 | | 61 | | 2,245,879 | | | 929,377 | | | 19,052 |

(1) using platanus_trim (http://platanus.bio.titech.ac.jp/pltanus_trim) to remove adaptor sequences and low quality regions

(2) using Trimmomatic (Bolger et al. 2014) to remove adaptor sequences and low quality reads.

(3) estimated using GlimmerHMM as implemented in QUAST (Gurevich et al. 2013)

(4) estimated using Busco v3 (Waterhouse et al. 2017)
