# Supplementary material for: A multigene phylogeny toward a new phylogenetic classification of Leotiomycetes
Source: IMA Fungus. 2019 Jun 7;10:1. doi: 10.1186/s43008-019-0002-x (PMC7325659; doi:10.1186/s43008-019-0002-x)
Supplement: Supplementary file 9 — Table S5. Number of genes detected using BUSCO for each of the genomes used in genome phylogeny. Completeness value based on the BUSCO Pezizomycotina database (contains 3156 reference genes). (DOCX 19 kb) [file 43008_2019_2_MOESM9_ESM.docx]

**Additional file 9: Table S5**. Number of genes detected using BUSCO for each of the genomes used in genome phylogeny. Completeness value based on the BUSCO Pezizomycotina database (contains 3,156 reference genes).

| **Code** | **Voucher** | **Species** | **JGI label or NCBI biosample** | **Completeness** | **Complete genes** | **Complete single-copy** | **Duplicate genes** | **Fragmented genes** | **Missing genes** |
| --- | --- | --- | --- | --- | --- | --- | --- | --- | --- |
| Amore1 | ATCC 22711 | *Amorphotheca resinae* | Amore1 | 98.9% | 3119 | 3117 | 2 | 14 | 23 |
| D781 | ICMP 21731 | *Arachnopeziza araneosa* | SAMN09907757 | 98.1% | 3049 | 3085 | 9 | 21 | 41 |
| Ascsa1 | NRRL 50072 | *Ascocoryne sarcoides* | Ascsa1 | 98.0% | 3095 | 3088 | 7 | 17 | 44 |
| Bisps1 | PMI 857 | *Calycina* sp. (as *Bisporella* sp.) | Bisps1 | 96.5% | 3043 | 2956 | 87 | 37 | 76 |
| Blugr1 | DH14 | *Blumeria graminis* f.sp. *hordei* | Blugr1 | 88.9% | 2805 | 2790 | 15 | 95 | 256 |
| Botci1 | B05.10 | *Botrytis cinerea* | Botci1 | 98.6% | 3112 | 3106 | 6 | 9 | 35 |
| Buln1 | CBS 118.31 | *Bulgaria inquinans* | Bulin1 | 98.0% | 3093 | 3089 | 4 | 26 | 37 |
| Cadsp1 | DSE1049 | *Cadophora sp.* | Cadsp1 | 99.1% | 3129 | 3103 | 26 | 7 | 20 |
| VPRI42388 | FRR 6070 | *Cairneyella variabilis* | SAMN04263868 | 98.2% | 3098 | 3083 | 15 | 25 | 33 |
| Chalo1 | BDJ | *Chalara longipes* | Chalo1 | 98.7% | 3116 | 3103 | 13 | 20 | 20 |
| D1686 | ICMP 21732 | *Chlorencoelia torta* | SAMN09907751 | 96.8% | 3056 | 3046 | 10 | 37 | 63 |
| IHIA39 | IHIA39 | *Chlorociboria aeruginascens* | SAMN06706673 | 96.9% | 3049 | 3039 | 10 | 40 | 67 |
| ICMP19812 | ICMP 19812 | *Ciborinia camelliae* | SAMN03840770 | 95.7% | 3021 | 3014 | 7 | 54 | 81 |
| Cocst1 | CBS 202.91 | *Coccomyces strobi* | Cocst1 | 96.7% | 3052 | 3049 | 3 | 27 | 77 |
| DortE4 | DortE4 | *Diplocarpon rosae* | SAMN06163654 | 91.7% | 3065 | 869 | 2196 | 34 | 57 |
| Erynec1 | strain c | *Erysiphe necator* | Erynec1 | 85.8% | 2707 | 2705 | 2 | 127 | 322 |
| Glalo1 | ATCC 20868 | *Glarea lozoyensis* | Glalo1 | 98.6% | 3112 | 3102 | 10 | 20 | 24 |
| Golci1 | UCSC1 | *Golovinomyces cichoracearum* | Golci1 | 54.8% | 1728 | 1704 | 24 | 269 | 1159 |
| Greab1 | DAOM 170408 | *Gremmeniella abietina* | Greab1 | 98.1% | 3095 | 3092 | 3 | 17 | 44 |
| Hymvar1 |  | *Hymenoscyphus varicosporoides* | Hymvar1 | 98.2% | 3102 | 3091 | 11 | 14 | 40 |
| D1086 | ICMP 22793 | *Hymenotorrendiella dingleyae* | SAMN09907749 | 98.3% | 3102 | 3097 | 5 | 25 | 29 |
| D1413 | ICMP 21723 | *Hyphodiscus sp.* | SAMN09907750 | 98.2% | 3100 | 3090 | 10 | 19 | 37 |
| D664 | ICMP 21728 | *Lachnum nothofagi* | SAMN09907754 | 90.1% | 2842 | 2733 | 109 | 198 | 120 |
| Leptod1 | PMI412 | *Leptodontidium sp.* | Leptod1 | 99.2% | 3130 | 3095 | 35 | 9 | 17 |
| Lorju1 | ATCC 46458 | *Loramyces juncicola* | Lorju1 | 98.5% | 3109 | 3102 | 7 | 20 | 27 |
| Lorma1 | CBS 235.53 | *Loramyces macrosporus* | Lorma1 | 98.2% | 3097 | 3092 | 5 | 23 | 36 |
| Marbr1 |  | *Marssonina brunnea* | Marbr1 | 98.9% | 3123 | 3119 | 4 | 9 | 24 |
| NL1 | NL1 | *Diplocarpon mali* (as *Marssonina coronariae)* | SAMN06564146 | 97.9% | 3089 | 3085 | 4 | 32 | 35 |
| Melbi2 |  | *Meliniomyces bicolor* | Melbi2 | 98.8% | 3118 | 3096 | 22 | 15 | 23 |
| Melva1 |  | *Meliniomyces variabilis* | Melva1 | 99.0% | 3126 | 3109 | 17 | 12 | 18 |
| D365 | ICMP 21729 | *'Mollisia'* sp. *Cadophora* clade | SAMN09907753 | 98.6% | 3115 | 3106 | 6 | 11 | 33 |
| LMK759 | LMK759 | *Mycrosclerotinia curreyana* | SAMN03448624 | 97.8% | 3084 | 3079 | 5 | 20 | 52 |
| D2031 | ICMP 18395 | *Neobulgaria alba* | SAMN09907752 | 98.1% | 3095 | 3086 | 9 | 17 | 44 |
| OR74A | OR74A | *Neurospora crassa* | Neucr2 | 98.1% | 3094 | 3092 | 2 | 24 | 38 |
| Oidma1 |  | *Oidiodendron maius* | Oidma1 | 98.0% | 3095 | 3056 | 39 | 25 | 36 |
| NRRL12192 | NRRL 12192 | *Pezicula radicicola* | SAMN07709029 | 98.8% | 3120 | 3092 | 28 | 16 | 20 |
| UAMH11012 | UAMH 11012 | *Phialocepha subalpina* | PRJEB12348 | 98.4% | 3104 | 3079 | 25 | 19 | 33 |
| Phisc1 | CBS 120377 | *Phialocephala scopiformis* | Phisc1 | 98.9% | 3122 | 3112 | 10 | 13 | 21 |
| D728 | ICMP 21725 | *Phialocephala sp.* | SAMN09907756 | 98.8% | 3118 | 3113 | 5 | 15 | 23 |
| D792 | ICMP 13383 | *‘Pirottaea’ palmicola* | SAMN09907758 | 98.3% | 3101 | 3096 | 5 | 25 | 30 |
| D683 | ICMP 21730 | *Proliferodiscus dingleyae* | SAMN09907755 | 98.2% | 3101 | 3094 | 7 | 20 | 35 |
| Pseudest | 20631-21 | *Pseudogymnoascus destructans* | Pseudest1 | 97.1% | 3064 | 3045 | 19 | 26 | 66 |
| Rhesp1 | MPI-PUGE-AT-0058 | *Rhexocercosporidium* sp. | Rhesp1 | 98.8% | 3119 | 3106 | 13 | 9 | 28 |
| Rhier1 | UAMH 7357 | *Rhizoscyphus ericae* | Rhier1 | 99.0% | 3125 | 3115 | 10 | 9 | 22 |
| UK7 | UK7 | *Rhynchosporium commune* | SAMEA3895816 | 98.8% | 3118 | 3110 | 8 | 14 | 24 |
| Rutfi1 | CBS 115.86 | *Rustroemia firma* | Rutfi1 | 98.2% | 3100 | 3097 | 3 | 19 | 37 |
| CBS115975 | CBS 115975 | *Rustroemia sydowiana* | SAMN02903521 | 86.9% | 2741 | 2610 | 131 | 248 | 167 |
| CBS111548 | CBS 111548 | *Rutstroemia echinophila* | SAMN02903859 | 80.2% | 2531 | 2526 | 5 | 360 | 265 |
| Sclsc1 | ATCC 18683 | *Sclerotinia sclerotiorum* | Sclsc1 | 96.7% | 3050 | 3045 | 5 | 46 | 60 |
| Themi | ATCC 90970 | *Thelebolus microsporus* | Themi1 | 95.8% | 3023 | 3013 | 10 | 38 | 95 |
| AFTOL_ID_51 | OSC 100004 | *Xylaria hypoxylon* | Xylhyp1 | 97.8% | 3083 | 3079 | 7 | 24 | 46 |
